# LncRNAs of *Saccharomyces cerevisiae* bypass the cell cycle arrest imposed by ethanol stress

**DOI:** 10.1371/journal.pcbi.1010081

**Published:** 2022-05-19

**Authors:** Lucas Cardoso Lázari, Ivan Rodrigo Wolf, Amanda Piveta Schnepper, Guilherme Targino Valente

**Affiliations:** 1 Department of Parasitology, Institute of Biomedical Sciences, Sāo Paulo University (USP), Sao Paulo, Brazil; 2 Department of Bioprocess and Biotechnology, School of Agriculture, Sao Paulo State University (UNESP), Botucatu, Brazil; 3 Department of Structural and Functional Biology, Institute of Bioscience at Botucatu, Sao Paulo State University (UNESP), Botucatu, Brazil; 4 Max Planck Institute for Heart and Lung Research, Bad Nauheim, Germany; Pázmány Péter Catholic University: Pazmany Peter Katolikus Egyetem, HUNGARY

## Abstract

Ethanol alters many subsystems of *Saccharomyces cerevisiae*, including the cell cycle. Two ethanol-responsive lncRNAs in yeast interact with cell cycle proteins, and here, we investigated the role of these RNAs in cell cycle. Our network dynamic modeling showed that higher and lower ethanol-tolerant strains undergo cell cycle arrest in mitosis and G1 phases, respectively, during ethanol stress. The higher population rebound of the lower ethanol-tolerant phenotype after stress relief responds to the late phase arrest. We found that the lncRNA lnc9136 of SEY6210 (a lower ethanol-tolerant strain) induces cells to skip mitosis arrest. Simulating an overexpression of lnc9136 and analyzing CRISPR–Cas9 mutants lacking this lncRNA suggest that lnc9136 induces a regular cell cycle even under ethanol stress, indirectly regulating Swe1p and Clb1/2 by binding to Gin4p and Hsl1p. Notably, lnc10883 of BY4742 (a higher ethanol-tolerant strain) does not prevent G1 arrest in this strain under ethanol stress. However, lnc19883 circumvents DNA and spindle damage checkpoints, maintaining a functional cell cycle by interacting with Mec1p or Bub1p even in the presence of DNA/spindle damage. Overall, we present the first evidence of direct roles for lncRNAs in regulating yeast cell cycle proteins, the dynamics of this system in different ethanol-tolerant phenotypes, and a new yeast cell cycle model.

## Author summary

Ethanol is a cell stressor in yeast that dampen ethanol production. LncRNAs are RNAs that control many cellular processes. Computational simulations allow us to study the dynamism of cell systems. Therefore, we built a computational model of the yeast cell cycle to investigate how cells respond to ethanol stress. Simulations showed that ethanol stress or spindle damage arrests the cell cycle. Furthermore, the performance of higher and lower ethanol-tolerant strains in poststress recovery growth seems to be related to the cell cycle phase in which cells are stalled. However, two lncRNAs maintain the activity of the cell cycle even in yeast cells under these stresses by repressing specific cell cycle proteins. Finally, this model facilitates analyses of the yeast cell cycle for applied or basic science purposes.

## Introduction

Global ethanol production depends on the use of *Saccharomyces cerevisiae* [1,2]. However, the high ethanol yield challenges production, stalling fermentation. Ethanol stress alters many yeast pathways, reducing yeast viability, cell growth, macromolecule biosynthesis, fermentation, and membrane integrity [3,4]. The yeast ethanol stress-responsive mechanisms and cell surveillance comprise the regulation of many specific genes affecting a wide range of biological processes, especially the cell cycle and viability pathways [5–8]. For example, the activity of several cell cycle-related genes (e.g., BUB1, CDH1, CLN3, SWE1, GRR1, SWI4, and SWI6) is crucial to maintain yeast cell growth in a medium containing 11% ethanol (volume/volume) [9]. Altogether, knowledge concerning the dynamics of cell cycle systems under stressful conditions is essential to develop new ethanol-tolerant strains.

Cyclins activate kinases to drive the whole cell cycle. Transcription factors activated in G1 phase induce the cell cycle, in which cells reach constant growth and protein synthesis, leading to the activation of Cln1p, Cln2p, or Cln3p cyclins. Activated cyclins bind to Cdc28p kinase, activating the transcription factors SBF and MBF, which control the transcription of essential genes for subsequent phases [10]. The DNA is replicated via Clb5p and Clb6p type B cyclins in S phase. Furthermore, Clbs and the conjoint action of the pre-replication complex are essential for proper DNA unpacking and recruitment of polymerases [11–13]; then, cells may be ready to advance to G2 phase. After duplication of spindle pole bodies by the action of Clbs proteins (Clb1p, Clb2p, Clb3p, and Clb4p), the activation of cyclin inhibitors is crucial to degrade Clbs, triggering completion of anaphase [10]. Throughout the phases, checkpoint mechanisms stop cycle progression until cells reach an appropriate size, repair DNA damage, complete kinetochore attachment and spindle alignment, and occasionally wait until the end of the mating process [14–17].

Long noncoding RNAs (lncRNAs) are >200 nucleotides in length and function in many regulatory processes [18–22]. LncRNAs may scaffold protein complexes or regulate gene expression by interacting with chromatin enhancer-related proteins or transcription factors [23]. We previously identified ethanol stress-responsive lncRNAs in six yeast strains. Furthermore, we showed that the cell cycle is a pathway that is substantially affected by ethanol stress [24].

Most of the knowledge about the eukaryotic cell cycle is based on the study of yeast systems [25]. However, the yeast cell cycle dynamics under ethanol stress, the role of lncRNAs in this process, and the interactions between lncRNAs and cell cycle proteins are unknown. We addressed these points using dynamic network modeling, population rebound experiments, and CRISPR–Cas9 approaches. We developed a logic model of the yeast cell cycle to assess the effect of ethanol stress and the molecular mechanisms of lnc9136 and lnc10883 on cell cycle progression. We used transcriptome data from lower (LT) and higher (HT) ethanol-tolerant strains under severe ethanol stress to perform dynamic network modeling. The computational simulation suggests that both phenotypes undergo cell cycle arrest during extreme ethanol stress: the LT arrests in a later cell cycle stage (M phase) than the HT (G1 phase). The late cell cycle phase arrest in LT reported by simulations may be related to their faster population rebound after ethanol stress relief observed in our analysis of the population rebound experiment. Remarkably, the *in silico* overexpression of the lncRNA lnc9136 in SEY6210 (an LT strain) reported a bypass of M phase arrest by repressing two proteins, allowing the cell cycle release in this strain even under ethanol stress; partial deletions of this lncRNA by CRISPR–Cas9 corroborated this finding. Finally, the simulations also allowed us to associate the lncRNA lnc10883 of BY4742 (an HT strain) with the release of cell cycle arrest, although cells likely present DNA or spindle damage.

## Results

### Overview

**Fig 1:**
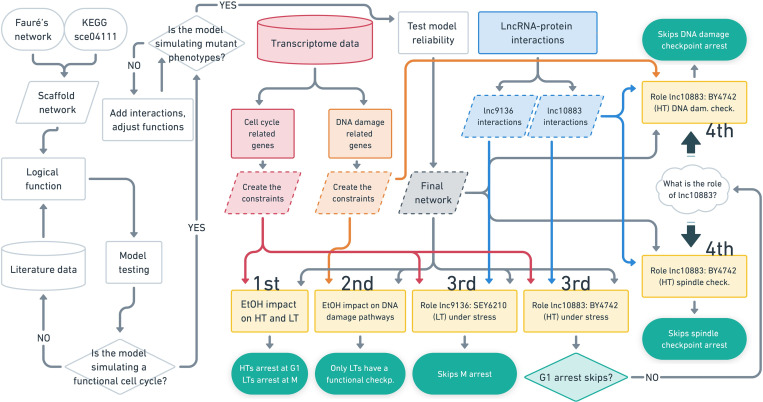
Workflow of model construction (left and central white forms), experimental model simulations to study the effects of ethanol stress and lncRNAs on the yeast cell cycle (yellow forms), and simulation results (green forms). ’Test model reliability’ includes simulations of the model with several random cell cycle perturbations and the effect of checkpoint nodes on cell cycle arrest. ’EtOH’ indicates ethanol, ’HT’ and ’LT’ are higher and lower ethanol-tolerant phenotypes, respectively. Details concerning the methods and the description of experimental model simulations (ranging from 1^st^ to 4^th^ in the figure) are described in the Materials and Methods section.

**Fig 2:**
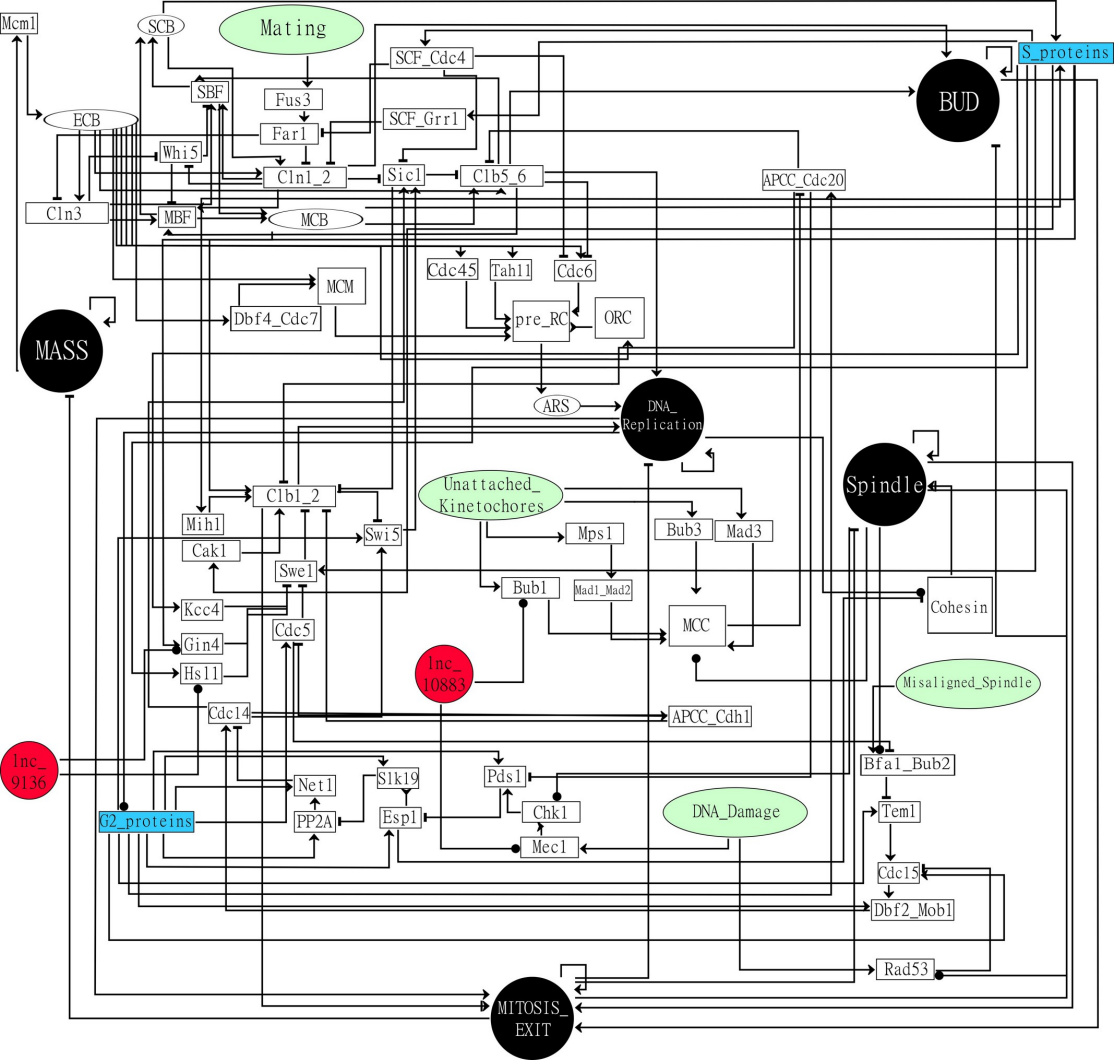
Network model. Black circles are phenomenological nodes, white circles are genomic regulatory elements, green circles are checkpoint nodes, red circles are lncRNA nodes, white rectangles are protein or protein complex nodes, and blue rectangles are the S_proteins and G2_proteins nodes responsible for activating nodes from S and G2 phases, respectively. The lncRNA-protein interactions were predicted independently for the SEY6210 and BY4742 strains. The computational GINsim model is available at https://figshare.com/articles/software/Yeast_Cell_Cycle_Logic_Model/14503035.

We developed a new computational model for the yeast cell cycle integrating a logic model already available [26], part of the KEGG yeast cell cycle pathway (accession number sce04111) [27], and data from the literature. Our model was continuously adjusted until it presented a marked improvement in performance in predicting most mutants with disrupted cell cycle phenotypes selected from the literature (hereafter referred to as cell cycle mutants); these results were also used to assess the model performance (**Fig 1**). Thus, the final network used here has 67 nodes and 144 interactions. The nodes represent proteins, protein complexes (e.g., the pre-replication complex, represented by the pre_RC node), phenomenological nodes (e.g., the MASS node), checkpoint nodes (e.g., the DNA_Damage node), genomic regulatory elements (e.g., ARS), and our predicted lncRNA-protein interactions (**Fig 2**). The model simulated several cell cycle perturbations to assess the model reliability. Finally, we also emulated the effect of checkpoint nodes on cell cycle arrest (**Fig 1**).

The node state in Boolean networks may be ’0’ (representing inactivity or absence) or ’1’ (representing activity or presence). However, we generated our logic model with multivalue nodes to improve the simulation accuracy by modifying the logical function described [26] or creating new functions for most nodes. Thus, most nodes in our model assume values of ’0’, ’1’, ’2’ or ‘3’ (**Fig 1 and S1 Table**); the biological meaning of each value depends on the type of node (**Table 1**).

**Table 1:** Node types, range of values, and their biological meaning. ’a’ indicates that this node type also has some nodes that reach only three values. Thus, ’1’ and ’2’ indicate normal and high levels (or activity), respectively. ’b’, the checkpoint node Mating is a Boolean node. Then, ’0’ or ’1’ indicate inactivation or activation of the Mating node, respectively. ’c’ refers to the lncRNAs or selected genes for the experimental model simulations (details provided in the ‘Simulating the effects of ethanol stress-responsive lncRNAs and ethanol on the cell cycle’ in the Materials and Methods section).

Node type	Node values range	Meaning of respective values
Protein^a^	’0’, ’1’, ’2’ or ’3’	Absence, low, normal or high protein yield
Protein complex^a^	’0’, ’1’, ’2’ or ’3’	Inactive, low, normal or high activation level
MASS	’0’, ’1’ or ’2’	Pre-mass increase, starts the mass increasing, or maximum mass level
BUD	’0’, ’1’ or ’2’	Absence, activation of bud formation, or bud presence
DNA_Replication	’0’ or ’1’	DNA not replicated or DNA replication
Spindle	’0’, ’1’ or ’2’	Absent, activation of spindle formation, or spindle presence
MITOSIS_EXIT	’0’, ’1’ or ’2’	Interphase, M phase entering, or mitotic exit
Checkpoint^b^	’0’, ’1’ or ’2’	Inactive, active but not causing arrest, or active causing arrest
Genes^c^	’0’, ’1’, ’2’ or ’3’	Non-differential expression, downregulation, normal expression, or upregulation
Genomic regulatory elements	’0’, ’1’, ’2’ or ’3’	Inactive, low, normal or high activity

We used our model to emulate the cell cycle without any perturbation (hereafter referred to as the regular cell cycle) (**S1 Fig**), to analyze the cell cycle under ethanol stress and the role of two lncRNAs in this pathway, as well as spindle and DNA damage checkpoints. Thus, we performed four experimental model simulations. The first experimental model simulation showed that the higher and lower ethanol-tolerant strains (HT and LT strains, respectively) exhibited cell cycle arrest under ethanol stress. The second experimental model simulation showed that all LTs had a functional checkpoint related to DNA damage under ethanol stress. The third model simulation showed that the lnc10883 of BY4742 under ethanol stress did not avoid the cell cycle arrest reported in the first experimental model simulation. However, the fourth model simulation suggests that lnc10883 may skip activated DNA damage and spindle checkpoint arrest. In the first, second, and fourth simulations, we used the gene expression from transcriptome data of HT and LT strains at their highest ethanol level supported [24] in our logic equations to define specific model constraints (**Fig 1**).

### The model’s performance

The simulation of our model characterizes a functional cell cycle when if the state transition graph presents the activation of all phenomenological nodes (MASS, BUD, DNA_Replication, Spindle, and MITOSIS_EXIT) (node value ≥ 1), followed by their inhibition (mainly the MASS node) (node value = 0), and finally, the cell cycle restarting (**S1 Fig**). Otherwise, the model simulation shows cell cycle arrest (details in the ’Model cycling rationale’ in the Materials and Methods section).

**Fig 3:**
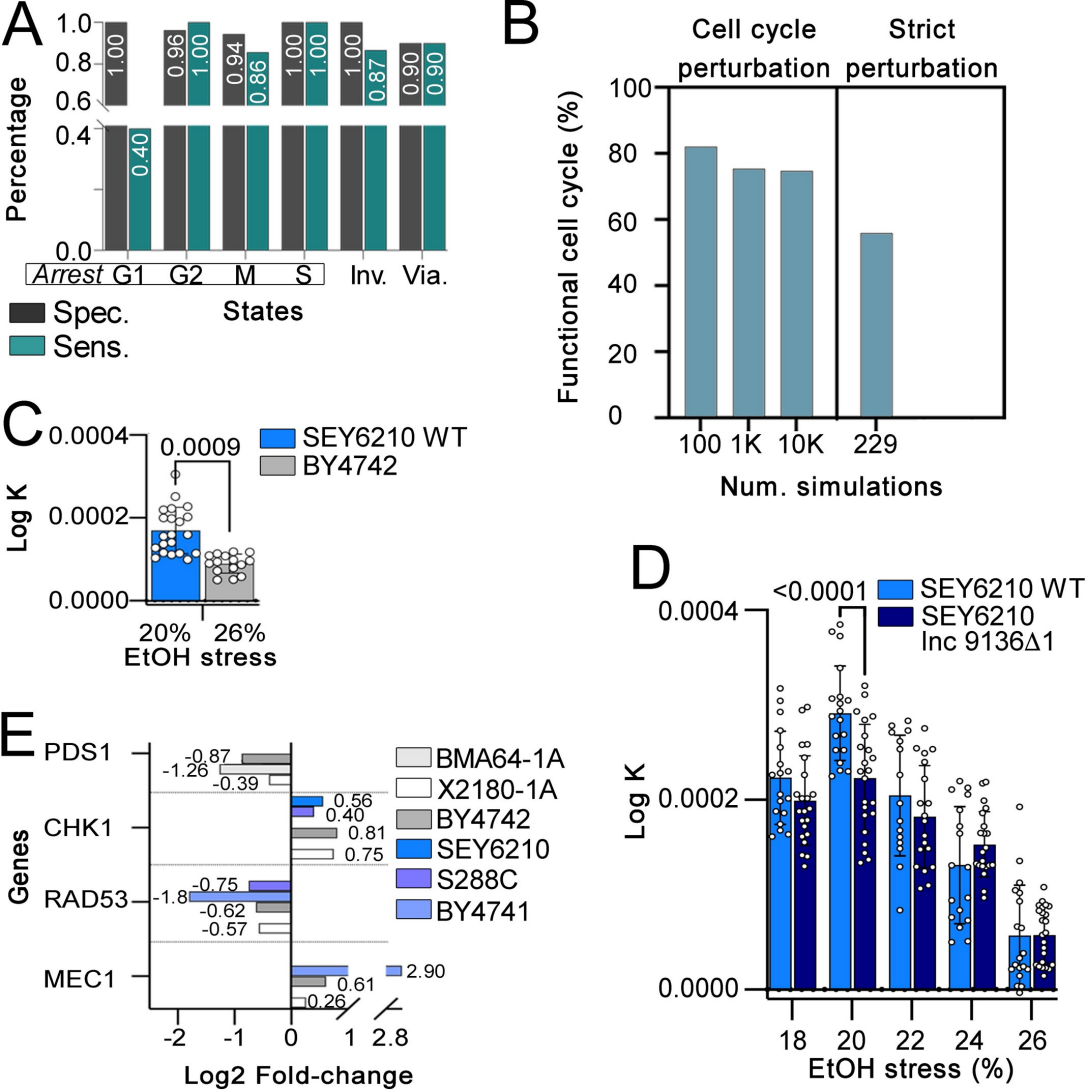
Model evaluation (A and B), growth curve analysis (C and D), and DNA damage-related gene expression (E). **A:** Specificity and sensitivity of simulating cell cycle mutants (mutants that had a disruption in the cell cycle) calculated by comparing the simulation outcomes and the reported description of each mutant. ’Inv.’ and ’Via’ are ’Inviable’ and ’Viable’ phenotypes, respectively; **B:** the percentage of functional cell cycles (details in the ’Model cycling rationale’ in the Materials and Methods section) from random cell cycle perturbation simulations (100, 1,000, and 10,000 simulations) and in the random perturbations from G1 phase. **C:** Population growth curve analysis of wild-type (WT) SEY6210 and BY4742 strains in the population rebound experiment after exposure to the highest ethanol stress level supported for each strain (’X’ axis). **D:** Population growth curve analysis in the population rebound experiment after ethanol (EtOH) stress relief (’X’ axis). The SEY6210 WT and SEY6210 lnc9136Δ1 mutant (partial deletion of lnc9136) strains were analyzed. **E:** Log_2_ fold-change (’X’ axis) in the expression of DNA damage-related genes between the treatment and control groups under the highest ethanol stress level for each strain [24]. The logic equations used to simulate these expressions as systems constraints are described in **S4 Table**. A higher populational growth rate (Log K in ’Y’ axis in ’C’ and ’D’) indicates the population with a higher ability to recover the growth rate after stress relief, and the number above bar indicates p values.

The model performance presented 86.6% accuracy in predicting the 109 cell cycle mutants and good sensitivity and specificity in predicting specific mutant types (’Inviable’, ’Viable’ and arrests in ’G1’, ’G2’, ’M’ and ’S’ phases), with the type of ’arrest in G1’ the only exception (**Fig 3A and S2 Table**). Many papers concerning an *in vivo* analysis and reporting the aforementioned mutant phenotypes do not accurately describe the exact cell cycle phase in which these mutants were arrested. For instance, if the publication reports a particular mutant as ’inviable’ but our model predicted a G1 arrest, we considered our result a correct prediction, and we labeled the mutant as ’Inviable’. Therefore, the sensitivity and specificity of some phenotypes (e.g., G1 arrest) are likely lower than expected.

We imposed random cell cycle perturbations in the model to assess its reliability. First, 100, 1,000 and 10,000 simulations of the model with random cell cycle perturbations showed 82%, 75.4% and 74.6% cyclic attractors passing through all cell cycle phases, respectively (**Fig 3B**). Interestingly, the 82, 754, and 7,469 largest basins of attractions from these simulations characterized functional cell cycle simulations. The remaining simulations (18%, 24.6%, and 25.4%) represented cell cycle arrests, usually with stable states in single state attractors (see examples in the **S1 Video**). Second, only 55.9% of 229 simulations of the model with random cell cycle perturbations ranging from G1 to mitotic exit phases characterized functional cell cycles (**Fig 3B**); the model set to start the simulation from G1 phase requires MASS, Cln3, Whi5, SBF, and MBF activation and inactivation of the DNA_Replication node.

The simulation of the model fixing at least one checkpoint node in the maximum activation level or all checkpoints at low/high activation, usually returned a cell cycle arrest. These simulations revealed the effect of checkpoint nodes on cell cycle arrest (**S3 Table**).

### The effect of ethanol stress on cell cycle progression

**Fig 4:**
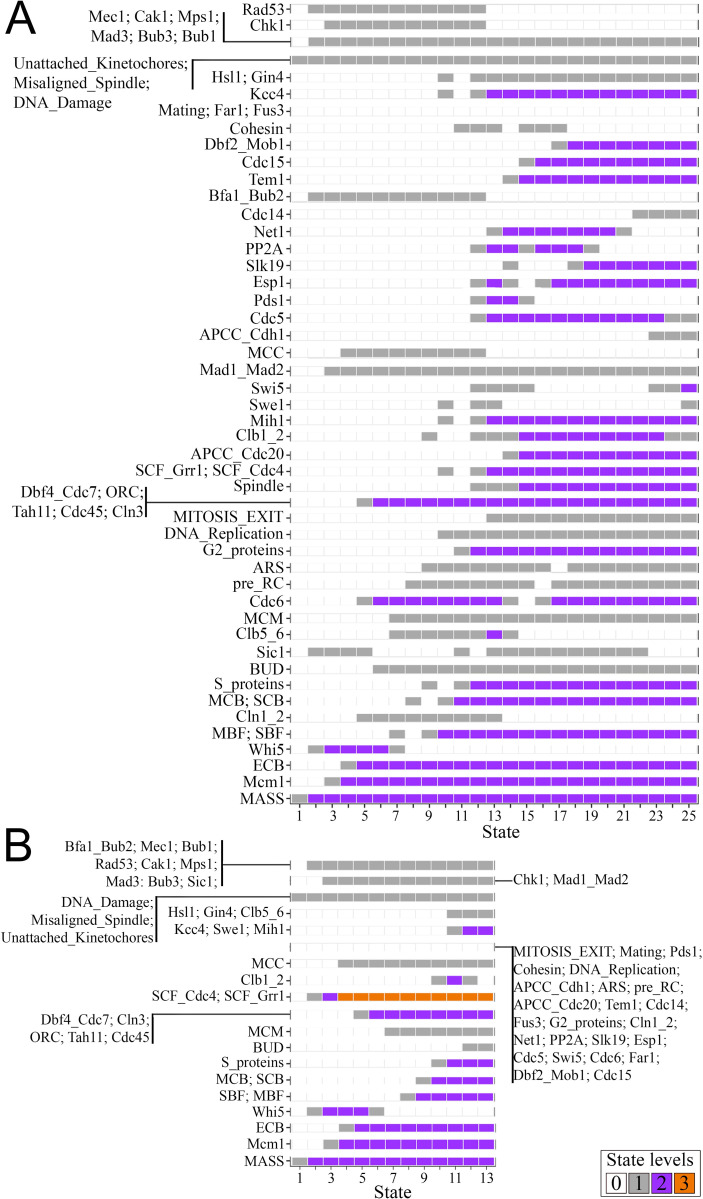
Cell cycle predictions of the LT (A) and HT (B) phenotypes under ethanol stress (the first experimental model simulation in **Fig 1**). The box color indicates the node values in each simulation state. Cell cycle arrest is defined when the simulation usually displays single-state attractors without the MASS node returning to ’0’, as observed here; otherwise, the simulation of the model returned a functional cell cycle. The M phase arrest in the LT (A) is evidenced here by MASS > ’0’ and MITOSIS_EXIT = ’1’. G1 arrest in the HT (B) is evidenced here by the MASS > ’0’, and DNA_Replication = ’0’. Details concerning arrests are reported in the ’Model cycling rationale’ and **Eq 1** in the Materials and Methods section. Since the model simulations in A and B represented cell cycle arrests, the attractors are the last states depicted on the X-axis.

Simulating the model with the constraints ’LT phenotype’ and ’HT phenotype’ (**S4 Table**) built from the transcriptome data of the strains at their highest supported ethanol level [24] showed cell cycle arrest for the HT and LT phenotypes under stress: the HT arrests in G1 phase due to the high activity of the SCF node, whereas the LT arrests in M phase (likely at anaphase) due to the lack of inhibition of the Clb1_2 node (**Fig 4**). Our *in vivo* analysis of cell growth showed that the wild-type LT SEY6210 strain displayed a higher population rebound after its highest ethanol stress relief than the wild-type HT BY4742 strain (**Fig 3C**).

### The effect of lnc9136 on cell cycle progression

All model simulations evaluating different expression levels (normal expression, downregulation and upregulation) of lnc9136 of the LT SEY6210 strain acting either as an activator or inhibitor without ethanol stress constraints returned a functional cell cycle.

**Fig 5:**
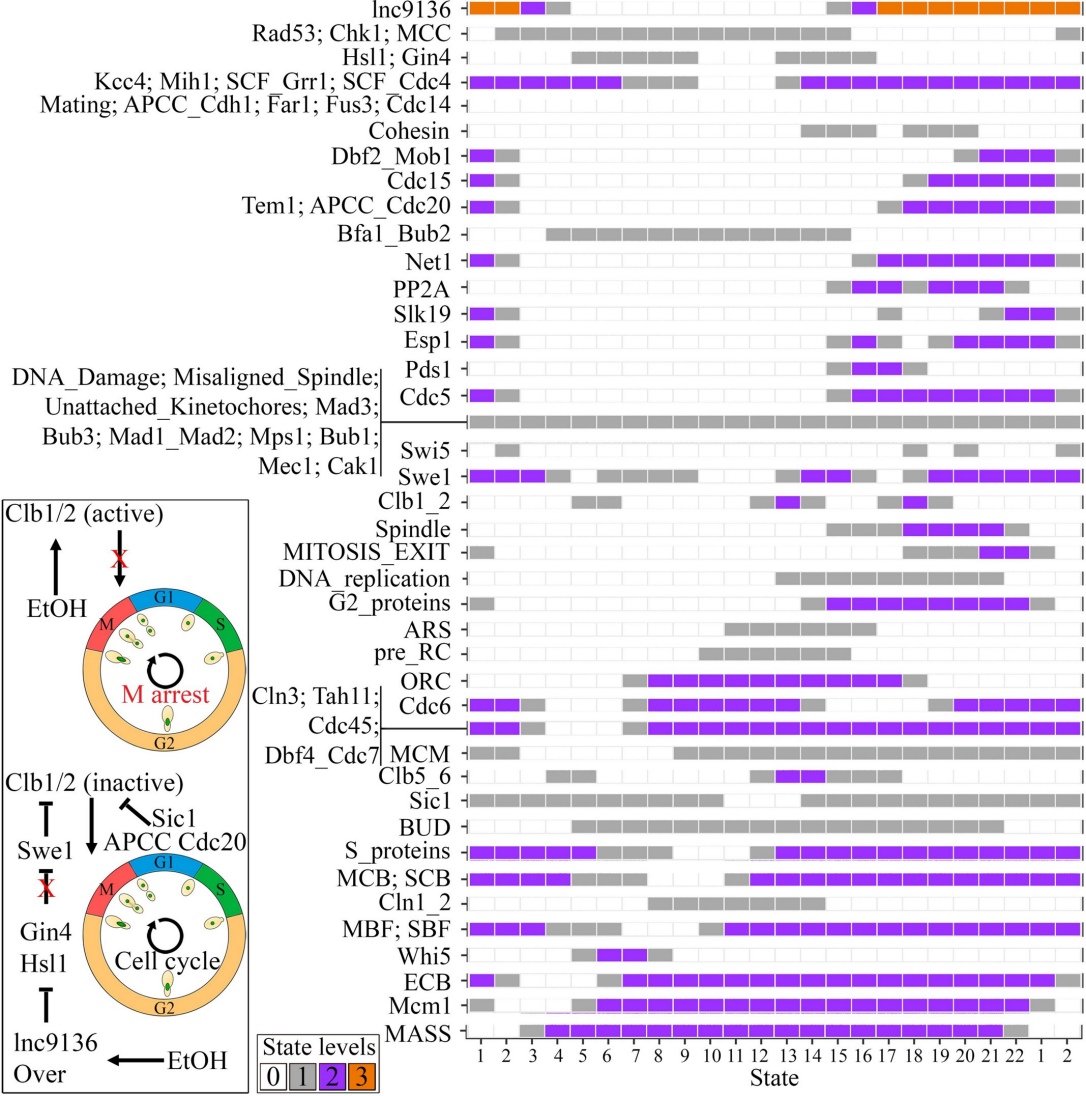
Simulating the SEY6210 cell cycle under ethanol stress with *in silico* overexpression of lnc9136 (the third experimental model simulation in Fig 1). The box color indicates the node levels in each simulation state. The simulation presented a cyclic attractor related to a functional cell cycle, which includes all states depicted on the X-axis. Thus, the functional cell cycle is evidenced when the simulation outcomes cyclic attractors presenting the activation of all phenomenological nodes, further inhibited when the MITOSIS_EXIT node reaches ’2’, and restarting the cell cycle (MASS returning to ’0’), as observed here (see details in the ’Model cycling rationale’ in the Materials and Methods). The upper picture in the box on the left is the LT cell cycle arrest mechanism reported in **Fig 4**, while the bottom picture reports our suggested M arrest skip mechanism that may occur in cells under ethanol stress. The red ’X’ depicts suppression of a given regulation. The red ’X’ on the edge head ended at the Swe1 node, indicates that this node was blocked neither by Gin4 nor Hsl1 when lnc9136 was overexpressed under ethanol (EtOH) stress.

LncRNAs may function as molecular decoys to inhibit target proteins [28]. Transcriptome analysis showed that lnc9136 was upregulated in strain SEY6210 under ethanol stress (log_2_ fold-change = 1.25) (NCBI BioProject number PRJNA727478). Our lncRNA-protein interaction prediction indicated that lnc9136 interacts with the G2 phase proteins Hsl1p and Gin4p (**Fig 2**). Interestingly, the *in silico* simulation in which lnc9136 was overexpressed in G2 phase and functioned as a target inhibitor in strain SEY6210 with ethanol stress constraints was the only condition that hindered arrest in M phase imposed by ethanol stress in the LT, returning a functional cell cycle. The APCC_Cdc20 node was updated to ’2’ in the last simulation states (**Fig 5**). According to these data, we suggest that lnc9136 may indirectly release Swe1p by inhibiting Hsl1p and Gin4p, and the joint actions among Swe1, Sic1, and APCC/Cdc20 inhibit Clb1/2 activity, resulting in mitosis exit in cells under ethanol stress (left box in **Fig 5**). Indeed, a similar simulation mentioned, but with inhibited APCC_Cdc20 node returned an M phase arrest.

Our *in vivo* analysis of cell growth of the SEY6210 lnc9136Δ1 mutant after stress relief showed that this mutant displayed a lower population rebound than the wild-type SEY6210 strain after most ethanol stress tests: the mutant grew better than the wild-type strain in the presence of 24% ethanol (volume/volume), and both genotypes had similar growth in the presence of 26% ethanol. However, only the differences in growth in 20% ethanol were statistically significant (**Fig 3D**). Additionally, the SEY6210 lnc9136Δ2 mutant was inviable (**S3 Fig**).

### The effect of lnc10883 on cell cycle progression

The *in silico* simulation overexpressing lnc10883 and acting as a target activator in HT BY4742 strain without ethanol stress constraint was the only case that returned an arrest in M phase; all other simulations examining this lncRNA without ethanol stress constraints returned a functional cell cycle.

Remarkably, all simulations that evaluated different expression levels (normal expression, downregulation and upregulation) of lnc10883 in BY4742 under ethanol stress (the third experimental model simulation) did not avoid the expected G1 phase arrest imposed by ethanol stress in HT. Therefore, we sought to determine the role of lnc10883 in the cell cycle.

**Fig 6:**
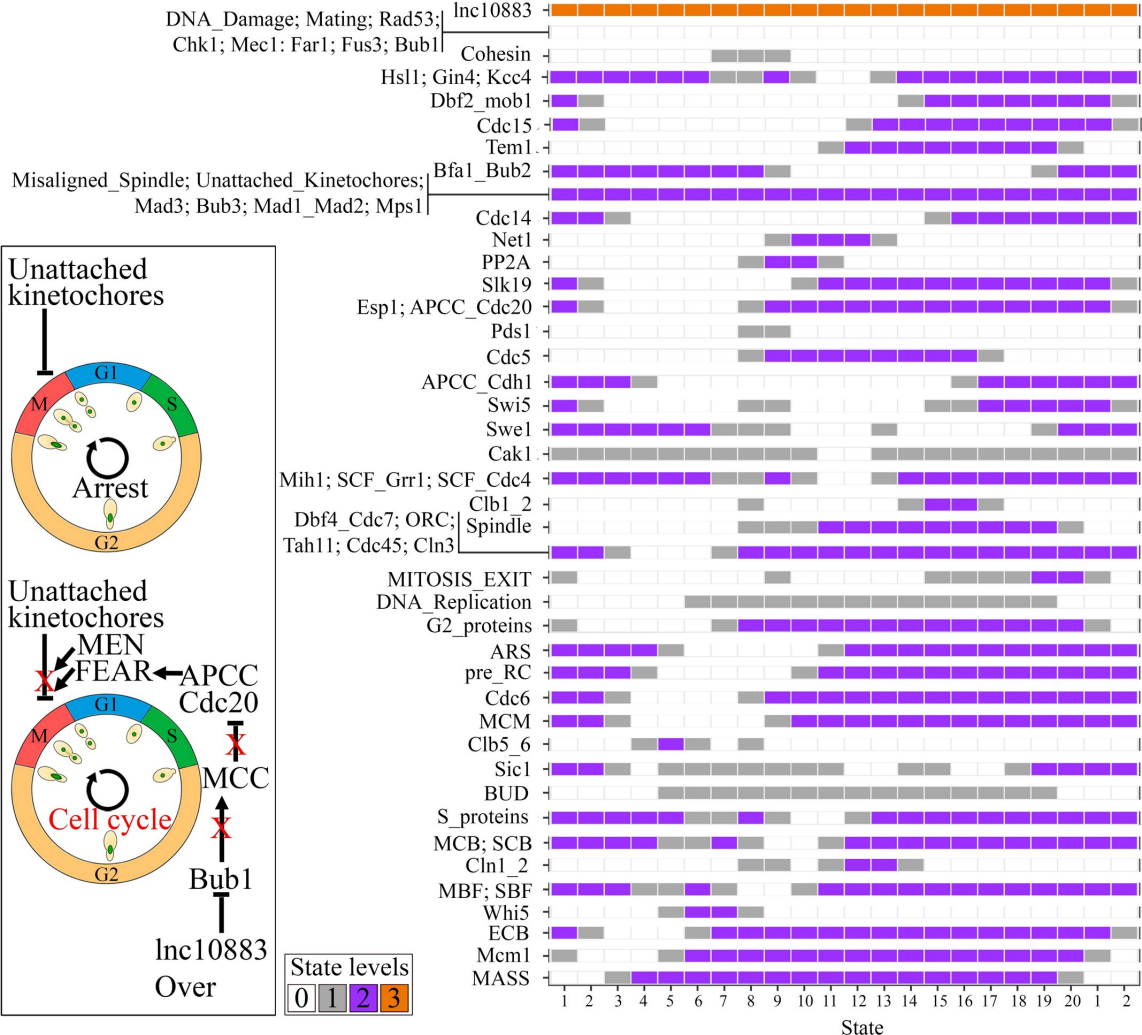
Simulating the BY4742 cell cycle with active spindle checkpoint arrest (Misaligned_Spindle and Unattached_Kinetochores nodes fixed at ’2’) to assess the role of lnc10883 overexpression (’Over’) *in silico* under these conditions (the fourth experimental model simulation in Fig 1). The box color indicates the node levels in each simulation state. The simulation presented a cyclic attractor related to a functional cell cycle, which includes all states depicted on the X-axis. Thus, the functional cell cycle is evidenced when the simulation outcomes cyclic attractors presenting the activation of all phenomenological nodes, further inhibited when the MITOSIS_EXIT node reaches ’2’, and restarting the cell cycle (MASS returning to ’0’), as observed here (see details in the ’Model cycling rationale’ in the Materials and Methods). The upper picture in the box on the left is the arrest mechanism mediated by spindle checkpoints, while the bottom picture reports our suggested arrest skip mechanism. The red ’X’ on the edges depicts the suppression of given regulations.

The transcriptome data showed that lnc10883 was upregulated in BY4742 cells under ethanol stress (log_2_ fold-change = 1.4) (NCBI BioProject number PRJNA727478). Interestingly, only simulating the lnc10883 overexpression to function as a target inhibitor in BY4742 cells with an active spindle checkpoint and without ethanol constraints precluded spindle checkpoint arrest. Based on these data, we suggest that in cells with an active spindle checkpoint, lnc10883 inhibits the Bub1p (we predicted that this lncRNA interacts with Bub1p), maintaining the MCC (mitotic checkpoint complex) inactive. Therefore, the activated APC/C complex (represented by the APCC_Cdc20 node in our model) triggers the FEAR pathway. The joint action of FEAR and MEN preclude spindle checkpoint arrest. Finally, the lack of Clb1/2 (the Clb1_2 node in our model) allows mitosis exit (the MITOSIS_EXIT node reaching ’2’ in our model), even likely with a damaged spindle (**Fig 6**).

### Simulating the effect of ethanol stress on the DNA damage pathways

Previous flow cytometry experiments showed that severe ethanol stress induces DNA damage in yeast. Furthermore, genes from this pathway are differentially expressed in all strains analyzed [24] (**Fig 3E**). Simulating the model with constraints from differentially expressed genes related to the DNA damage pathway (the second experimental model simulation in **Fig 1 and S4 Table**) showed that all LT strains (S288C, BY4741, and SEY6210) exhibited cell cycle arrest, indicating a functional checkpoint. Conversely, the HT strains X2180-1A and BY4742 did not present any arrest, suggesting cell cycle progression without DNA repair (the second experimental model simulation) (**S3 Fig and S4 Table**). The BMA64-1A strain did not present differential expression of the MEC1, CHK1, or RAD53 genes [24] (**Fig 3E**), preventing us from analyzing whether this strain underwent DNA damage-induced arrest.

Mec1p activates the Chk1 branch of the DNA damage checkpoint arrest (see the Discussion section). Since our prediction of the lncRNA-protein interaction showed that lnc10883 of BY4742 interacts with Mec1p, we decided to simulate whether this lncRNA functioned in avoiding DNA damage-induced arrest. The *in silico* simulation overexpressing lnc10883 acting as a target inhibitor in BY4742 with the same DNA damage constraint mentioned (the fourth experimental model simulations in **Fig 1**) showed that this lncRNA also skips DNA damage-induced arrest (**S3 Fig and S4 Table**). We suggest that lnc10883 acts on DNA damage checkpoint arrest by affecting the Chk1 branch in cells by interacting with Mec1p (further discussed), preventing the expected arrest from CHK1 overexpression (this gene is overexpressed in the BY4742 strain [24] (**Fig 3E**).

## Discussion

### Yeast cell cycle models

The published *S*. *cerevisiae* cell cycle models present different properties. For instance, simulation of a Boolean model that included all cyclins predicted a regular cell cycle, although this model contained few nodes [29]. The mentioned model [29] was further improved by including additional nodes and delays in the activation and degradation of nodes [30]. Furthermore, cell phenotypes were predicted by a probabilistic Boolean model [31]. Remarkably, a sizeable Boolean model with many interactions correctly predicted 62 of 85 mutant phenotypes tested [32].

Concerning models with multivalued nodes, a complex model with many cell cycle proteins presented good performance in emulating several cell cycle mutants described in the literature [26]. The number of nodes was expanded in a new model, although the model was validated only by simulating the G2/M phase transition [33]. A large model that included mRNAs, proteins, nutrients, and cellular events had nodes that could reach ten levels. Although this model facilitated complex simulations, it was validated by simulating only a single mutant [34].

Despite the progress in developing yeast cell cycle models, those mentioned above lack relevant properties for this study. For example: 1) crucial proteins (e.g., Gin4p, Hsl1p, and Bub1p) and lncRNAs were not considered; 2) pathway simplifications hinder the simulation of relevant characteristics for our purpose; 3) most models were developed to analyze specific mechanisms or subpaths; 4) many interactions and proteins were absent in previous models compared to the KEGG cell cycle pathway (sce04111) [27]; and 5) although Fauré’s model [26] presented reliable characteristics for our study, most of its nodes have up to two levels, preventing evaluations of low, normal, or high levels of expression, activity or molecule yields. Thus, we developed a model with multivalued nodes because these nodes increase the predictive accuracy of the model. These logic models address nonlinear behavior qualitatively, favoring the emulation of different abundances of RNA or proteins and the effects of differential expression [35–37].

Here, we used the model and concepts reported by Fauré et al. [26], the KEGG yeast cell cycle network [27], and many protein–protein and lncRNA-protein interactions to assemble a new logic model of the yeast cell cycle. Our model contains 67 nodes, 144 interactions, and broad node levels (up to 4 values), and the model was validated by predicting many experimental cell cycle mutants for each cell cycle phase. Finally, this new model was used to analyze the effect of ethanol on the cell cycle and the roles of stress-responsive lncRNAs in this process.

A simulation of systems perturbation (e.g., gene deletion) usually requires fixing the level of given nodes in the model along with the whole simulation [38] or toggling an off state in specific simulation points but also allowing some regulation [39]. To our knowledge, this study is the first to use transcriptomic data in equations of logic models to test the perturbation of yeast systems. In this case, our equations allow the downregulated nodes to reach a limited value range, whereas the upregulated nodes quickly update to the maximum value followed by regulations driven by other nodes.

### Evaluating the new cell cycle model

System robustness is evaluated by analyzing the stability of the basin of attractions of this perturbed system [40,41]. Model adjustment and validation by simulating cell cycle mutants ensure the reliability of the model [26]. Our model accurately recapitulated most cell cycle mutants generated in molecular biology experiments tested here, resulting in states that match the mutant phenotypes described in these papers. Our model is resilient to random perturbation, returning a functional cell cycle in most analyses, and the model also responds to the checkpoint nodes properly. In fact, the cell cycle is resilient and functions even under disturbances. For example, an *in vivo* analysis showed that only a low percentage of cell growth and division-related genes were deleted in viable mutants with slow growth [42]. Several computational models recapitulate the cell cycle robustness property [26,29–31,34,43,44]. Altogether, we suggest that our model is suitable for testing hypotheses concerning the cell cycle.

However, our model has three limitations. 1) DNA must be replicated only once per cycle to maintain cell stability [45], which is a mechanism that was not accounted in our model. 2) Although our model correctly predicted mutations related to the action of the FEAR and MEN pathways to control the release of Cdc14p, the Cdc14p release independent of the Cdc15p mechanism [46] is absent in our model. The insertion of Cdc14p independent release demanded reformulating bulk of interactions and equations, impairing other mechanisms that were previously well-modeled, and decreasing the number of correct predictions. 3) Our model did not specify spindle pole body duplication and cytokinesis.

### Late cell cycle arrest in LT strains induces a better rebound after ethanol stress relief

Experiments have reported that different stressors in yeast cause arrest in all cell cycle phases or even a time phase elongation [47–50]. Most cell growth in yeast occurs in M phase [51]. Interestingly, the fraction of cells in G1/S phases in populations after stress relief recovers faster than from interphase to M phase [50]. Our simulations suggest that LT and HT under ethanol stress were arrested in M and G1 phases, respectively. Altogether, we suggest that the LT strains may promptly shift to M phase exit after ethanol stress relief, while HT strains would still need to surpass G1 (and perhaps residual arrests at S/G2) and the time-consuming M phase growth. In fact, our *in vivo* analysis showed that the LT SEY6210 strain displayed a faster population rebound after stress relief than the HT BY4742 strain.

Our simulation showed that the G1 arrest observed in HT under ethanol stress may be due to the high activity of the SCF complex. Although *in vivo* inactivation or conditional expression of SKP1 (expressing an SCF complex protein) causes arrest in G1/G2 phase or inviability [52,53], to our knowledge, this study provides the first prediction regarding the response to SCF overactivity in yeast. Overall, we suggest that overactivity of the SCF-Cdc4 complex (represented by the SCF_Cdc4 node) may indirectly affect DNA replication, halting the cell cycle before S phase. Our simulation showed that the highest level of SCF_Cdc4 prevents Cdc6 node activation. Therefore, the lack of Cdc6 node activity precludes the activation of the pre-replication complex (represented by the pre_RC node), which is necessary for DNA_Replication node activation. Interestingly, *in vivo* experiments revealed that the SCF-Cdc4 protein complex drives the degradation of Cdc6p [54].

Furthermore, experiments report that Ndd1p synthesis depends on the MBF complex, which indirectly activates G2/M-specific genes [55]. Clb1/2 proteins are responsible for the G2/M transition [56]. Since Ndd1p was not modeled here, the activation of MCB by the MBF node is a relevant condition to activate the Clb1_2 node. Only three states had active Clb1_2 node in the HT under ethanol stress model constraints, which also seems to have contributed to the predicted G1 arrest.

The simulation showed that the continuous presence of the Clb1_2 node stalls LT in M phase. Likewise, experiments showed that the repression of Clb1/2 expression and their proteolysis mainly by the APC complex are important for mitotic exit [57–60]. Cells either with a nondegradable Clb2p form or lacking the APC complex have a high level of Clb2p throughout the cell cycle, precluding cell division [61].

### The lncRNA lnc9136 prevents cell cycle arrest in strain SEY6210 under ethanol stress

Lnc9136 is upregulated in SEY6210 cells under ethanol stress [24]. Simulating these conditions (lncRNA overexpression in SEY610 cells under ethanol stress) prevented the expected LT cell cycle arrest, resulting in a functional cell cycle. We hypothesize that defective lnc9136 hinders a functional cell cycle in the LT SEY6210 strain under stress. Thus, the *in vivo* mutant population would have a smaller size or a dearth of cells ready to divide than a wild-type population, hampering fast growth after stress relief. The *in vivo* partial deletion of two different regions of this lncRNA corroborated our hypothesis, since one mutation dampened the population rebound after stress relief, while the other mutant was inviable.

We suggest that the molecular mechanism in cells mediating the arrest release mentioned above relies on the lnc9136, Swe1, Sic1, and APCC/Cdc20. Overall, the overexpression of lnc9136 suppresses two Swe1 inhibitors (Hsl1p and Gin4p). Then, Swe1, Sic1, and APCC/Cdc20 inhibit the Clb1/2 proteins, leading to mitotic exit in SEY6210 cells under ethanol stress. Therefore, lnc9136 would exert a similar effect of HSL1 and GIN4 *in vivo* knockouts, increasing the abundance of Swe1p and cell viability [62]. Previous experimental evidence showed that the joint actions of Hsl1p and Gin4p to inactivate Swe1p drive septin formation, which in turn allows cells to enter M phase through a synergic effect with Clb1/2p activation [62,63]. Clb1/2p halts cells at the end of M phase. Therefore, the M phase exit relies on Clb1/2p inhibition [64]. Cdc20p (represented by APCC_Cdc20 in our model) is essential for APC/C [65] and suffices for mitotic exit in yeast by degrading Clb2p [66]. Additionally, the generated Cdc20Δ mutant is inviable [42] or arrested in late telophase [67]. Cdh1p functions in mitotic exit in cells [68]. Interestingly, the Cdh1 node was inactive in the SEY6210 arrest release simulation. However, inhibition of CDKs by Sic1 surpasses the observed cyclin proteolysis defects in Cdh1Δ, generating viable mutants [69]. Moreover, Sic1 inhibits Clb2 to regulate mitotic exit in cells [70].

LncRNAs act on cell cycle progression by indirectly regulating cyclins, CDKs, and transcription factors. For instance, the lncRNA GADD7 expressed in CHO-K1 cells interacts with Tar DNA binding protein 43, leading to Cdk6p mRNA degradation and preventing the transition from G1 to S phase [71]. LncRNAs in human cell lines may cause cell cycle arrest (usually through physical interactions with diverse proteins) [72–76] or promote cell proliferation [77–79].

Information on the effect of lncRNAs on the yeast cell cycle is still scarce, although these RNAs may be stress-responsive. For instance, Hog1 induces the transcription of the lncRNA Cdc28 under osmotic stress, promoting cell cycle reentry after stress relief [80]. Furthermore, four ncRNAs may be associated with ethanol stress [81].

### The lncRNA lnc10883 in BY4742 cells maintains cell cycle activity even in the presence of spindle and DNA damage

Severe ethanol stress induces DNA damage in the strains studied here [24]. Strikingly, our simulations showed that HTs completed an entire cell cycle even under DNA damage, whereas LTs presented the expected cell cycle arrest.

Two systems synergically control the DNA damage checkpoint: the Rad53p and Chk1p/Mec1p pathways [82]. However, activating a single pathway is sufficient to induce cell cycle arrest and repair DNA damage [82–84]. Although BY4742 cells overexpressed two DNA damage checkpoint genes in response to ethanol stress, the PDS1 (Chk1p pathway) and RAD53 genes were downregulated [24]. Therefore, we suggest that the DNA damage checkpoint might be inactive in this strain under ethanol stress. Interestingly, the simulation allows us to suggest that overexpression of lnc10883 in cells would inhibit Mec1p, inactivating the Chk1p pathway and hindering cell cycle arrest. Thus, we speculated that lnc10883 might be another hurdle to establish an arrest for proper DNA repair.

Here, we also suggested that the lnc10883-Bub1 complex inhibits MCC assembly in cells, a complex essential for inhibiting APC/C. Then, APC/C, MEN, and FEAR would release Cdc14p, causing further inhibition of Clb1/2 in anaphase and allowing cell division even in the presence of putative spindle damage. Indeed, the lack of Bub1p in mutants hampers an arrest in those cells treated with microtubule polymerization inhibitor nocodazole, maintaining the whole cell cycle even in cells with misaligned kinetochores [85].

Previous studies have shown the role of lncRNAs in cell cycle checkpoints. For instance, the suppression of the lncRNA PANDA in human fibroblasts sensitized them to apoptosis induced by DNA damage [86]. DNA damage-induced lncRNAs prompt the response to DNA damage in mouse cells [87]. Regulation of the DNA damage response mediated by lncRNAs is common in cancers, particularly by modulating the ATM, ATR, and p53 signaling pathways [88].

This paper presents a new yeast cell cycle logic model used to analyze the cell cycle dynamics during severe ethanol stress and the role of two ethanol stress-responsive lncRNAs. Our model was based on numerous classical genetic data, and the one was highly accurate in predicting cell cycle mutants described in the literature. Mutants generated here using the CRISPR–Cas9 approach also validated the prediction of one model, reinforcing the reliability of the model. Overall, simulations, cell culture experiments, and CRISPR–Cas9 mutants indicate that lncRNAs likely modulate the cell cycle under ethanol stress and the activation of DNA/spindle damage checkpoints by repressively binding to proteins involved in these pathways. A cell morphology analysis, DNA content measurement, and analysis using antibodies against proteins in each cell cycle phase are relevant approaches to test the reported findings. Finally, we claim that our cell cycle model is suitable for many studies focusing on cell cycle progression, an essential step for biotechnology development, tissue engineering, health science, and metabolic engineering.

## Materials and methods

### Network design, development of logical functions, and model evaluation

The yeast cell cycle was modeled in GINsim [89] using logical functions with Boolean operators AND, NOT, and OR. All simulations were performed using the synchronous update. The model usage is reported in the **S1 Video**. For the large-scale simulations, the command line GINsim was used (java -jar bioLQM.jar model.sbml -p <experimental perturbation> -r trace -u synchronous -i <initial state> -l 10).

A previously published model [26] was the scaffold network for our logic model. This scaffold was enriched with proteins and interactions from the KEGG yeast cell cycle pathway (sce04111) [27], demanding recasting logical functions. The concept of phenomenological nodes, the method to model mitotic exit, and the creation of logic functions were adapted from the previous model [26] (**Fig 1**). The nodes represent proteins, protein complexes, phenomenological nodes (MASS, BUD, DNA_Replication, Spindle, and MITOSIS_EXIT), checkpoint nodes (Mating, Unattached_Kinetochores, Misaligned_Spindle, and DNA_Damage), genomic regulatory elements (SCB, ECB, MCB, and ARS), and lncRNAs (lnc9136 and lnc10883), whereas interactions represent the activation or inhibition of nodes (**Fig 2**).

We redesigned the logical functions for most nodes to reach the values ’0’, ’1’, ’2’ or ’3’ (**Fig 1 and S1 Table**) because most of the nodes in Fauré’s model [26] only reached two levels. These logical functions rule the nodes’ value update throughout the simulation, as their update rules are satisfied at each step. Equations were adjusted until the model emulated a functional cell cycle of living cells: increasing mass, cell cycle activation, progression through all phases, mitosis, and decreasing mass (see a detailed description in the ’Model cycling rationale’). Based on data from the literature and the KEGG yeast cell cycle pathway (sce04111) [27], the level of each activator and the regulated nodes newly added here in the scaffold network was positively or negatively related to activation or inhibition, respectively (**Fig 1**). Nodes for which little information was available in the literature (e.g., Cak1 and Mih1) were limited to ’0’ or ’1’ to avoid spurious logical functions (**S1 Table**). The conversion of gene expression data for some selected genes into discrete logic equation values is described in detail in the “Simulating the effects of ethanol stress-responsive lncRNAs and ethanol on the cell cycle” section.

The formation of protein complexes such as MCC and the pre-replication complex (represented by the pre_RC node) was simplified here because logical models do not handle the mass flow. Therefore, the activation of a particular complex relies on the presence of all its components. The values of the nodes that represent protein complexes vary during the simulation as the smallest value of their components. As previously reported [26], complexes containing subunits without regulatory changes throughout the cell cycle were not included, and CDK Cdc28 functions were implicit in the Clb1_2, Cln5_6, Cln1_2 and Cln3 cyclin nodes.

The model and functions were readjusted and validated by simulating 109 published cell cycle mutant (knockouts and overexpression) phenotypes. These mutants were generated using classical genetic approaches (**S2 Table and Fig 1**). Our predictions for these mutants were compared with their phenotypes described in the literature to validate the model and assess reliability by calculating sensitivity and specificity. For this purpose, the simulations generating cyclic attractors with states corresponding to the expected cell cycle steps (G1, S, G2, M phase activation and mitotic exit) predicted viable mutants (described here as ’Viable’). Otherwise, the model predicted inviable mutants, which may have attractors corresponding to G1, S, G2, or M arrests (’Arrest in G1’, ’Arrest in S’, ’Arrest in G2’, or ’Arrest in M’, respectively) (the arrest definitions are described in detail in **Eq 1**). When the publication reports a particular mutant only as ’inviable’ (not specify the arrested phase) and our simulation converged to an attractor with states indicating an arrest, we considered our result a correct prediction. Thus, we labeled this mutant as ’Inviable’ before calculating the sensitivity and specificity. The model accuracy was improved by adding interactions and adjusting logic functions if a particular simulation did not match the published mutant phenotype.

The effect of checkpoint nodes (Unattached_Kinetochores, Misaligned_Spindle, Mating, and DNA_Damage) on cell cycle arrest was evaluated by simulating them with fixed activation values: 1) each checkpoint node was independently fixed at the minimum or maximum activated values and 2) all checkpoint nodes were fixed at the minimum or maximum activated value (**S3 Table**).

After validating the model, we tested its resilience to random system perturbations to certify its reliability. Since the cell cycle in living cells is a resilient system [42], a reliable model must withstand random cell cycle perturbations. One hundred, 1,000, and 10,000 independent simulations with random initial node values were performed to verify the reliability of the model (**Fig 1**); we expected a high frequency of predictions of a functional cell cycle even with systems perturbation. For this purpose, each node (except the checkpoint nodes) received a random initial value from ’0’ to ’3’ in each simulation by an *in-house* Python script. Checkpoint nodes (DNA_Damage, Misaligned_Spindle, Unattached_Kinetochores, and Mating) started with ’0’ values in all random cell cycle perturbation simulations to avoid putative spurious arrests. Therefore, the observed arrests were consequences of node regulation during simulation rather than pervasive outcomes.

The model reliability was analyzed again by performing an additional 229 simulations with random cell cycle perturbations ranging from G1 to mitotic exit phases (**Fig 1**). First, the system was set up to start the simulation in G1 phase (presenting only MASS, Cln3, Whi5, SBF, and MBF activation, and inactivation of the DNA_Replication node). An *in-house* Python script randomly selected a single node for each simulation to receive a random constraint to simulate the perturbations from G1 phase. In this case, the value of each selected node was fixed from ’0’ to ’3’ throughout the simulation.

### Model cycling rationale

Dynamic logical models reflect state transitions rather than quantities [26]. In this section, we describe how the simulation evolves to emulate a functional cell cycle and how the cell cycle arrests emerge.

The activation of each phenomenological node indicates that the simulation reached all conditions for the subsequent modeling steps. The phenomenological nodes (**Fig 2**) help to control and track the cell cycle progression, as previously described [26]: tracking the level of these nodes allows one to assess whether the model launched and completed mitosis or to assess the presence of cell cycle arrest (**S1 Fig) (**details are described below and in **Eq 1**).

In contrast to other nodes, the MASS is self-activated to ’1’, even after mitotic exit. The maximum MASS value (’2’) starts the cell cycle by activating the Mcm1 node: in living cells, Mcm1p regulates the expression of G1 and S genes [90]. Then, Clns nodes induce the activation of the SBF, MBF, and BUD nodes. Next, Cdc45, Tah11, Cdc6, MCM, and ORC nodes activate the pre_RC node. The latter and Clb5_6 nodes jointly activate the DNA_Replication node, which represents both the replication process and the presence of duplicated DNA, an active state occurring until mitotic exit. The DNA_Replication node triggers the G2/M transition by activating the G2_proteins node: the latter starts several nodes related to M phase at the appropriate states. The activation of the MITOSIS_EXIT phenomenological node (’1’) starts M phase and requires activation of the phenomenological nodes (except MASS) and the Clb1_2 node. The phenomenological nodes (MASS, BUD, DNA_Replication, and Spindle) maintain the maximum value until suppression by the MITOSIS_EXIT node. Then, the mitotic exit in a functional cell cycle depends on Clb1_2 node inhibition, inducing MITOSIS_EXIT node to reach ’2’. Thus, all phenomenological nodes, including MASS, are updated to ’0’ to restart the cycle. Overall, the simulation outcomes revealed a functional cell cycle when the state transition graph has a cyclic attractor containing the activation of each phenomenological node followed by the peak MITOSIS_EXIT value. Then, all phenomenological nodes are suppressed, restarting the cell cycle. Finally, the MASS is updated again to ’1’ while MITOSIS_EXIT < ’2’.

The model simulations that output a state transition graph with a MASS different than ’0’, while MITOSIS_EXIT < ’2’ indicated cell cycle arrest. Tracking the state of the DNA_replication, S_proteins, and G2_proteins nodes allow us to seek the phase in which the cell cycle was arrested (**Eq 1**).

$$\left\{\begin{array}{c} L=\langle 0,1,2,3\rangle \\ EF⇐(E\wedge (\sum_{i=l_{1}}^{i=l_{4}}P_{i}=0)\wedge (\mathrm{MITOSIS}\_\mathrm{EXIT}=l_{3}))|P=\{\mathrm{MASS},\mathrm{BUD},\mathrm{DNA}\_\mathrm{Replication},\mathrm{Spindle}\}\\ A⇐(E\wedge (MASS>l_{1})\wedge (\mathrm{MITOSIS}\_\mathrm{EXIT}<l_{3}))\\ G1\;\mathrm{arrest}⇐(A\wedge (\mathrm{DNA}\_\mathrm{Replication}=l_{1}))\\ S\;arrest⇐(A\wedge (DNA\_\mathrm{Replication}>l_{1})\wedge (S\_\mathrm{proteins}=l_{1}))\\ G2\;\mathrm{arrest}⇐(A\wedge (\mathrm{DNA}\;\mathrm{Replication}>l_{1})\wedge (G2\_\mathrm{proteins}>l_{1}))\\ M\;\mathrm{arrest}⇐(A\wedge (\mathrm{MITOSIS}\_\mathrm{EXIT}=l_{2})) \end{array}\right\}$$ Eq 1

where *L* is the possible nodes values and *l*_*n*_ is the *l*_th_ element of *L*, e.g., *l*_*1*_ = 0, whereas *l*_*2*_ = 1. *EF* is the end of a simulation of a functional cell cycle, *E* represents the end of a simulation by reaching a basin of attraction, *P* indicates the set of phenomenological nodes, and *A* indicates arrest.

The value of checkpoint nodes drives the value update of checkpoint-influenced nodes (Mec1, Chk1, Rad53, Bub1, Mps1, Bub3, Mad3, Mad1_Mad2, MCC, Bfa1_Bub2, Far1 and Fus3), which may peak at ’1’ or ’2’. For instance, the Mec1 node peaks at the value ’2’ when its related checkpoint node (the DNA_Damage) peaks at ’2’, whereas Far1 and Fus3 and their related checkpoint node (Mating) will jointly peak at ’1’.

Checkpoint nodes reaching ’2’ usually force cell cycle arrest by inhibiting crucial nodes for cell cycle progression (**S3 Table**). However, negative feedback from the phenomenological node Spindle may deactivate checkpoint nodes below ’2’. Since Mating is not essential for our goals, we set it as a Boolean node: its activation causes an arrest in G1 phase.

During simulations, the node values updating from ’0’ to ’2’ rely on the intermediary transition from ’0’ to ’1’. Thus, nodes with equations lacking rules to drive an update to ’1’ (e.g., Whi5 and G2_protein nodes) may also pass by the value ’1’. GINsim [89] automatically updates the values of nodes that did not reach ’1’, ’2’, or ’3’ to ’0’.

Altogether, the preset to simulate a regular cell cycle (without any perturbation) was the initial state of MASS and checkpoint node (except Mating) levels at ’1’, whereas the other nodes must start at ’0’.

### Simulating the effects of ethanol stress-responsive lncRNAs and ethanol on the cell cycle

Four experimental model simulations were performed to study the cell cycle under ethanol stress and the role of lncRNAs. The general presets of inputs for these simulations were the same as those used to emulate a regular cell cycle, in addition to the specific requirements for each experiment. The first analysis evaluated the effect of ethanol on the HT and LT phenotypes. The second experiment assessed the effect of ethanol on the DNA damage checkpoint. The purpose of the third analysis was to assess whether the lncRNAs lnc9136 and lnc10883 prevented cell cycle arrest induced by ethanol stress. The goal of the fourth experimental model simulation was to study the role of lnc10883 in a regular cell cycle but with DNA damage or spindle checkpoint active (**Fig 1**).

A strong correlation between RNA and protein levels has been observed in yeasts [91]. Hence, we assumed the protein yield in our simulations based on the transcriptome data to simulate the effect of ethanol stress on the cell cycle and DNA damage checkpoint activation (**Fig 1**).

We previously measured the highest ethanol tolerance level of six yeast strains. Each strain was treated with YPD medium (2% peptone, 1% yeast extract, and 2% glucose) with different ethanol concentrations: the highest tolerated ethanol level of a particular strain was the highest ethanol concentration (volume/volume) that allowed its surveillance and growth on YPD plates. Thus, BMA64-1A (tolerates 30% ethanol), BY4742 (26%), and X2180-1A (24%) were classified as displaying the HT phenotype, whereas BY4741 (22%), SEY6210 (20%), and S288C (20%) were classified as displaying the LT phenotype. Then, we obtained the transcriptome data from these strains under the treatment (the highest ethanol level supported for each strain) and control conditions (NCBI BioProject number PRJNA727478). We used the transcriptome to assess differential expression and calculate the log2 fold-change between treatment and control conditions (using DESeq2 with default parameters [92]). We classified each gene based on the log2 fold-change as:

downregulated<non‐differentiallyexpressed<upregulated,

where the ’non-differentially expressed’ genes had a log2 fold-change = 0, and the down- and upregulated differentially expressed genes must have a false discovery rate < 0.01 [24].

Cell cycle-related genes with similar up- or downregulated expression profiles in all three strains within each phenotype were selected to simulate the effect of ethanol on the yeast cell cycle (**Fig 1**). The expression levels of these 10 selected genes (**S5 Table**) were converted to logical functions as follows: 1) nodes corresponding to the downregulated genes may change between ’0’ and ’1’ during the simulation, indicating the absence or a low protein yield, respectively; 2) nodes corresponding to the upregulated genes reach ’3’ (high protein yield) soon after activation, which may be either sustained or reduced according to regulation by other nodes during simulations; and 3) nodes related to the non-differentially expressed genes vary from ’0’ to ’2’ according to their regulations. For instance, the Sic1 gene is not differentially expressed in HT strains, whereas it is downregulated in LT strains. Therefore, Sic1 is updated to ’0’ or ’1’ to simulate the effect of ethanol on LT strains and from ’0’ to ’2’ in HT strains. Simulations testing the cell cycle of HT and LT phenotypes under ethanol stress were performed separately using the ’HT phenotype’ and ’LT phenotype’ model constraints, respectively (the first experimental model simulation). The same procedures were performed to evaluate the effect of ethanol on DNA damage checkpoints in each strain in independent simulations (the second and part of the fourth experimental model simulations) (**S4 Table**).

The genomic coordinates of SEY6210 (an LT strain) and BY4742 (an HT strain) lncRNAs [24] are deposited at https://figshare.com/articles/dataset/LncRNAs_annotations/17086109, and the sequences are in the NCBI accession numbers MZ099632 and MZ099633 and **S1 Data**. LncRNA-protein interactions in SEY6210 and BY4742 were predicted using lncPRO [93] (default parameters). The probability of interactions exhibited a Poisson distribution. Therefore, only interactions with a probability ≥ 0.95 were selected. We sought ethanol stress-responsive lncRNAs that interacted with the cell cycle proteins depicted in **Fig 2**. Thus, the interactions between lnc9136 and Gin4p and Hsl1p in SEY6210 and between lnc10883 and Mec1p and Bub1p in BY4742 were included in the network (**Figs 1 and 2**) before the third and the fourth experimental model simulations.

The third experimental model simulation was performed to assess whether the lncRNAs precluded the cell cycle arrests imposed by ethanol stress reported in the first experimental model simulation (**Fig 1**). Thus, the same ’LT phenotype’ and ’HT phenotype’ model constraints used in the first experimental model simulation (**S4 Table**) were applied to emulate the effects of lnc9136 on the LT SEY6210 and lnc10883 on the HT BY4742 under ethanol stress. No experimental evidence is available regarding the effects of these lncRNAs. Thus, lnc9136 and lnc10883 were simulated as activators or inhibitors under normal expression, down- and upregulation. The normal, down- and upregulation expressions and activity profiles (activators or inhibitors) mentioned for these lncRNAs were applied in the regular cell cycle setting (without ethanol stress constraints) followed by simulations to investigate how these lncRNAs function in the cell cycle of cells not exposed to any stressor.

Initial assays toggling a constitutive overexpression of lnc9136 on the SEY6210 with ethanol stress model constraints revealed no release of cell cycle arrest. Hsl1p and Gin4p (proteins that bind to lnc9136) function in G2 phase (KEGG pathway sce04111; SGD database). Then, we investigated the effect of lnc9136 on cell cycle arrest by simulating its overexpression starting in G2 phase through regulation by the G2_proteins node: lnc9136 reaches a value of ’3’ soon after G2_proteins node activation.

The effect of lnc10883 on the spindle and DNA damage pathways in BY4742 (the fourth experimental model simulation) was examined based on the hypothesis that strains stressed with ethanol exhibit spindle and DNA damage (**Fig 1**). Thus, we independently simulated the upregulation of lnc10883 acting as a target inhibitor or activator in the regular cell cycle presets (without ethanol stress constraints) but maintaining the spindle checkpoint active (Misaligned_Spindle and Unattached_Kinetochores nodes fixed at the maximum level (’2’)) or with model constraints from the expression of DNA damage-related genes (’BY4742 DNA damage’ constraint in **S4 Table**).

### Partial deletion of lncRNA 9136

To evaluate the modeled hypothesis regarding the influence of lncRNA lnc9136 on cell cycle arrest release in SEY6210 under severe ethanol stress, we generated partial deletion mutants (lnc9136Δ1, and lnc9136Δ2) of this lncRNA using CRISPR-Cas9 (**Table 2**) followed by population rebound experiments (see the “Population rebound experiments”section).

**Table 2:** Oligonucleotides used in this study. Bold nucleotides indicate the genomic target sequence. **“Δ1”**: oligonucleotides used to obtain the SEY6210 lnc9136Δ1 mutant; **“Δ2”**: oligonucleotides used to obtain the SEY6210 lnc9136Δ2; *: phosphorylated primer end. Details of target-homology repairs and Cas9 cleavage loci are shown in **S4 Fig**.

Oligos	Sequence
^Δ1^F pMEL16	*5’ **TCATGTACTCCATAGAGTGA**GTTTTAGAGCTAGAAATAGC 3’
^Δ1^F repair.	5’ TCCACACACCCATTTCGCTAGCGTAGAACAAGGGGAGACACAAACTTTCTTTTCCTTGCATAATTATTTCCCTCGTTGCTACTCATTGAGGCCGCTCCATATGGAGATTTGAAAAAGGTT 3’
^Δ1^R repair	5’ AACCTTTTTCAAATCTCCATATGGAGCGGCCTCAATGAGTAGCAACGAGGGAAATAATTATGCAAGGAAAAGAAAGTTTGTGTCTCCCCTTGTTCTACGCTAGCGAAATGGGTGTGTGGA 3’
^Δ2^F pMEL16	*5’ **AAGCCTTGCCTAAAAAAAGTG**GTTTTAGAGCTAGAAATAGC
^Δ2^F repair	5’ TCCTGCACTGAATTTACACAGAAGTTAAGAACCGCCTCTGCTTTTCTGGTATTATTTTGCATCTCAAAATATAGGCAATTACCAGGTATACGATATTTCCTCAAAATGAAAATGCCTAAA 3’
^Δ2^R repair	5’ TTTAGGCATTTTCATTTTGAGGAAATATCGTATACCTGGTAATTGCCTATATTTTGAGATGCAAAATAATACCAGAAAAGCAGAGGCGGTTCTTAACTTCTGTGTAAATTCAGTGCAGGA 3’
R pMEL16	*5’ GATCATTTATCTTTCACTGC 3
F M13	5’ TGTAAAACGACGGCCAGT 3’
R M13	5’ CAGGAAACAGCTATGAC 3’

The plasmids pMEL16 (His^-^, Addgene 107922) and p414-TEF1p-Cas9-CYC1t (hereafter referred to as P414, Addgene 43802) were used to express gRNA and Cas9, respectively. P414 has the KAN selective marker instead of TRP1 (donation from Dr. Arnold Driessen of the University of Groningen, The Netherlands).

The target regions were inserted into pMEL16 by PCR: 20 ng of pMEL16 plasmids, 1 μL of Phusion (NEB M0530S), 1X of Phusion Buffer, 0.1 mM of each dNTP, 0.4 μM of F pMEL16 and, 0.4 μM of R pMEL16 in a final volume of 25 μL (**Table 2**). The touchdown PCR reaction was 98°C for 1 min, followed by 5 cycles with a high Tm (98°C for 30 sec, X°C for 30 sec, and 72°C for 6 min), 10 cycles with mid Tm (98°C for 30 sec, Y°C for 30 sec, and 72°C for 6 min), 20 cycles with low Tm (98°C for 30 sec, Z°C for 30 sec, and 72°C for 6 min), and 72°C for 6 min. The Tm X°C, Y°C and Z°C are the averages between the melting temperature of R pMEL16 and the F pMEL16 of each gene, plus 9°C, 5°C and 2°C, respectively. Then, 25 μL of amplicons were digested with 1 μL of DpnI (NEB R0176S). 100–200 ng of purified digestion was ligated by T4 DNA ligase (Promega M1801), and 2 μL of products were transferred into 40 μL of TOPO competent cells (ice incubation for 20 min, 42°C for 50 sec, and heat shock on ice for 2 min). Cells were incubated in 200 μL of LB medium at 37°C for 1h, followed by overnight incubation (37°C) in LB plates with 0.05 mg/mL of ampicillin. Colonies with modified pMEL16 were sought by PCR-RFLP using M13 oligos (**Table 2**) and ClaI digestion (Bsu16l, Thermo Fisher IVGN0306) (37°C for 1h). Positive colonies were grown in liquid LB with 0.05 mg/mL of ampicillin, and plasmids were extracted using the QuickLyse Miniprep system (Qiagen 27405).

Yeast cells were grown overnight (30°C) in liquid YPD (2% peptone, 1% yeast extract, and 2% glucose), diluted in the same medium to an OD_600_ of 0.3, and incubated (30°C, and 200 RPM) until reach an OD_600_ of 1.0. Competent cells were obtained using the Yeast Transformation Kit (Sigma YEAST-1KT).

A solution with 10 μL of salmon testes DNA, 600 μL of plate buffer (both from the Sigma YEAST-1KT kit), 1 μg of the P414 plasmid, 1 μg of modified and purified pMEL16 plasmid, 5 μL of double-strand repair DNA (**Table 2**), and 100 μL of competent yeast cells were incubated at 30°C for 30 min, and 10% of DMSO was further added. The samples were immediately incubated at 42°C for 15 min, quickly transferred into ice, and chilled for 2 min. Double-strand repair DNA was obtained by mixing 100 μM of F and R repair oligos for each target locus (**Table 2**), followed by incubation (95°C for 10 min) and slow cooling on the bench.

Cells were harvested by centrifugation (2,000 RPM for 30 sec), and the supernatant was discarded by careful pipetting. The pellet was diluted in 250 μL of drop-out medium His^-^ (Yeast Synthetic Drop-out Medium Supplement without Histidine, Sigma Y1751) with an initial concentration of 1.92 mg/mL, supplemented with 20% of glucose, and 1.9 mg/mL of Yeast nitrogen base without amino acids and ammonium sulfate. Tubes were incubated (30°C, 200 RPM for 2h) and plated on drop-out medium His^-^ (the same one mentioned, plus 2% of bacto agar, and 0.2 mg/mL of G418) plates. Plates were incubated at 30°C until colonies emerged. Mutants were screened by standard colony PCR and sequenced using the Sanger method. Mutants were grown in liquid YPD medium with 0.2 mg/mL of G418. Finally, 700 μL of mutant cells plus 15% glycerol were stored at -80°C until use.

### Population rebound experiments

We investigated the population rebound of the SEY6210 lnc9136Δ1 mutant and the wild-type SEY6210 strain after the high ethanol stress relief. Overnight cells grown in YPD (YPD plus 0.2 mg/mL of G418 for mutant strains) at 30°C were further diluted in YPD to an OD_600_ of 0.3 and incubated at 30°C, and 200 RPM. Then, ~400 μL of cells (OD_600_ of 0.2) were harvested by centrifugation (2,000 RPM for 2 min), and the medium was carefully discarded by pipetting. Pelleted cells were diluted in 2 mL of preprepared YPD medium with different ethanol concentration (18%, 20%, 22%, 24%, and 26% (volume/volume)). The content was transferred into 10 mL rounded bottom tubes and immediately incubated (30°C, 135 RPM for 1h). Then 1 mL of cells were harvested by centrifugation (2,000 RPM for 2 min), the medium was carefully discarded by pipetting, and the pellet was diluted in 1 mL of YPD medium. Finally, we transferred 200 μL of sample for each well-plate to obtain four technical replicates. Plates were incubated at 30°C for 24 h, and OD_600_ was measured each five min after 30 sec of orbital shaking. The same protocol was used to compare the rebound of BY4742 and SEY6210 wild-types after treatment with 26% and 20% of ethanol (their highest ethanol tolerance levels [24]), respectively. We compared the Log *K* (growth rate) between SEY6210 wild-type vs. SEY6210 lnc9136Δ1 mutant and between SEY6210 vs. BY4742 wild-types using the mixed-effects model (Geisser-Greenhouse correction, Sídák test, and swap direct comparisons), and the Mann-Whitney U (unpaired mode, non-parametric, and two-tailed) tests, respectively.

## Supporting information

S1 Fig State transition graph of the yeast cell cycle without perturbation. The box color indicates the node values in each state. The ’X’ axis contains all the states of the attractor. The functional cell cycle is observed when the simulation results in cyclic attractors presenting the activation of all phenomenological nodes, further inhibited when the MITOSIS_EXIT node reaches ’2’ and restarting the cell cycle (MASS returning to ’0’), as observed here. The boxes on the left side show the key events along the systems evolution. The numbers on the left side of phenomenological nodes represent the order of node activation to emulate a functional cell cycle.
(PDF)

S2 Fig Lethality tests. The heading lists the combination of vectors and the DNA repair tested, while the first columns indicate the medium used. The YPD plates showed that all experiments allowed cell surveillance in a rich medium. The plates with G418 containing or lacking P414 evidenced that this vector is expressed. A similar conclusion was obtained for the pMEL16 in cells plated or not plated on drop-out (DO) His^-^ medium. Based on the expected plate profiles of all experiments observed and the profile of cells harboring the two vectors + repair DNA plated onto the DO His^-^ + G418 plate (the box dashed figure), we conclude that both vectors are properly working (expressing Cas9 and His marker) and that the presence of repair DNA is responsible for inducing lethality by conducting the deletion properly.
(PDF)

S3 Fig Simulations of the DNA damage checkpoint for each strain (the second experimental model simulation) and the role of the lnc10883 in BY4742 in the DNA damage checkpoint (the fourth experimental model simulation). The simulations were performed using only the node MASS active (level ’1’) as the initial state plus the model constraints created from the expression of DNA damage-related genes (**S4 Table and Fig 3E**). Notably, lnc10883 was fixed at ’3’ for the *in silico* overexpression simulation. All simulations related to the HT strains returned a functional cell cycle. Thus, the X-axis represents all states corresponding to the cyclic attractor. Conversely, simulations related to LT strains returned an arrest, as depicted by a steady state with the single-state attractors reported in the last state on the X axis.
(PDF)

S4 Fig Design of DNA repair for partial deletions of lnc9136 (A and B) and representation of the Cas9 cut site at the genomic target (the yellow and orange boxes).
(PDF)

S1 Table Details of the logical equations for the yeast cell cycle nodes. The references used to design the logic equations are cited in parentheses after each node name. The notation in the equations was designed according to the symbols and rules demanded by the GINsim manual. We highlight details concerning some complex interactions: A) Whi5 is not able to completely inhibit MBF and SBF, even when overexpressed. Then, Whi5 does not arrest the cell cycle, causing only a slight reduction in cell size; B) Inhibition of Clb1_2 relies on the action of the APC complex leading to mitotic exit, which is not performed by Sic1 alone. Thus, at least one part of the APC complex must be active (represented in our model by APCC_Cdc20 or APCC_Cdh1 nodes) to ensure that the model functions for several mutants tested here; C) The Cak1 null mutant is inviable, and information about this gene is scarce. Therefore, Cak1 was modeled here as a Boolean node essential for Clb1_2 activation; D) We stated that Hsl1 and Cdc5 must be active to simulate complete inhibition of Swe1, according to a previous report. However, due to the lack of information in the literature, Kcc4 and Gin4 were modeled as nodes able to reduce Swe1 levels but without inducing complete inhibition; E) CDKs were implicitly present in some nodes, as previously published (Cdc28 is implicit in Clb1_2, Cln5_6, Cln1_2, and Cln3 nodes); F) The Mcm2 protein is implicit in the MCM node; G) ! MASS refers to a MASS = 0 level; H) “*” indicates nodes responsible for activating other nodes usually expressed in these phases; I) Node types (TF, transcription factor; PKC, protein/kinase complex; KI, kinase inhibitor; TR, transcriptional repressor; DRIF, DNA replication initiation factor; DRLF, DNA replication licensing factor; ABP, ATP-binding protein; UPL, ubiquitin-protein ligase; K, kinase; CDKI, cyclin-dependent kinase inhibitor; CDKK, cyclin-dependent kinase-activating kinase; CSCC, component of the spindle-assembly checkpoint complex; KS, kinase substrate; KAP, kinetochore-associated protein; PP, protein phosphatase; CSRC, core subunit of the RENT complex; GAP, GTPase-activating protein; GTP, GTPase; RgEl, regulatory element; PCo, protein complex).
(PDF)

S2 Table List of mutations used for model adjustment and validation. The procedure used to simulate each mutant is presented in the column ’GINsim input’.
(PDF)

S3 Table Exploring the effect of checkpoint nodes on the cell cycle model. **Note A:** General setting used to test mutations, random cell cycle perturbations, and the effects of ethanol and lncRNAs on the cell cycle. **Note B:** Setup of checkpoint nodes fixed at the minimum activated value. **Note C:** The MATING node had only the activation value ’1’, which is its maximum or minimum activated value. **Note D:** Setup for each checkpoint node fixed at the maximum activated value. **Note E:** Setup of all checkpoint nodes fixed at the minimum activated value. **Note F:** Setup of all checkpoint nodes fixed at the maximum activated value.
(PDF)

S4 Table Model constraints used to simulate the effects of ethanol on the cell cycle (‘*’, the first experimental model simulation), the DNA damage pathways (the second experimental model simulation), and the effect of lncRNAs on the cell cycle based on the transcriptome data (the third and fourth experimental model simulations). The numbers in brackets represent the up- or downregulation profiles for each phenotype from the data reported in **S5 Table and Fig 3E** in the main text. According to the following definition *downregulated<non-differentially expressed<upregulated*, where the ’non-differentially expressed’ genes had a log2 fold-change = 0 and the differentially expressed genes must have a false discovery rate < 0.01, the nodes related to the downregulated genes were model-constrained to a value of ’0’ or ’1’, whereas the nodes related to the upregulated genes reached a value of ’3’. For instance, Rad53[0,1] constrains the node Rad53 to assume only values of ’0’ or ’1’ during simulations. The notation X[Y@n] indicates that the logical functions of the X node will operate such as the Y = ‘n’ level (the X’s node regulator). For instance, Chk1[Mec1@2] will drive the Chk1 node updates assuming Mec1 = 2, although Mec1 will be updated according to its logic equations. This approach enables to peak the maximum level for these nodes but not excluding the possibility to evolve during the simulation. The notation in the equations was designed according to the symbols and rules indicated in the GINsim manual.
(PDF)

S5 Table Log2 fold-changes in the differentially expressed genes related to the cell cycle used here. The “-” symbol indicates the lack of differential expression (the non-differentially expressed genes).
(PDF)

S1 Data Sequences of lncRNAs studied here in FASTA format. Name; strain: genomic coordinates start-end; orientation.
(PDF)

S1 Video Usage of the yeast cell cycle model in GINsim (http://ginsim.org/). (ZIP)
