# Supplementary material for: LncRNAs of Saccharomyces cerevisiae bypass the cell cycle arrest imposed by ethanol stress
Source: PLoS Comput Biol. 2022 May 19;18(5):e1010081. doi: 10.1371/journal.pcbi.1010081 (PMC9232138; doi:10.1371/journal.pcbi.1010081)

S1 Fig: State transition graph of the yeast cell cycle without perturbation. The box color indicates the node values in each state. The 'X' axis contains all the states of the attractor. The functional cell cycle is observed when the simulation results in cyclic attractors presenting the activation of all phenomenological nodes, further inhibited when the MITOSIS_EXIT node reaches '2' and restarting the cell cycle (MASS returning to '0'), as observed here. The boxes on the left side show the key events along the systems evolution. The numbers on the left side of phenomenological nodes represent the order of node activation to emulate a functional cell cycle.

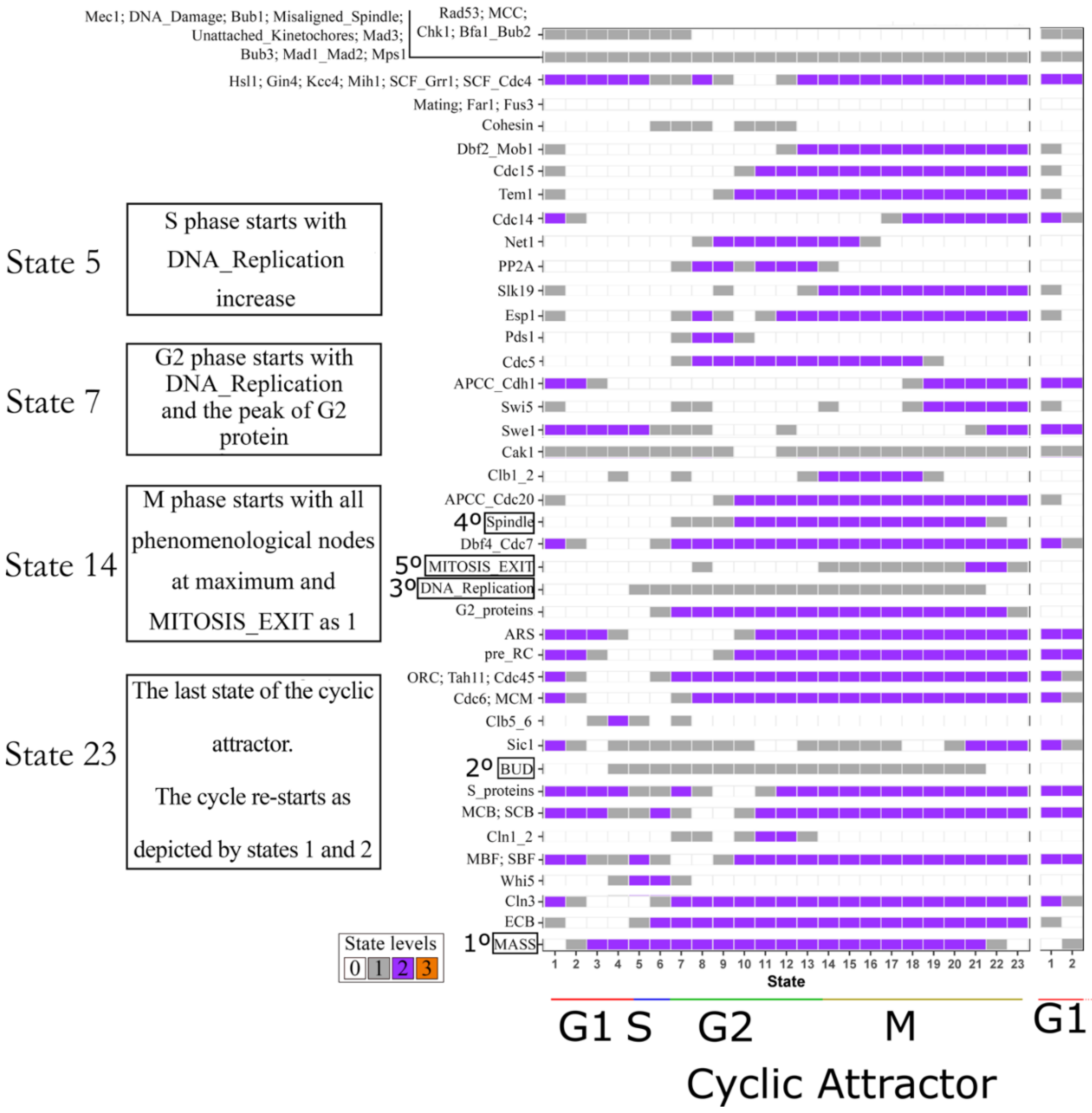

Supplement: S1 Fig — The box color indicates the node values in each state. The ’X’ axis contains all the states of the attractor. The functional cell cycle is observed when the simulation results in cyclic attractors presenting the activation of all phenomenological nodes, further inhibited when the MITOSIS_EXIT node reaches ’2’ and restarting the cell cycle (MASS returning to ’0’), as observed here. The boxes on the left side show the key events along the systems evolution. The numbers on the left side of phenomenological nodes represent the order of node activation to emulate a functional cell cycle. (PDF) [file pcbi.1010081.s001.pdf]
