# Supplementary material for: LncRNAs of Saccharomyces cerevisiae bypass the cell cycle arrest imposed by ethanol stress
Source: PLoS Comput Biol. 2022 May 19;18(5):e1010081. doi: 10.1371/journal.pcbi.1010081 (PMC9232138; doi:10.1371/journal.pcbi.1010081)
Supplement: S2 Fig — The heading lists the combination of vectors and the DNA repair tested, while the first columns indicate the medium used. The YPD plates showed that all experiments allowed cell surveillance in a rich medium. The plates with G418 containing or lacking P414 evidenced that this vector is expressed. A similar conclusion was obtained for the pMEL16 in cells plated or not plated on drop-out (DO) His- medium. Based on the expected plate profiles of all experiments observed and the profile of cells harboring the two vectors + repair DNA plated onto the DO His- + G418 plate (the box dashed figure), we conclude that both vectors are properly working (expressing Cas9 and His marker) and that the presence of repair DNA is responsible for inducing lethality by conducting the deletion properly. (PDF) [file pcbi.1010081.s002.pdf]

S2 Fig: Lethality tests. The heading lists the combination of vectors and the DNA repair tested, while the first columns indicate the medium used. The YPD plates showed that all experiments allowed cell surveillance in a rich medium. The plates with G418 containing or lacking P414 evidenced that this vector is expressed. A similar conclusion was obtained for the pMEL16 in cells plated or not plated on drop-out (DO) His^- medium. Based on the expected plate profiles of all experiments observed and the profile of cells harboring the two vectors + repair DNA plated onto the DO His^- + G418 plate (the box dashed figure), we conclude that both vectors are properly working (expressing Cas9 and His marker) and that the presence of repair DNA is responsible for inducing lethality by conducting the deletion properly.

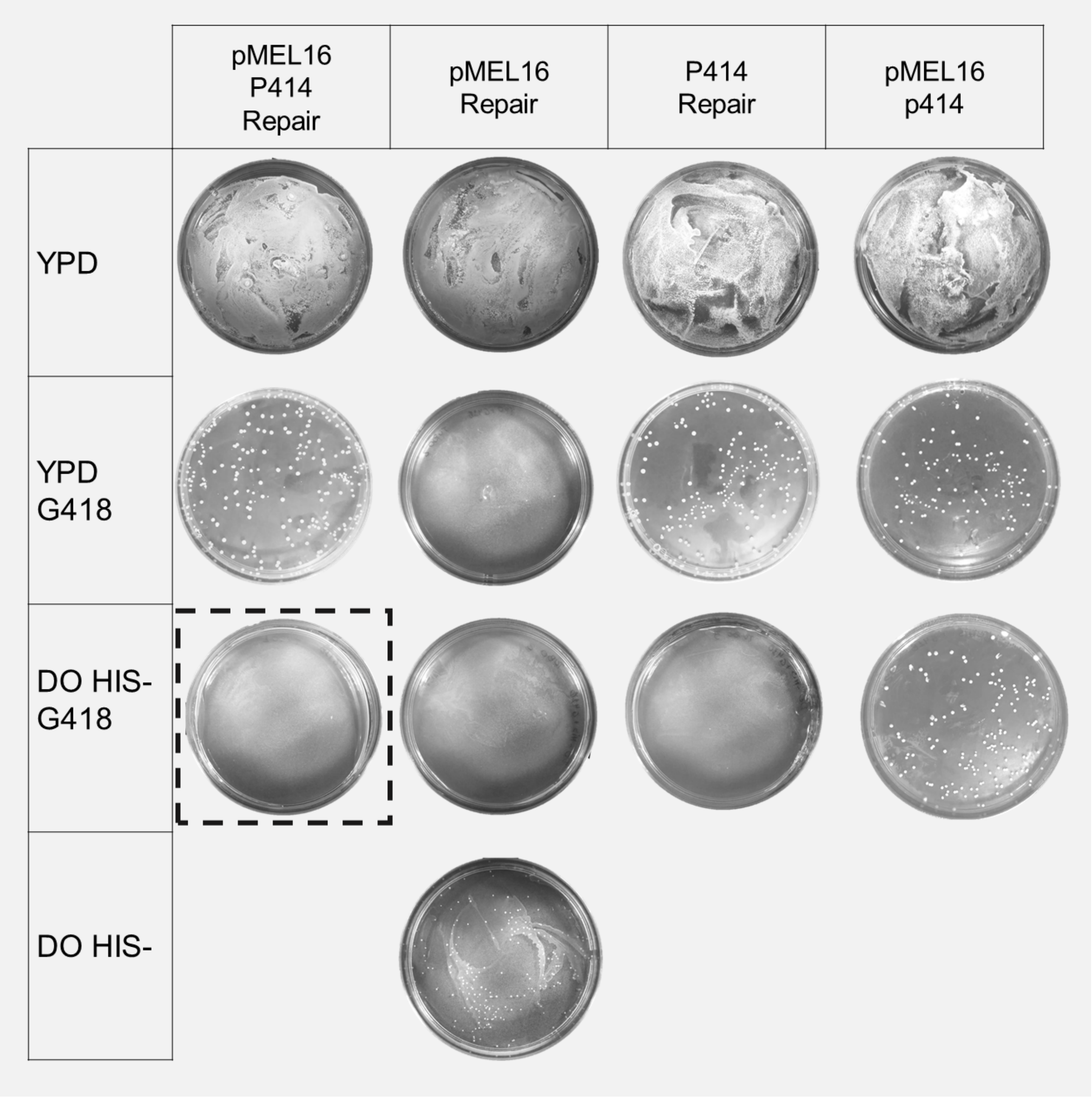
pMEL16 P414 Repair
pMEL16 Repair
P414 Repair
pMEL16 p414
YPD
YPD G418
DO HIS- G418
DO HIS-
