# Supplementary material for: LncRNAs of Saccharomyces cerevisiae bypass the cell cycle arrest imposed by ethanol stress
Source: PLoS Comput Biol. 2022 May 19;18(5):e1010081. doi: 10.1371/journal.pcbi.1010081 (PMC9232138; doi:10.1371/journal.pcbi.1010081)
Supplement: S3 Fig — The simulations were performed using only the node MASS active (level ’1’) as the initial state plus the model constraints created from the expression of DNA damage-related genes (S4 Table and Fig 3E). Notably, lnc10883 was fixed at ’3’ for the in silico overexpression simulation. All simulations related to the HT strains returned a functional cell cycle. Thus, the X-axis represents all states corresponding to the cyclic attractor. Conversely, simulations related to LT strains returned an arrest, as depicted by a steady state with the single-state attractors reported in the last state on the X axis. (PDF) [file pcbi.1010081.s003.pdf]

S3 Fig: Simulations of the DNA damage checkpoint for each strain (the second experimental model simulation) and the role of the lnc10883 in BY4742 in the DNA damage checkpoint (the fourth experimental model simulation). The simulations were performed using only the node MASS active (level '1') as the initial state plus the model constraints created from the expression of DNA damage-related genes (**S4 Table and Fig 3E**). Notably, lnc10883 was fixed at '3' for the *in silico* overexpression simulation. All simulations related to the HT strains returned a functional cell cycle. Thus, the X-axis represents all states corresponding to the cyclic attractor. Conversely, simulations related to LT strains returned an arrest, as depicted by a steady state with the single-state attractors reported in the last state on the X axis.

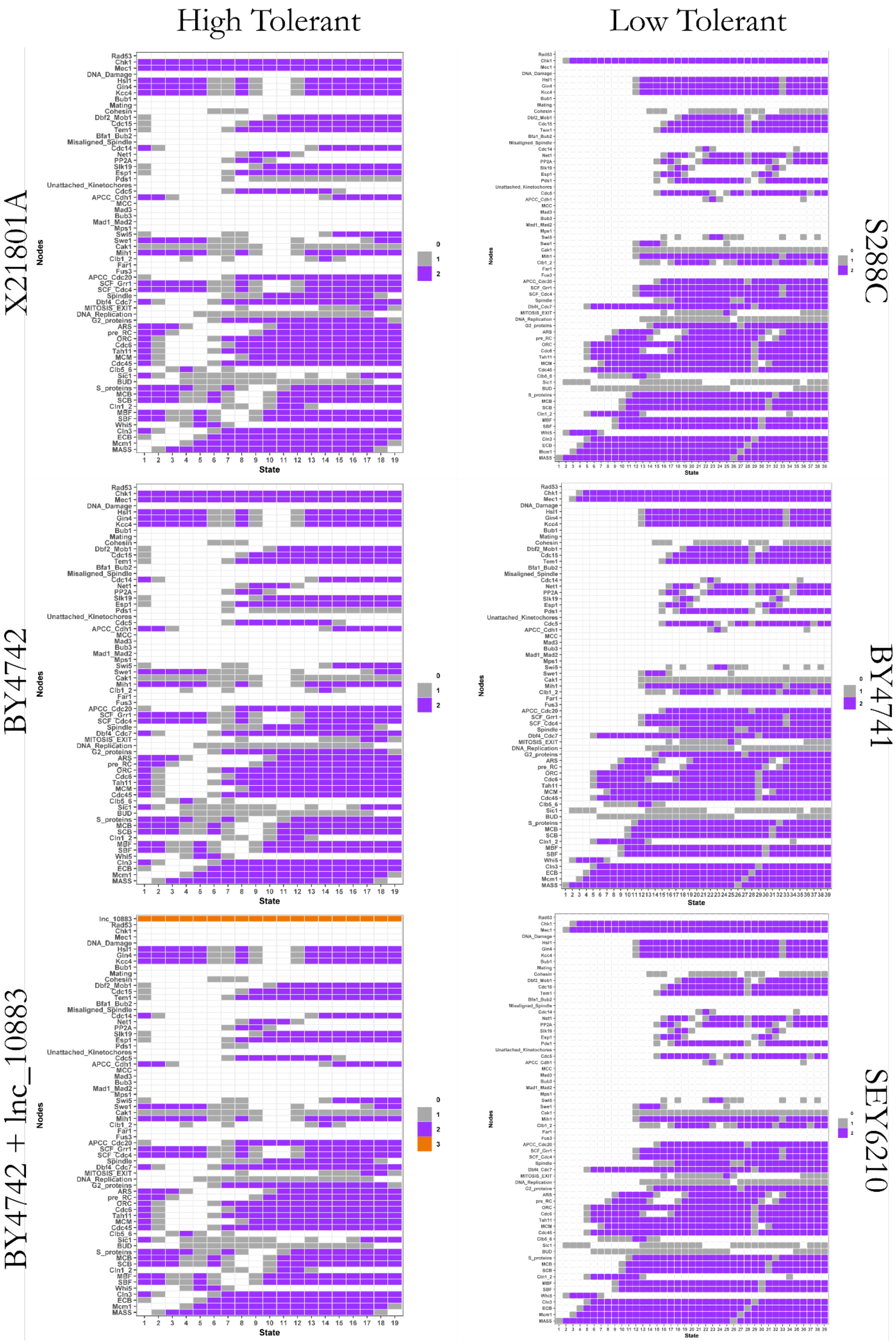
High Tolerant
Low Tolerant
X21801A
BY4742
BY4742 + lnc_10883
S288C
BY4741
SEY6210
Nodes
State
0
1
2
3
