# Supplementary material for: LncRNAs of Saccharomyces cerevisiae bypass the cell cycle arrest imposed by ethanol stress
Source: PLoS Comput Biol. 2022 May 19;18(5):e1010081. doi: 10.1371/journal.pcbi.1010081 (PMC9232138; doi:10.1371/journal.pcbi.1010081)

S4 Fig: Design of repair DNA repair for partial deletions of lnc9136 (A and B) and representation of the Cas9 cut site at the genomic target (the yellow and orange boxes).

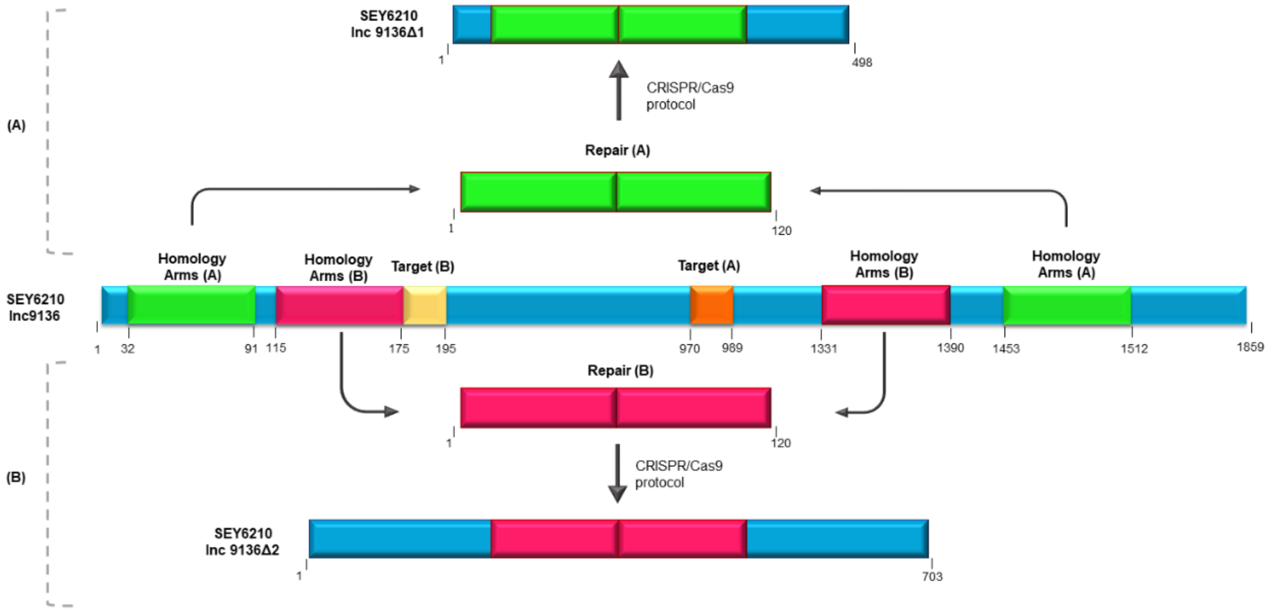

Supplement: S4 Fig — Design of DNA repair for partial deletions of lnc9136 (A and B) and representation of the Cas9 cut site at the genomic target (the yellow and orange boxes). (PDF) [file pcbi.1010081.s004.pdf]
