# Supplementary material for: LncRNAs of Saccharomyces cerevisiae bypass the cell cycle arrest imposed by ethanol stress
Source: PLoS Comput Biol. 2022 May 19;18(5):e1010081. doi: 10.1371/journal.pcbi.1010081 (PMC9232138; doi:10.1371/journal.pcbi.1010081)
Supplement: S1 Table — The references used to design the logic equations are cited in parentheses after each node name. The notation in the equations was designed according to the symbols and rules demanded by the GINsim manual. We highlight details concerning some complex interactions: A) Whi5 is not able to completely inhibit MBF and SBF, even when overexpressed. Then, Whi5 does not arrest the cell cycle, causing only a slight reduction in cell size; B) Inhibition of Clb1_2 relies on the action of the APC complex leading to mitotic exit, which is not performed by Sic1 alone. Thus, at least one part of the APC complex must be active (represented in our model by APCC_Cdc20 or APCC_Cdh1 nodes) to ensure that the model functions for several mutants tested here; C) The Cak1 null mutant is inviable, and information about this gene is scarce. Therefore, Cak1 was modeled here as a Boolean node essential for Clb1_2 activation; D) We stated that Hsl1 and Cdc5 must be active to simulate complete inhibition of Swe1, according to a previous report. However, due to the lack of information in the literature, Kcc4 and Gin4 were modeled as nodes able to reduce Swe1 levels but without inducing complete inhibition; E) CDKs were implicitly present in some nodes, as previously published (Cdc28 is implicit in Clb1_2, Cln5_6, Cln1_2, and Cln3 nodes); F) The Mcm2 protein is implicit in the MCM node; G) ! MASS refers to a MASS = 0 level; H) “*” indicates nodes responsible for activating other nodes usually expressed in these phases; I) Node types (TF, transcription factor; PKC, protein/kinase complex; KI, kinase inhibitor; TR, transcriptional repressor; DRIF, DNA replication initiation factor; DRLF, DNA replication licensing factor; ABP, ATP-binding protein; UPL, ubiquitin-protein ligase; K, kinase; CDKI, cyclin-dependent kinase inhibitor; CDKK, cyclin-dependent kinase-activating kinase; CSCC, component of the spindle-assembly checkpoint complex; KS, kinase substrate; KAP, kinetochore-associated protein; PP, pr [file pcbi.1010081.s005.pdf]

S1 Table: Details of the logical equations for the yeast cell cycle nodes. The references used to design the logic equations are cited in parentheses after each node name. The notation in the equations was designed according to the symbols and rules demanded by the GINsim [1] manual. We highlight details concerning some complex interactions:

A) Whi5 is not able to completely inhibit MBF and SBF, even when overexpressed. Then, Whi5 does not arrest the cell cycle, causing only a slight reduction in cell size [2];

B) Inhibition of Clb1_2 relies on the action of the APC complex leading to mitotic exit, which is not performed by Sic1 alone. Thus, at least one part of the APC complex must be active [3] (represented in our model by APCC_Cdc20 or APCC_Cdh1 nodes) to ensure that the model functions for several mutants tested here;

C) The Cak1 null mutant is inviable [4], and information about this gene is scarce. Therefore, Cak1 was modeled here as a Boolean node essential for Clb1_2 activation;

D) We stated that Hsl1 and Cdc5 must be active to simulate complete inhibition of Swe1, according to a previous report [5]. However, due to the lack of information in the literature, Kcc4 and Gin4 were modeled as nodes able to reduce Swe1 levels but without inducing complete inhibition;

E) CDKs were implicitly present in some nodes, as previously published [6] (Cdc28 is implicit in Clb1_2, Cln5_6, Cln1_2, and Cln3 nodes);

F) The Mcm2 protein is implicit in the MCM node;

G) !MASS refers to a MASS=0 level;

H) "*" indicates nodes responsible for activating other nodes usually expressed in these phases;

I) Node types (TF, transcription factor; PKC, protein/kinase complex; KI, kinase inhibitor; TR, transcriptional repressor; DRIF, DNA replication initiation factor; DRLF, DNA replication licensing factor; ABP, ATP-binding protein; UPL, ubiquitin-protein

ligase; K, kinase; CDKI, cyclin-dependent kinase inhibitor; CDKK, cyclin-dependent kinase-activating kinase; CSCC, component of the spindle-assembly checkpoint complex; KS, kinase substrate; KAP, kinetochore-associated protein; PP, protein phosphatase; CSRC, core subunit of the RENT complex; GAP, GTPase-activating protein; GTP, GTPase; RgEl, regulatory element; PCo, protein complex).

**Node (reference)/node type**	**Value**	**Logical function**
MASS	0	• MITOSIS_EXIT:2
	1	• !MASS & !MITOSIS_EXIT
	2	• MASS:1 | MASS:2
Mcm1/TF	0	• !MASS
	2	• MASS:2
ECB [7]/RegEl	0	• !Mcm1
	1	• Mcm1:1
	2	• Mcm1:2 | Mcm1:3
Cln3 [8]/PKC	0	• !ECB | Far1
	1	• ECB:1
	2	• ECB:2
	3	• ECB:3
Whi5 [9]/TR	0	• Cln3:2 | Cln1_2:2
	2	• (!Cln3 & !Cln1_2) | (Cln1_2:1 & Cln3:1)
SBF [10,11]/TFs	0	• (Whi5:1 & !Cln3 & !Cln1_2 & !Clb5_6) | (Whi5:3 & Cln3:1 & !Cln1_2 & !Clb5_6) | (Whi5:3 & Cln3:1 & Cln1_2:1 & Clb5_6:1) | (Whi5:3 & !Cln3 & Cln1_2:1 & Clb5_6:1) | (Whi5:2 & !Cln3 & !Cln1_2 & !Clb5_6) | (Whi5:3 & !Cln3 & !Cln1_2 & !Clb5_6) | (Whi5:3 & Clb5_6:1 & !Cln1_2 & !Cln3) | (Whi5:3 & Clb5_6:1 & Cln3:1 & !Cln1_2) | (Whi5:3 & Clb5_6:1 & Cln1_2:1 & !Cln3) | (Whi5:3 & !Cln3 & Cln1_2:1 & !Clb5_6)
	1	• (Cln1_2:1 & !Whi5 & Cln3:1) | (Cln1_2:2 & Whi5:2 & Cln3:1) | (Cln1_2:1 & !Whi5 & !Cln3) | (Cln1_2:2 & Whi5:2 & !Cln3) | (Cln1_2:1 & Whi5:2 & Cln3:2) | (!Cln1_2 & !Whi5 & Cln3:1) | (!Cln1_2 & Whi5:2 & Cln3:2) | (Cln1_2:2 & Whi5:3 & Cln3:1) | (Cln1_2:2 & Whi5:3 & Cln3:2) | (Cln1_2:2 & Whi5:3 & Cln3:2) | (Cln1_2:1 & Whi5:3 & Cln3:2) | (Cln1_2:2 & Whi5:3 & Cln3:2) | (Cln1_2:2 & Whi5:3 & !Cln3) | (!Cln1_2 & Whi5:3 & Cln3:2) | (Clb5_6:1 & !Cln1_2 & !Cln3)

	2	• (Cln1_2:2 & !Whi5 & !Cln3) | (Cln1_2:2 & !Whi5 & Cln3:1) | (Cln1_2:2 & !Whi5 & Cln3:2) | (!Cln1_2 & !Whi5 & Cln3:2) | (Cln1_2:1 & !Whi5 & Cln3:2) | (Cln1_2:3 & Whi5:2 & !Cln3) | (Cln1_2:3 & Whi5:2 & Cln3:1) | (Cln1_2:3 & Whi5:2 & Cln3:2) | (Cln1_2:3 & Whi5:2 & Cln3:3) | (Cln1_2:1 & Whi5:2 & Cln3:3) | (Cln1_2:2 & Whi5:2 & Cln3:3) | (!Cln1_2 & Whi5:2 & Cln3:3) | (Clb5_6:2 & !Cln1_2 & !Cln3)
	3	• (Cln3:3 & !Whi5 & !Cln1_2) | (Cln3:3 & !Whi5 & Cln1_2:1) | (Cln3:3 & !Whi5 & Cln1_2:2) | (Cln3:3 & !Whi5 & Cln1_2:3) | (!Cln3 & !Whi5 & Cln1_2:3) | (Cln3:1 & !Whi5 & Cln1_2:3) | (Cln3:2 & !Whi5 & Cln1_2:3) | (!Whi5 & Clb5_6:3 & !Cln1_2 & !Cln3)
MBF [10,11]/TFs	0	• (Whi5:1 & !Cln3 & !Cln1_2 & !Clb5_6) | (Whi5:3 & Cln3:1 & !Cln1_2 & !Clb5_6) | (Whi5:3 & Cln3:1 & Cln1_2:1 & Clb5_6:1) | (Whi5:3 & !Cln3 & Cln1_2:1 & Clb5_6:1) | (Whi5:2 & !Cln3 & !Cln1_2 & !Clb5_6) | (Whi5:3 & !Cln3 & !Cln1_2 & !Clb5_6) | (Whi5:3 & Clb5_6:1 & !Cln1_2 & !Cln3) | (Whi5:3 & Clb5_6:1 & Cln3:1 & !Cln1_2) | (Whi5:3 & Clb5_6:1 & Cln1_2:1 & !Cln3) | (Whi5:3 & !Cln3 & Cln1_2:1 & !Clb5_6)
	1	• (Cln1_2:1 & !Whi5 & Cln3:1) | (Cln1_2:2 & Whi5:2 & Cln3:1) | (Cln1_2:1 & !Whi5 & !Cln3) | (Cln1_2:2 & Whi5:2 & !Cln3) | (Cln1_2:1 & Whi5:2 & Cln3:2) | (!Cln1_2 & !Whi5 & Cln3:1) | (!Cln1_2 & Whi5:2 & Cln3:2) | (Cln1_2:2 & Whi5:3 & Cln3:1) | (Cln1_2:2 & Whi5:3 & Cln3:2) | (Cln1_2:2 & Whi5:3 & Cln3:2) | (Cln1_2:1 & Whi5:3 & Cln3:2) | (Cln1_2:2 & Whi5:3 & Cln3:2) | (Cln1_2:2 & Whi5:3 & !Cln3) | (!Cln1_2 & Whi5:3 & Cln3:2) | (Clb5_6:1 & !Cln1_2 & !Cln3)
	2	• (Cln1_2:2 & !Whi5 & !Cln3) | (Cln1_2:2 & !Whi5 & Cln3:1) | (Cln1_2:2 & !Whi5 & Cln3:2) | (!Cln1_2 & !Whi5 & Cln3:2) | (Cln1_2:1 & !Whi5 & Cln3:2) | (Cln1_2:3 & Whi5:2 & !Cln3) | (Cln1_2:3 & Whi5:2 & Cln3:1) | (Cln1_2:3 & Whi5:2 & Cln3:2) | (Cln1_2:3 & Whi5:2 & Cln3:3) | (Cln1_2:1 & Whi5:2 & Cln3:3) | (Cln1_2:2 & Whi5:2 & Cln3:3) | (!Cln1_2 & Whi5:2 & Cln3:3) | (Clb5_6:2 & !Cln1_2 & !Cln3)
	3	• (Cln3:3 & !Whi5 & !Cln1_2) | (Cln3:3 & !Whi5 & Cln1_2:1) | (Cln3:3 & !Whi5 & Cln1_2:2) | (Cln3:3 & !Whi5 & Cln1_2:3) | (!Cln3 & !Whi5 & Cln1_2:3) | (Cln3:1 & !Whi5 & Cln1_2:3) | (Cln3:2 & !Whi5 & Cln1_2:3) | (Clb5_6:3 & !Cln1_2 & !Cln3)
Cln1_2 [12]/PKC	0	• Far1:1 | (SCF_Grr1 & !SCB & !ECB) | (SCF_Grr1:3 & SCB & ECB) | (SCF_Grr1:2 & SCB:2 & ECB:2) | (SCF_Grr1:2 & SCB:2 & ECB:1) | (SCF_Grr1:2 & SCB:1 & ECB:2) | (SCF_Grr1:1 & SCB:1 & ECB:1) | SCF_Grr1:3

	1	• (ECB:1 & SCB:1 & !SCF_Grr1) | (ECB:1 & !SCB & !SCF_Grr1) | (SCB:1 & !ECB & !SCF_Grr1) | (ECB:2 & SCB:2 & SCF_Grr1:1) | (ECB:2 & SCB:1 & SCF_Grr1:1) | (SCB:2 & ECB:1 & SCF_Grr1:1) | (ECB:2 & !SCB & SCF_Grr1:1) | (SCB:2 & !ECB & SCF_Grr1:1) | (ECB:3 & SCB:1 & SCF_Grr1:2) | (ECB:3 & SCB:2 & SCF_Grr1:2) | (ECB:3 & SCB:3 & SCF_Grr1:2) | (ECB:1 & SCB:3 & SCF_Grr1:2) | (ECB:2 & SCB:3 & SCF_Grr1:2)
	2	• (ECB:2 & SCB:2 & !SCF_Grr1) | (ECB:2 & !SCB & !SCF_Grr1) | (SCB:2 & !ECB & !SCF_Grr1) | (ECB:3 & SCB:3 & SCF_Grr1:1) | (ECB:3 & SCB:1 & SCF_Grr1:1) | (ECB:3 & SCB:2 & SCF_Grr1:1) | (ECB:1 & SCB:3 & SCF_Grr1:1) | (ECB:2 & SCB:3 & SCF_Grr1:1) | (ECB:3 & !SCB & SCF_Grr1:1) | (!ECB & SCB:3 & SCF_Grr1:1) | (ECB:1 & SCB:2 & !SCF_Grr1) | (ECB:2 & SCB:1 & !SCF_Grr1)
	3	• (ECB:3 & SCB:3 & !SCF_Grr1) | (ECB:3 & !SCB & !SCF_Grr1) | (SCB:3 & !ECB & !SCF_Grr1) | (ECB:3 & SCB:1 & !SCF_Grr1) | (ECB:3 & SCB:2 & !SCF_Grr1) | (SCB:3 & ECB:1 & !SCF_Grr1) | (SCB:3 & ECB:2 & !SCF_Grr1)
SCB[13]/RegEl	1	• SBF:1 | (MBF:1 & SBF:1)
	2	• SBF:2 | SBF:3 | (MBF:3 & SBF:3)
MCB [13]/RegEl	1	• (MBF:1 & SBF:1) | SBF:1
	2	• SBF:2 | SBF:3 | (MBF:3 & SBF:3)
S_proteins*	1	• SCB:1 & MCB:1
	2	• (SCB:2 & MCB:2) | (SCB:3 & MCB:3)
BUD [14]	0	• MITOSIS_EXIT:2
	1	• Cln1_2 | (Clb5_6 & !Cln1_2) | BUD:1
Sic1 [15–18]/KI	1	• (!Swi5 & !Cdc14 & !Cln1_2 & !SCF_Cdc4) | (Swi5:1 & !Cln1_2 & !Cdc14) | (Swi5:1 & Cln1_2 & Cdc14:3) | (Swi5:1 & (Cln1_2:1 | Cln1_2:2) & Cdc14:2) | (Swi5:1 & Cln1_2:1 & !Cdc14) | (SCF_Cdc4:1 & Swi5:1 & Cln1_2 & !Cdc14) | (SCF_Cdc4:2 & Swi5:2 & Cln1_2 & !Cdc14) | (SCF_Cdc4:3 & Swi5 & Cln1_2 & !Cdc14) | (SCF_Cdc4 & (!Cln1_2 | Cln1_2) & !Swi5 & !Cdc14)
	2	• (Swi5:2 & !Cln1_2 & !Cdc14 & !SCF_Cdc4) | (Swi5:2 & Cln1_2 & Cdc14:3 & SCF_Cdc4) | (Swi5:2 & Cln1_2:1 & Cdc14:1 & (!SCF_Cdc4 | SCF_Cdc4:1)) | (Swi5:2 & Cln1_2:2 & Cdc14:2 & (!SCF_Cdc4 | SCF_Cdc4:1 | SCF_Cdc4:2)) | (Swi5:3 & Cln1_2:1 & !Cdc14 & !SCF_Cdc4) | (Swi5:3 & Cln1_2:2 & Cdc14:1 & (!SCF_Cdc4 | SCF_Cdc4:1)) | (Swi5:3 & Cln1_2:3 & Cdc14:2 & (!SCF_Cdc4 | SCF_Cdc4:1 | SCF_Cdc4:2)) | (Swi5:2 & Cdc14 & !Cln1_2 & !SCF_Cdc4) | (Swi5:2 & Cdc14:1 & !Cln1_2 & (SCF_Cdc4:1 | !SCF_Cdc4)) | (Swi5:2 & Cdc14:2 & !Cln1_2 & (!SCF_Cdc4 | SCF_Cdc4:1 | SCF_Cdc4:2)) | (Swi5:2 & Cdc14:3 & !Cln1_2 &

		SCF_Cdc4) | (Swi5:2 & Cdc14:2 & (Cln1_2:1 | Cln1_2:2 | !Cln1_2)) | (Swi5:3 & (SCF_Cdc4:1 | SCF_Cdc4:2) & !Cdc14 & !Cln1_2)
	3	• (Swi5:3 & Cdc14:3 & (Cln1_2 | !Cln1_2) & (SCF_Cdc4 | !SCF_Cdc4)) | (Swi5:3 & Cdc14 & !Cln1_2 & (!SCF_Cdc4 | SCF_Cdc4)) | (Swi5:3 & !Cdc14 & !Cln1_2 & !SCF_Cdc4) | (Swi5:3 & Cdc14:2 & (Cln1_2:1 | Cln1_2:2 | !Cln1_2) & (!SCF_Cdc4 | SCF_Cdc4:1))
Clb5_6 [11,19,20]/PKC	0	• Sic1:3 | APCC_Cdc20:3 | (Sic1:3 & APCC_Cdc20:3) | (Sic1:2 & APCC_Cdc20:2) | (Sic1 & APCC_Cdc20 & !MCB) | (MCB:1 & APCC_Cdc20:1 & Sic1) | (MCB:1 & APCC_Cdc20 & Sic1:1) | (MCB:1 & APCC_Cdc20:2 & Sic1) | (MCB:1 & APCC_Cdc20 & Sic1:2) | (MCB:1 & APCC_Cdc20 & Sic1:3) | (MCB:1 & APCC_Cdc20:3 & Sic1)
	1	• (MCB:1 & !Sic1 & !APCC_Cdc20) | (MCB:2 & Sic1:1 & !APCC_Cdc20) | (MCB:2 & !Sic1 & APCC_Cdc20:1) | (MCB:3 & Sic1:1 & APCC_Cdc20:1) | (MCB:3 & Sic1:2 & !APCC_Cdc20) | (MCB:3 & !Sic1 & APCC_Cdc20:2) | (ECB:1 & !Sic1 & !APCC_Cdc20 & !MCB)
	2	• (MCB:2 & !Sic1 & !APCC_Cdc20) | (MCB:3 & Sic1:1 & !APCC_Cdc20) | (MCB:3 & !Sic1 & APCC_Cdc20:1)
	3	• MCB:3 & !Sic1 & !APCC_Cdc20
Cdc45 [21]/DRIF	0	• !ECB
	1	• ECB:1
	2	• ECB:2
	3	• ECB:3
MCM [22]/PCo	1	• ECB:1 & Dbf4_Cdc7:1
	2	• ECB:2 & Dbf4_Cdc7:2
	3	• ECB:3 & Dbf4_Cdc7:3
Tah11 [23]/DRLF	0	• !ECB
	1	• ECB:1
	2	• ECB:2
	3	• ECB:3
Cdc6 [24]/ABP	0	• (SCF_Cdc4:3 & ECB) | (Clb5_6:3 & ECB) | (SCF_Cdc4:2 & Clb5_6:2)
	1	• ECB:1 & (!Clb5_6 | (Clb5_6:1 & SCF_Cdc4:1) | (Clb5_6:1 & !SCF_Cdc4) | (SCF_Cdc4:1 & !Clb5_6) | (Clb5_6:2 & !SCF_Cdc4) | (SCF_Cdc4:2 & !Clb5_6) | !SCF_Cdc4)
	2	• ECB:2 & (!Clb5_6 | (Clb5_6:1 & SCF_Cdc4:1) | (Clb5_6:1 & !SCF_Cdc4) | (SCF_Cdc4:1 & !Clb5_6) | (Clb5_6:2 & !SCF_Cdc4) | (SCF_Cdc4:2 & !Clb5_6) | !SCF_Cdc4)

	3	• ECB:3 & (!Clb5_6 | (Clb5_6:1 & SCF_Cdc4:1) | (Clb5_6:1 & !SCF_Cdc4) | (SCF_Cdc4:1 & !Clb5_6) | (Clb5_6:2 & !SCF_Cdc4) | (SCF_Cdc4:2 & !Clb5_6) | !SCF_Cdc4)
ORC [25]/PCo	0	• !ECB
	1	• ECB:1
	2	• ECB:2
	3	• ECB:3
pre_RC [21,22,26,27]/PCo	1	• (Cdc45:1 & Tah11:1 & Cdc6:1 & MCM:1 & ORC:1) | (Cdc45:1 & Tah11 & Cdc6 & ORC & MCM) | (Cdc45 & Tah11:1 & Cdc6 & ORC & MCM) | (Cdc45 & Tah11 & Cdc6:1 & ORC & MCM) | (Cdc45 & Tah11 & Cdc6 & ORC:1 & MCM) | (Cdc45 & Tah11 & Cdc6 & ORC & MCM:1)
	2	• (Cdc45:2 & Tah11:2 & Cdc6:2 & ORC:2 & MCM:2) | (Cdc45:2 & (Tah11:3 | Tah11:2) & (Cdc6:3 | Cdc6:2) & (ORC:3 | ORC:2) & (MCM:3 | MCM:2)) | ((Cdc45:3 | Cdc45:2) & Tah11:2 & (Cdc6:3 | Cdc6:2) & (ORC:3 | ORC:2) & (MCM:3 | MCM:2)) | ((Cdc45:3 | Cdc45:2) & (Tah11:3 | Tah11:2) & Cdc6:2 & (ORC:3 | ORC:2) & (MCM:3 | MCM:2)) | ((Cdc45:3 | Cdc45:2) & (Tah11:3 | Tah11:2) & (Cdc6:3 | Cdc6:2) & ORC:2 & (MCM:3 | MCM:2)) | ((Cdc45:3 | Cdc45:2) & (Tah11:3 | Tah11:2) & (Cdc6:3 | Cdc6:2) & (ORC:3 | ORC:2) & MCM:2)
	3	• Cdc45:3 & Tah11:3 & Cdc6:3 & ORC:3 & MCM:3
ARS [25]/RegEl	1	• pre_RC:1
	2	• pre_RC:2
	3	• pre_RC:3
G2_proteins*	2	• DNA_Replication:1
DNA_Replication [28]	0	• MITOSIS_EXIT:2
	1	• ((Clb1_2:2 | Clb1_2:3) & ARS) | ((Clb5_6:2 | Clb5_6:3) & ARS) | DNA_Replication:1 | (Clb1_2:1 & Clb5_6:1 & ARS) | (Clb1_2:1 & !Clb5_6 & ARS)
MITOSIS_EXIT [29]	0	• !Spindle | !BUD | !DNA_Replication
	1	• (Spindle:2 | Spindle:1) & BUD:1 & DNA_Replication:1 & (Clb1_2:1 | Clb1_2:2 | Clb1_2:3)
	2	• Spindle:2 & BUD:1 & DNA_Replication:1 & !Clb1_2 & MITOSIS_EXIT:1
Dbf4_Cdc7 (KEGG)/PKC	0	• !ECB
	1	• ECB:1
	2	• ECB:2
	3	• ECB:3
Spindle [30]	0	• MITOSIS_EXIT:2

	1	• Cohesin:1 & (!MITOSIS_EXIT | MITOSIS_EXIT:1) & (!Spindle | Spindle:1)
	2	• (Spindle:1 & !Cohesin & (!MITOSIS_EXIT | MITOSIS_EXIT:1)) | (Spindle:2 & (MITOSIS_EXIT:1 | !MITOSIS_EXIT) & (Cohesin | !Cohesin))
SCF_Cdc4 [25]/UPL	0	• !S_proteins
	1	• S_proteins:1
	2	• S_proteins:2
	3	• S_proteins:3
SCF_Grr1 [25]/UPL	0	• !S_proteins
	1	• S_proteins:1
	2	• S_proteins:2
	3	• S_proteins:3
APCC_Cdc20 [31]/UPL	0	• MCC:1
	1	• G2_proteins:1 & !MCC
	2	• G2_proteins:2 & !MCC
	3	• G2_proteins:3 & !MCC
Fus3 [32]/PK	0	• !Mating
	1	• Mating
Far1 [32,33]/CDKI	0	• !Fus3
		• SCF_Cdc4
	1	• Fus3 & !SCF_Cdc4
Clb1_2 [19,34–36]/PKC	0	• !Cak1 | !MCB | Swe1:3 | APCC_Cdc20:3 | APCC_Cdh1:3 | Sic1:3 | (MCB:2 & Cak1 & APCC_Cdh1:2) | (MCB:2 & Cak1 & ((Sic1:2 & APCC_Cdc20:1) | (Sic1:2 & APCC_Cdc20:2))) | (MCB:2 & Cak1 & ((Swe1:2 & APCC_Cdc20:1) | (Swe1:2 & APCC_Cdc20:2))) | (MCB:1 & Cak1 & APCC_Cdh1:1) | (MCB:1 & Cak1 & Swe1 & APCC_Cdc20)
	1	• (MCB:1 & Cak1 & !APCC_Cdh1 & (!Sic1 | (Sic1:1 & !APCC_Cdc20) | (Sic1:2 & !APCC_Cdc20)) & !Swe1 & (!APCC_Cdc20 | APCC_Cdc20:1 | APCC_Cdc20:2)) | (MCB:2 & Cak1 & APCC_Cdh1:1 & (!APCC_Cdc20 | APCC_Cdc20:1 | APCC_Cdc20:2)) | (MCB:2 & Cak1 & Swe1:1 & (!APCC_Cdc20 | APCC_Cdc20:1 | APCC_Cdc20:2)) | (MCB:1 & Cak1 & !APCC_Cdh1 & ((Sic1:1 & APCC_Cdc20:1) | (Sic1:1 & APCC_Cdc20:2)) & (!APCC_Cdc20 | APCC_Cdc20:1 | APCC_Cdc20:2) & !Swe1) | (MCB:2 & Cak1 & !APCC_Cdc20 & Swe1:2 & !APCC_Cdh1)

	2	• (MCB:2 & Cak1 & !APCC_Cdh1 & (!Sic1 | (Sic1:1 & !APCC_Cdc20) | (Sic1:2 & !APCC_Cdc20)) & (!APCC_Cdc20 | APCC_Cdc20:1 | APCC_Cdc20:2) & !Swe1) | (MCB:3 & Cak1 & APCC_Cdh1:1 & (!APCC_Cdc20 | APCC_Cdc20:1 | APCC_Cdc20:2)) | (MCB:3 & Cak1 & Swe1:1 & (!APCC_Cdc20 | APCC_Cdc20:1 | APCC_Cdc20:2)) | (MCB:2 & Cak1 & !APCC_Cdh1 & ((Sic1:1 & APCC_Cdc20:1) | (Sic1:1 & APCC_Cdc20:2)) & (!APCC_Cdc20 | APCC_Cdc20:1 | APCC_Cdc20:2) & !Swe1) | (MCB:3 & Cak1 & APCC_Cdh1:2 & (!APCC_Cdc20 | APCC_Cdc20:1 | APCC_Cdc20:2)) | (MCB:3 & Cak1 & ((Sic1:2 & APCC_Cdc20:1) | (Sic1:2 & APCC_Cdc20:2)) & (!APCC_Cdc20 | APCC_Cdc20:1 | APCC_Cdc20:2)) | (MCB:3 & Cak1 & Swe1:2 & (!APCC_Cdc20 | APCC_Cdc20:1 | APCC_Cdc20:2))
	3	• (MCB:3 & Cak1 & !APCC_Cdh1 & (!Sic1 | (Sic1:1 & !APCC_Cdc20) | (Sic1:2 & !APCC_Cdc20)) & (!APCC_Cdc20 | APCC_Cdc20:1 | APCC_Cdc20:2) & !Swe1) | (MCB:3 & Cak1 & !APCC_Cdh1 & ((Sic1:1 & APCC_Cdc20:1) | (Sic1:1 & APCC_Cdc20:2)) & (!APCC_Cdc20 | APCC_Cdc20:1 | APCC_Cdc20:2) & !Swe1)
Cak1 [31]/CDKK	1	• S_proteins
Swe1 [37,38]/K	0	• (S_proteins & Hsl1:3 & Cdc5:3 & (Gin4 | !Gin4) & (!Kcc4 | Kcc4)) | (S_proteins:1 & Hsl1:1 & Cdc5 & (Gin4 | !Gin4) & (!Kcc4 | Kcc4)) | (S_proteins:1 & Hsl1 & Cdc5:1 & (Gin4 | !Gin4) & (!Kcc4 | Kcc4)) | (S_proteins:2 & Hsl1:2 & (Cdc5:2 | Cdc5:3) & (Gin4 | !Gin4) & (!Kcc4 | Kcc4)) | (S_proteins:2 & (Hsl1:2 | Hsl1:3) & Cdc5:2 & (Gin4 | !Gin4) & (!Kcc4 | Kcc4))
	1	• (S_proteins:1 & !Hsl1 & !Cdc5 & !Kcc4 & !Gin4) | (S_proteins:1 & ((Hsl1:2 & !Cdc5) | (Hsl1:1 & !Cdc5) | (!Hsl1 & Cdc5:1) | (!Hsl1 & Cdc5:2)) & (Kcc4:1 | !Kcc4 | Kcc4:2) & (Gin4:1 | !Gin4 | Gin4:2)) | (S_proteins:2 & Hsl1:1 & Cdc5:1 & (Kcc4:1 | Kcc4:2 | !Kcc4) & (Cdc5:1 | Cdc5:2 | !Cdc5)) | (S_proteins:2 & Kcc4:3 & Gin4:3 & (Hsl1:1 | !Hsl1) & (!Cdc5 | Cdc5:1))
	2	• (S_proteins:2 & !Hsl1 & !Cdc5 & !Kcc4 & !Gin4) | (S_proteins:2 & ((Hsl1:2 & !Cdc5) | (Hsl1:1 & !Cdc5) | (!Hsl1 & Cdc5:1) | (!Hsl1 & Cdc5:2)) & (Kcc4:1 | !Kcc4 | Kcc4:2) & (Gin4:1 | !Gin4 | Gin4:2)) | (S_proteins:3 & ((Hsl1:1 & Cdc5:1) | (Hsl1:2 & Cdc5:1) | (Hsl1:1 & Cdc5:2)) & (Kcc4:1 | Kcc4:2 | !Kcc4) & (Gin4:1 | Gin4:2 | !Gin4)) | (S_proteins:3 & Kcc4:2 & Gin4:3 & (Hsl1:1 | !Hsl1) & (Cdc5:1 | !Cdc5)) | (S_proteins:3 & Kcc4:3 & Gin4:2 & (Hsl1:1 | !Hsl1) & (Cdc5:1 | !Cdc5)) | (S_proteins:3 & Hsl1:2 & Cdc5:2 & (Kcc4:1 | Kcc4:2 | !Kcc4) & (Cdc5:1 | Cdc5:2 | !Cdc5))

	3	• (S_proteins:3 & !Hsl1 & !Cdc5 & !Kcc4 & !Gin4) | (S_proteins:3 & ((Hsl1:2 & !Cdc5) | (Hsl1:1 & !Cdc5) | (!Hsl1 & Cdc5:1) | (!Hsl1 & Cdc5:2)) & (Kcc4:1 | !Kcc4 | Kcc4:2) & (Gin4:1 | !Gin4 | Gin4:2))
Swi5 [39]/TF	0	• (G2_proteins:1 & Cdc14:1 & Clb1_2:2) | (G2_proteins:1 & Cdc14:1 & Clb1_2:3) | (G2_proteins:1 & Cdc14:2 & Clb1_2:3) | (G2_proteins:2 & Cdc14:1 & Clb1_2:3) | (G2_proteins:2 & !Cdc14 & Clb1_2:2) | (G2_proteins:1 & !Cdc14 & Clb1_2:3) | (G2_proteins:3 & !Cdc14 & Clb1_2:3) | (G2_proteins:1 & !Cdc14 & Clb1_2:2) | (G2_proteins:1 & !Cdc14 & Clb1_2:3)
	1	• (G2_proteins:1 & !Cdc14 & !Clb1_2) | (G2_proteins:1 & Cdc14:1 & Clb1_2:1) | (G2_proteins:1 & Cdc14:2 & Clb1_2:2) | (G2_proteins:1 & Cdc14:3 & Clb1_2) | (G2_proteins:2 & Cdc14:1 & Clb1_2:2) | (G2_proteins:2 & Cdc14:2 & Clb1_2:3) | (G2_proteins:3 & Cdc14:1 & Clb1_2:3) | (G2_proteins:2 & !Cdc14 & Clb1_2:1) | (G2_proteins:1 & Cdc14 & !Clb1_2) | (G2_proteins:1 & Cdc14:2 & Clb1_2:1)
	2	• (G2_proteins:2 & Cdc14 & !Clb1_2) | (G2_proteins:2 & Cdc14:1 & Clb1_2:1) | (G2_proteins:2 & Cdc14:2 & Clb1_2:1) | (G2_proteins:2 & Cdc14:2 & Clb1_2:2) | (G2_proteins:2 & Cdc14:3 & Clb1_2) | (G2_proteins:3 & Cdc14:1 & Clb1_2:2) | (G2_proteins:3 & Cdc14:2 & Clb1_2:3)
	3	• (G2_proteins:3 & !Cdc14 & !Clb1_2) | (G2_proteins:3 & Cdc14:3 & Clb1_2) | (G2_proteins:3 & Cdc14:1 & Clb1_2:1) | (G2_proteins:3 & Cdc14:2 & Clb1_2:2) | (G2_proteins:3 & Cdc14:2 & Clb1_2:1)
Mps1 [40]/K	0	• !Unattached_Kinetochores
	1	• Unattached_Kinetochores:1
	2	• Unattached_Kinetochores:2
Mad1_Mad2 [40]/CSCC	0	• !Mps1
	1	• Mps1:1
	2	• Mps1:2
Bub3 [40]/KS	0	• !Unattached_Kinetochores
	1	• Unattached_Kinetochores:1
	2	• Unattached_Kinetochores:2
Mad3 [40]/CSCC	0	• !Unattached_Kinetochores
	1	• Unattached_Kinetochores:1
	2	• Unattached_Kinetochores:2
MCC [40]/PCo	0	• (Spindle:1 & Bub1:1 & Mad1_Mad2:1 & Bub3:1 & Mad3:1) | (Spindle:2 & Bub1:1 & Mad1_Mad2:1 & Bub3:1 & Mad3:1)
	1	• (Mad1_Mad2:1 & Mad3:1 & Bub3:1 & Bub1:1 & !Spindle) | (Mad1_Mad2:2 & Mad3:2 & Bub3:2 & Bub1:2)

APCC_Cdh1[36]/ UPL	0	• !Cdc14
	1	• Cdc14:1
	2	• Cdc14:2
	3	• Cdc14:3
Cdc5 [41]/K	1	• (G2_proteins:1 & !APCC_Cdh1) | (G2_proteins:2 & APCC_Cdh1:1) | (G2_proteins:3 & APCC_Cdh1:2)
	2	• (G2_proteins:2 & !APCC_Cdh1) | (G2_proteins:3 & APCC_Cdh1:1)
	3	• G2_proteins:3 & !APCC_Cdh1
Unattached_Kinetochores		Input
Pds1 [42]/Securin	0	• (G2_proteins:1 & APCC_Cdc20 & !Chk1) | (G2_proteins:2 & APCC_Cdc20:2 & !Chk1) | (G2_proteins & APCC_Cdc20:3 & !Chk1)
	1	• (G2_proteins:1 & !APCC_Cdc20) | (G2_proteins:2 & APCC_Cdc20:1 & !Chk1) | (G2_proteins:3 & APCC_Cdc20:2 & !Chk1)
	2	• (G2_proteins:2 & !APCC_Cdc20) | (G2_proteins:3 & APCC_Cdc20:1) | (G2_proteins:2 & !APCC_Cdc20 & Chk1) | (G2_proteins:3 & APCC_Cdc20 & Chk1) | (G2_proteins:2 & APCC_Cdc20:2 & Chk1:2)
	3	• (G2_proteins:3 & !APCC_Cdc20 & !Chk1) | (G2_proteins:3 & !APCC_Cdc20 & Chk1)
Esp1 [42]/Separin	0	• (Pds1:3 & G2_proteins) | (Pds1:2 & (G2_proteins:1 | G2_proteins:2))
	1	• (G2_proteins:1 & !Pds1) | (G2_proteins:1 & Pds1:1)
	2	• (G2_proteins:2 & (!Pds1 | Pds1:1)) | (G2_proteins:3 & Pds1:2)
	3	• (G2_proteins:3 & !Pds1) | (G2_proteins:3 & Pds1:1)
Slk19 [42]/KAP	1	• Esp1:1 & G2_proteins:1
	2	• Esp1:2 & G2_proteins:2
	3	• Esp1:3 & G2_proteins:3
PP2A [25]/PP	0	• (G2_proteins:1 & Slk19) | (G2_proteins:2 & Slk19:2) | (G2_proteins:2 & Slk19:3) | (G2_proteins & Slk19:3)
	1	• (G2_proteins:1 & !Slk19) | (G2_proteins:2 & Slk19:1) | (G2_proteins:3 & Slk19:2)
	2	• (G2_proteins:2 & !Slk19) | (G2_proteins:3 & Slk19:1)
	3	• G2_proteins:3 & !Slk19
Net1 [25]/CSRC	0	• !G2_proteins | !PP2A
	1	• PP2A & G2_proteins:1
	2	• PP2A & G2_proteins:2
	3	• PP2A & G2_proteins:3
Cdc14 [42]/PP	0	• (Net1:3 & Dbf2_Mob1:1) | (Net1:3 & Dbf2_Mob1:2) | (!Dbf2_Mob1 & Net1:1) | (Net1 & !Dbf2_Mob1) | (Net1:1 &

		Dbf2_Mob1:1) | (Net1:2 & Dbf2_Mob1:2) | (Net1:3 & Dbf2_Mob1:3)
	1	• (Dbf2_Mob1:1 & !Net1) | (Dbf2_Mob1:2 & Net1:1)
	2	• (Dbf2_Mob1:2 & !Net1) | (Dbf2_Mob1:3 & Net1:2)
	3	• Dbf2_Mob1:3 & !Net1
Misaligned_Spindle		Input
Bfa1_Bub2 [43]/GAP	0	• (Cdc5:2 & Misaligned_Spindle) | (Cdc5:3 & Misaligned_Spindle) | (Spindle:1 & Misaligned_Spindle:1) | (Spindle:2 & Misaligned_Spindle:1)
	1	• Misaligned_Spindle:1 & !Spindle & !Cdc5
	2	• Misaligned_Spindle:2 & !Cdc5
Tem1 [44]/GTP	0	• Bfa1_Bub2:1 | Bfa1_Bub2:2
	1	• G2_proteins:1 & !Bfa1_Bub2
	2	• G2_proteins:2 & !Bfa1_Bub2
	3	• G2_proteins:3 & !Bfa1_Bub2
Cdc15 [45]/K	0	• Rad53:1
	1	• Tem1 & G2_proteins:1
	2	• Tem1 & G2_proteins:2
	3	• Tem1 & G2_proteins:3
Dbf2_Mob1 [42]/K	1	• Cdc15:1 & G2_proteins:1
	2	• Cdc15:2 & G2_proteins:2
	3	• Cdc15:3 & G2_proteins:3
Cohesin [42]/protein	0	• Esp1:2 | Esp1:3 | !DNA_Replication
	1	• DNA_Replication & (!Esp1 | Esp1:1)
Mating		Input
Bub1 [25]/K	0	• lnc_10883:3 | (Unattached_Kinetochores:1 & lnc_10883:2)
	1	• (Unattached_Kinetochores:1 & !lnc_10883) | (Unattached_Kinetochores:2 & (lnc_10883:1 | lnc_10883:2))
	2	• Unattached_Kinetochores:2
Kcc4 [25]/K	0	• !S_proteins
	1	• S_proteins:1
	2	• S_proteins:2
	3	• S_proteins:3
Gin4 [25]/K	0	• !S_proteins | lnc_9136:3 | (S_proteins:1 & lnc_9136) | (S_proteins:2 & lnc_9136:2)
	1	• S_proteins:1 & !lnc_9136
	2	• (S_proteins:2 & !lnc_9136) | (S_proteins:2 & !lnc_9136) | (S_proteins:2 & lnc_9136:1) | (S_proteins:3 & lnc_9136:2)
	3	• S_proteins:3 & !lnc_9136
Hsl1 [25]/K	0	• !S_proteins | (S_proteins & lnc_9136:3) | (S_proteins:1 & lnc_9136) | (S_proteins:2 & lnc_9136:2)
	1	• S_proteins:1 & !lnc_9136

	2	• (S_proteins:2 & !lnc_9136) | (S_proteins:2 & lnc_9136:1) | (S_proteins:3 & lnc_9136:2)
	3	• S_proteins:3 & !lnc_9136
DNA_Damage		Input
Mec1 [25]/K	0	• !DNA_Damage | lnc_10883:3
	1	• DNA_Damage:1
	2	• DNA_Damage:2
Chk1 [46]/K	0	• Spindle & Mec1:1
	1	• Mec1:1 & !Spindle
	2	• Mec1:2
Rad53 [46]/K	0	• Spindle & (!DNA_Damage | DNA_Damage:1)
	1	• DNA_Damage:1 & !Spindle
	2	• DNA_Damage:2
lnc_10883		Input
lnc_9136	0	• !G2_proteins
	1	• G2_proteins:1
	2	• G2_proteins:2
	3	• G2_proteins:3

References

1. Naldi A, Berenguier D, Fauré A, Lopez F, Thieffry D, Chaouiya C. Logical modelling of regulatory networks with GINsim 2.3. Biosystems. 2009;97: 134–139. doi:10.1016/j.biosystems.2009.04.008
2. Nash RS, Volpe T, Futcher B. Isolation and characterization of WHI3, a size-control gene of Saccharomyces cerevisiae. Genetics. 2001;157: 1469–80. Available: http://www.ncbi.nlm.nih.gov/pubmed/11290704
3. Wäsch R, Cross FR. APC-dependent proteolysis of the mitotic cyclin Clb2 is essential for mitotic exit. Nature. 2002;418: 556–562. doi:10.1038/nature00856
4. Giaever G, Chu AM, Ni L, Connelly C, Riles L, Véronneau S, et al. Functional profiling of the Saccharomyces cerevisiae genome. Nature. 2002;418: 387–91. doi:10.1038/nature00935
5. Liu H, Wang Y. The function and regulation of budding yeast Swe1 in response to interrupted DNA synthesis. Mol Biol Cell. 2006;17: 2746–56. doi:10.1091/mbc.e05-11-1093
6. Fauré A, Naldi A, Lopez F, Chaouiya C, Ciliberto A, Thieffry D. Modular logical modelling of the budding yeast cell cycle. Mol Biosyst. 2009;5: 1787–1796. doi:10.1039/b910101m
7. Mai B, Miles S, Breeden LL. Characterization of the ECB Binding Complex Responsible for the M/G 1 -Specific Transcription of CLN3 and SWI4 . Mol Cell Biol. 2002;22: 430–441. doi:10.1128/mcb.22.2.430-441.2002
8. MacKay VL, Mai B, Waters L, Breeden LL. Early Cell Cycle Box-Mediated Transcription of CLN3 and SWI4 Contributes to the Proper Timing of the G 1 -to-S Transition in Budding Yeast . Mol Cell Biol. 2001;21: 4140–4148. doi:10.1128/mcb.21.13.4140-4148.2001
9. De Bruin RAM, McDonald WH, Kalashnikova TI, Yates J, Wittenberg C. Cln3 activates G1-specific transcription via phosphorylation of the SBF bound repressor Whi5. Cell. 2004;117: 887–898. doi:10.1016/j.cell.2004.05.025
10. Wijnen H, Landman A, Futcher B. The G1 Cyclin Cln3 Promotes Cell Cycle Entry via the Transcription Factor Swi6. Mol Cell Biol. 2002;22: 4402–4418. doi:10.1128/mcb.22.12.4402-4418.2002
11. Mendenhall MD, Hodge AE. Regulation of Cdc28 Cyclin-Dependent Protein Kinase Activity during the Cell Cycle of the Yeast Saccharomyces cerevisiae . Microbiol Mol Biol Rev. 1998;62: 1191–1243. doi:10.1128/mmbr.62.4.1191-1243.1998
12. Dirick L, Böhm T, Nasmyth K. Roles and regulation of Cln-Cdc28 kinases at the start of the cell cycle of Saccharomyces cerevisiae. EMBO J. 1995;14: 4803–4813. doi:10.1002/j.1460-2075.1995.tb00162.x
13. Sidorova J, Breeden L. Analysis of the SWI4/SWI6 protein complex, which directs G1/S-specific transcription in Saccharomyces cerevisiae. Mol Cell Biol. 1993;13: 1069–1077. doi:10.1128/mcb.13.2.1069-1077.1993
14. Epstein CB, Cross FR. CLB5: A novel B cyclin from budding yeast with a role in S phase. Genes Dev. 1992;6: 1695–1706. doi:10.1101/gad.6.9.1695
15. Kishi T, Ikeda A, Koyama N, Fukada J, Nagao R. A refined two-hybrid system reveals that SCFCdc4-dependent degradation of Swi5 contributes to the regulatory mechanism of S-phase entry. Proc Natl Acad Sci U S A. 2008;105: 14497–14502. doi:10.1073/pnas.0806253105
16. Visintin R, Craig K, Hwang ES, Prinz S, Tyers M, Amon A. The phosphatase Cdc14 triggers mitotic exit by reversal of Cdk-dependent phosphorylation. Mol Cell. 1998;2: 709–718. doi:10.1016/S1097-2765(00)80286-5

17. Berset C, Griac P, Tempel R, La Rue J, Wittenberg C, Lanker S. Transferable Domain in the G 1 Cyclin Cln2 Sufficient To Switch Degradation of Sic1 from the E3 Ubiquitin Ligase SCF Cdc4 to SCF Grr1 . Mol Cell Biol. 2002;22: 4463–4476. doi:10.1128/mcb.22.13.4463-4476.2002
18. Feldman RMR, Correll CC, Kaplan KB, Deshaies RJ. A Complex of Cdc4p, Skp1p, and Cdc53p/Cullin Catalyzes Ubiquitination of the Phosphorylated CDK Inhibitor Sic1p* physiological ubiquitination reactions require E3s, and whether all E3s will participate directly in both substrate. Cell. 1997;91: 221–230.
19. Cross FR, Schroeder L, Bean JM. Phosphorylation of the Sic1 inhibitor of B-type cyclins in Saccharomyces cerevisiae is not essential but contributes to cell cycle robustness. Genetics. 2007;176: 1541–1555. doi:10.1534/genetics.107.073494
20. Shirayama M, Attila T, Galova M, Nasmyth K. APC Cdc20 promotes exit from mitosis by destroying the anaphase inhibitor. Nature. 1999;402: 203–207.
21. Zou L, Mitchell J, Stillman B. CDC45, a novel yeast gene that functions with the origin recognition complex and Mcm proteins in initiation of DNA replication. Mol Cell Biol. 1997;17: 553–563. doi:10.1128/mcb.17.2.553
22. Sheu YJ, Stillman B. Cdc7-Dbf4 Phosphorylates MCM Proteins via a Docking Site-Mediated Mechanism to Promote S Phase Progression. Mol Cell. 2006;24: 101–113. doi:10.1016/j.molcel.2006.07.033
23. Devault A, Vallen EA, Yuan T, Green S, Bensimon A, Schwob E. Identification of Tah11/Sid2 as the ortholog of the replication licensing factor Cdt1 in Saccharomyces cerevisiae. Curr Biol. 2002;12: 689–694. doi:10.1016/S0960-9822(02)00768-6
24. Piatti S, Böhm T, Cocker JH, Diffley JFX, Nasmyth K. Activation of S-phase-promoting CDKs in late G1 defines a "point of no return" after which Cdc6 synthesis cannot promote DNA replication in yeast. Genes Dev. 1996;10: 1516–1531. doi:10.1101/gad.10.12.1516
25. Tanabe M, Kanehisa M. Using the KEGG database resource. Curr Protoc Bioinforma. 2012; 1–54. doi:10.1002/0471250953.bi0112s38
26. Leatherwood J. Emerging mechanisms of eukaryotic DNA replication initiation. Curr Opin Cell Biol. 1998;10: 742–748. doi:10.1016/S0955-0674(98)80117-8
27. Lee DG, Bell SP. Architecture of the yeast origin recognition complex bound to origins of DNA replication. Mol Cell Biol. 1997;17: 7159–7168. doi:10.1128/mcb.17.12.7159
28. Signon L. Genetic evidence for roles of yeast mitotic cyclins at single-stranded gaps created by DNA replication. G3 Genes, Genomes, Genet. 2018;8: 737–752. doi:10.1534/g3.117.300537
29. Spellman PT, Sherlock G, Zhang MQ, Iyer VR, Anders K, Eisen MB, et al. Comprehensive identification of cell cycle-regulated genes of the yeast Saccharomyces cerevisiae by microarray hybridization. Mol Biol Cell. 1998;9: 3273–3297. doi:10.1091/mbc.9.12.3273
30. Tóth A, Ciosk R, Uhlmann F, Galova M, Schleiffer A, Nasmyth K. Yeast cohesin complex requires a conserved protein, Eco1p(Ctf7), to establish cohesion between sister chromatids during DNA replication. Genes Dev. 1999;13: 320–333. doi:10.1101/gad.13.3.320
31. Santos A, Wernersson R, Jensen LJ. Cyclebase 3.0: A multi-organism database on cell-cycle regulation and phenotypes. Nucleic Acids Res. 2015;43: D1140--D1144. doi:10.1093/nar/gku1092
32. Peter M, Gartner A, Horecka J, Ammerer G, Herskowitz I. FAR1 links the signal transduction pathway to the cell cycle machinery in yeast. Cell. 1993;73: 747–

760. doi:10.1016/0092-8674(93)90254-N
33. Koch C, Nasmyth K. Cell cycle regulted transcription in yeast. Curr Opin Cell Biol. 1994;6: 451–459. doi:10.1016/0955-0674(94)90039-6
34. Booher RN, Deshales RJ, Kirschner MW. Properties of Saccharomyces cerevisiae wee 1 and its differential regulation of p34(CDC28) response to G1 and G2 cyclins. EMBO J. 1993;12: 3417–3426. doi:10.1002/j.1460-2075.1993.tb06016.x
35. Kaldis P, Sutton A, Solomon MJ. The Cdk-activating kinase (CAK) from budding yeast. Cell. 1996;86: 553–564. doi:10.1016/S0092-8674(00)80129-4
36. Cross FR. Two Redundant Oscillatory Mechanisms in the Yeast Cell Cycle. Dev Cell. 2003;4: 741–752.
37. Asano S, Park JE, Sakchaisri K, Yu LR, Song S, Supavilai P, et al. Concerted mechanism of Swe1/Wee1 regulation by multiple kinases in budding yeast. EMBO J. 2005;24: 2194–2204. doi:10.1038/sj.emboj.7600683
38. Okuzakil D, Watanabe T, Tanaka S, Nojima H. The Saccharomyces cerevisiae bud-neck proteins Kcc4 and Gin4 have distinct but partially-overlapping cellular functions. Genes Genet Syst. 2003;78: 113–126. doi:10.1266/ggs.78.113
39. Rock JM, Amon A. The FEAR network. Curr Biol. 2009;19: 1–10. doi:10.1016/j.cub.2009.10.002
40. Chen RH, Brady DM, Smith D, Murray AW, Hardwick KG. The spindle checkpoint of budding yeast depends on a tight complex between the Mad1 and Mad2 proteins. Mol Biol Cell. 1999;10: 2607–2618. doi:10.1091/mbc.10.8.2607
41. Visintin C, Tomson BN, Rahal R, Paulson J, Cohen M, Taunton J, et al. APC/C-Cdh1-mediated degradation of the Polo kinase Cdc5 promotes the return of Cdc14 into the nucleolus (Genes and Development (2008) 22 (79-90)). Genes Dev. 2008;22: 1560. doi:10.1101/gad.1601308.phosphatase
42. Dumitrescu TP, Saunders WS. The FEAR Before MEN: networks of mitotic exit. Cell Cycle. 2002;1: 304–307. doi:10.4161/cc.1.5.147
43. Hu F, Wang Y, Liu D, Li Y, Qin J, Elledge SJ. Regulation of the Bub2/Bfa1 GAP complex by Cdc5 and cell cycle checkpoints. Cell. 2001;107: 655–665. doi:10.1016/S0092-8674(01)00580-3
44. Geymonat M, Spanos A, Smith SJM, Wheatley E, Rittinger K, Johnston LH, et al. Control of mitotic exit in budding yeast: In vitro regulation of Tem1 GTPase by Bub2 and Bfa1. J Biol Chem. 2002;277: 28439–28445. doi:10.1074/jbc.M202540200
45. Lee SE, Frenz LM, Wells NJ, Johnson AL, Johnston LH. Order of function of the budding-yeast mitotic exit-network proteins Tem1, Cdc15, Mob1, Dbf2, and Cdc5. Curr Biol. 2001;11: 784–788. doi:10.1016/S0960-9822(01)00228-7
46. Chen Y, Sanchez Y. Chk1 in the DNA damage response: Conserved roles from yeasts to mammals. DNA Repair (Amst). 2004;3: 1025–1032. doi:10.1016/j.dnarep.2004.03.003
