# Supplementary material for: LncRNAs of Saccharomyces cerevisiae bypass the cell cycle arrest imposed by ethanol stress
Source: PLoS Comput Biol. 2022 May 19;18(5):e1010081. doi: 10.1371/journal.pcbi.1010081 (PMC9232138; doi:10.1371/journal.pcbi.1010081)
Supplement: S2 Table — The procedure used to simulate each mutant is presented in the column ’GINsim input’. (PDF) [file pcbi.1010081.s006.pdf]

S2 Table: List of mutations used for model adjustment and validation. The procedure used to simulate each mutant is presented in the column 'GINsim input'.

Mutation	Model prediction	Literature description	Reference	GINsim input
Alpha-factor	Arrest in G1	Arrest in G1	[1]	Fixed input Mating
Bfa1_Bub2-Δ	Viable	Viable	[2]	Bfa1_Bub2 KO
Bfa1_Bub2-Δ-nocodazole	Arrest in M	Viable	[3]	Bfa1_Bub2 KO + Fixed input nocodazole
Bfa1_Bub2-Δ|Mad2-Δ-nocodazole	Viable	Viable	[2]	Bfa1_Bub2 KO, Mad1_Mad2 KO + Fixed input nocodazole
Bfa1_Bub2-Δ|Pds1-Δ – nocodazole	Viable	Viable	[2]	Bfa1_Bub2 KO, Pds1 KO + Fixed input nocodazole
Cdc14-GAL	Arrest in G2	Inviable	[4]	Cdc14 [Dbf2_Mob1@3]
Cdc14-ts|Cdh1-Δ	Arrest in M	Arrest in M	[5]	APCC_Cdh1 KO, Cdc14 KO
Cdc14-ts|Cln1_2-GAL	Arrest in M	Inviable	[5]	Cdc14 KO, Cln1_2 [SCB@3]
Cdc14-ts|Cln1_2-Δ|Cln3-Δ|Cln3-GAL	Arrest in G1	Inviable	[6]	Cdc14 KO, Cln3 KO, Cln1_2 KO, Cln3 [ECB@3]
Cdc14-ts|Sic1-GAL	Viable	Arrest in M	[7]	Sic1 [Swi5@3], Cdc14 KO
Cdc14-ts|Sic1-Δ	Arrest in M	Arrest in M	[5]	Sic1 KO, Cdc14 KO
Cdc14-Δ	Arrest in M	Arrest in M	[4]	Cdc14 KO
Cdc15-ts|Tem1-MC	Arrest in M	Arrest in M	[7].	Tem1 [G2_proteins@3], Cdc15 KO
Cdc15-Δ	Arrest in M	Arrest in M	[7]	Cdc15 KO
Cdc20-ts|Bub2-Δ	Arrest in M	Arrest in M	[8]	APCC_Cdc20 KO, Bfa1_Bub2 KO
Cdc20-ts|Esp1-GAL	Arrest in G2	Arrest in M	[9]	APCC_Cdc20 KO, Esp1 [G2_proteins@3]
Cdc20-ts|Mad2-Δ	Arrest in M	Arrest in M	[6,8]	APCC_Cdc20 KO, Mad1_Mad2 KO
Cdc20-ts|Net1-ts	Viable	Inviable	[10]	APCC_Cdc20 KO, Net1 KO
Cdc20-Δ	Arrest in M	Arrest in M	[11]	APCC_Cdc20 KO
Cdc20-Δ |Pds1-Δ|Clb5_6-Δ	Viable	Viable	[12]	APCC_Cdc20 KO, Pds1 KO, Clb5_6 KO
Cdc20-Δ|Clb1_2-GAL	Arrest in M	Arrest in M	[13]	APCC_Cdc20 KO, Clb1_2[MCB@3]
Cdc20-Δ|Clb5_6-dbΔ|Pds1-Δ	Viable	Viable	[12]	APCC_Cdc20 KO, Pds1 KO, Clb5_6[APCC_Cdc20@0]
Cdc20-Δ|Clb5_6-Δ	Arrest in M	Arrest in M	[12]	APCC_Cdc20 KO, Clb5_6 KO
Cdc20-Δ|Pds1-Δ	Viable	Inviable	[12]	APCC_Cdc20 KO, Pds1 KO
Cdc45-Δ	Arrest in S	inviable	[14]	Cdc45 KO
Cdc5-Δ	Arrest in G2	Inviable	[15]	Cdc5 KO
Cdc6-Δ	Arrest in S	Arrest in S	[16]	Cdc56 KO
Cdc6-Δ|ARS-MC	Viable	Viable	[17]	Cdc6 KO, ARS[pre_RC@3]
Cdc6(2-49)	Viable	Viable	[18]	Cdc6[Clb5_6@0]
Cdh1-GAL	Arrest in G2	Arrest in G2	[19]	APCC_Cdh1[Cdc14@3]
Cdh1-Δ	Viable	Viable	[20]	APCC_Cdh1 KO

Cdh1-Δ|Cdc20-GAL|Sic1-Δ	Arrest in G2	Viable	[13]	APCC_Cdc20[G2_proteins@3], APCC_Cdh1 KO, SIc1 KO
Cdh1-Δ|Cdc20-Δ	Arrest in M	Arrest in M	[13]	APCC_Cdc20 KO, APCC_Cdh1 KO
Cdh1-Δ|Cdc20-Δ|Cdc6(2-49)|Sic1-Δ	Arrest in M	Viable	[6]	APCC_Cdh1 KO, APCC_Cdc20 KO, Cdc6[Clb5_6@0], Sic1 KO
Cdh1-Δ|Cdc20-Δ|Clb1_2-GAL	Arrest in M	Inviable	[6]	APCC_Cdh1 KO, APCC_Cdc20 KO, Clb1_2[MCB@3]
Cdh1-Δ|Cdc20-Δ|Cln1_2-Δ|Cln3-Δ	Arrest in G1	Inviable	[6]	APCC_Cdh1 KO, APCC_Cdc20 KO, Cln1_2 KO, Cln3 KO
Cdh1-Δ|Cdc20-Δ|Sic1-MC	Arrest in M	Viable	[13]	APCC_Cdh1 KO, APCC_Cdc20 KO, Sic1[Swi5@3]
Cdh1-Δ|Cdc6(2-49)	Viable	Viable	[21]	APCC_Cdh1 KO, Cdc6[Clb5_6@0]
Cdh1-Δ|Cdc6(2-49)|Sic1-Δ	Arrest in M	Arrest in M	[22]	APCC_Cdh1 KO, Sic1 KO, Cdc6[Clb5_6@0]
Cdh1-Δ|Clb1_2-GAL	Arrest in M	Arrest in M	[13]	APCC_Cdh1 KO, Clb1_2[MCB@3]
Cdh1-Δ|Clb5_6-GAL	Viable	Viable	[6]	APCC_Cdh1 KO, Clb5_6[MCB@3]
Cdh1-Δ|Cln1_2-Δ	Viable	Viable	[23]	APCC_Cdh1 KO, Cln1_2 KO
Cdh1-Δ|Cln1_2-Δ|Cln3-Δ	Arrest in G1	Inviable	[24]	APCC_Cdh1 KO, Cln1_2 KO, Cln3 KO
Cdh1-Δ|Cln1_2-Δ|Sic1-GAL	Arrest in S	Inviable	[24]	APCC_Cdh1 KO, Sic1[Swi5@3], Cln1_2 KO
Cdh1-Δ|Cln1_2-Δ|Sic1-GAL|Cln1_2-GAL	Arrest in S	Inviable	[24]	Cln1_2[SCB@3], APCC_Cdh1 KO, Sic1[Swi5@3], Cln1_2 KO
Cdh1-Δ|Swi5-Δ	Arrest in M	Arrest in M	[22]	APCC_Cdh1 KO, Swi5 KO
Clb1_2-dbΔ	Arrest in M	Arrest in M	[25]	Clb1_2[APCC_Cdh1@0], Clb1_2[APCC_cdc20@0]
Clb1_2-dbΔ|Clb5_6-Δ	Arrest in M	Arrest in M	[13]	Clb1_2[APCC_Cdh1@0], Clb1_2[APCC_cdc20@0], Clb5_6 KO
Clb1_2-GAL|Sic1-Δ	Arrest in M	Arrest in M	[26]	Clb1_2[MCB@3], Sic1 KO
Clb1_2-GAL|Swi5-Δ	Arrest in M	Arrest in M	[26]	Clb1_2[MCB@3], Swi5 KO
Clb1_2-Δ	Arrest in G2	Arrest in G2	[27]	Clb1_2 KO
Clb1_2-Δ|Pds1-Δ	Arrest in G2	Inviable	[12]	Pds1 KO, Clb1_2 KO
Clb5_6-dbΔ	Viable	Viable	[25]	Clb5_6[APCC_Cdc20@0]
Clb5_6-dbΔ|Pds1-Δ	Viable	Viable	[25]	Pds1 KO, Clb5_6[APCC_Cdc20@0]
Clb5_6-dbΔ|Sic1-Δ	Viable	Inviable	[25]	Sic1 KO, Clb5_6[APCC_Cdc20@0]
Clb5_6-GAL	Viable	Viable	[28]	Clb5_6[MCB@3]
Clb5_6-GAL|Sic1-Δ	Arrest in S	Inviable	[29]	Clb5_6[MCB@3], Sic1 KO
Clb5_6-Δ	Viable	Viable	[28]	Clb5_6 KO
Clb5_6-Δ|Cln1_2-Δ	Arrest in G2	Arrest in G1	[28]	Clb5_6 KO, Cln1_2 KO
Cln1_2-Δ	Viable	Viable	[30]	Cln1_2 KO
Cln1_2-Δ|Cln3-Δ	Arrest in G1	Inviable	[30]	Cln1_2 KO, Cln3 KO

Cln1_2-Δ|Cln3-Δ|Clb5_6-GAL	Viable	Viable	[28]	Cln1_2 KO, Cln3 KO, Clb5_6[MCB@3]
Cln1_2-Δ|Cln3-Δ|Clb5_6-MC	Viable	Viable	[31]	Cln1_2 KO, Cln3 KO, Clb5_6[MCB@3]
Cln1_2-Δ|Cln3-Δ|Cln1_2-GAL	Viable	Viable	[32]	Cln1_2 KO, Cln3 KO, Cln1_2 E3
Cln1_2-Δ|Cln3-Δ|Sic1-Δ	Viable	Viable	[30]	Cln1_2 KO, Cln3 KO, Sic1 KO
Cln1_2-Δ|Cln3-Δ|Whi5-Δ	Arrest in G1	Inviable	[6]	Cln1_2 KO, Cln3 KO, Whi5 KO
Cln1_2-Δ|Sic1-GAL	Arrest in G2	Inviable	[23]	Cln1_2 KO, Sic1[Swi5@3]
Cln1_2-Δ|Sic1-GAL|Cln1_2-GAL	Viable	Viable	[23]	Sic1[Swi5@3], Cln1_2 KO, Cln1_2 E3
Cln1_2-Δ|Sic1-Δ	Viable	Viable	[30]	Cln1_2 KO, Sic1 KO
Cln3-GAL	Viable	Viable	[33]	Cln3[ECB@3]
Cln3-Δ	Viable	Viable	[30]	Cln3 KO
Cln3-Δ|Whi5-Δ	Viable	Viable	[34]	Cln3 KO, Whi5 KO
Cohesin-Δ	Arrest in G2	Inviable	[35]	Cohesin KO
Dbf4-Δ	Arrest in S	Inviable	[36]	Dbf4_Cdc7 KO
Esp1-Δ	Arrest in M	Inviable	[37]	Esp1 KO
Mad2-Δ	Viable	Viable	[2]	Mad1_Mad2 KO
Mad2-Δ-nocodazole	Viable	Viable	[2]	Mad1_Mad2 KO + Fixed input nocodazole
Mad2-Δ|Pds1-Δ-nocodazole	Viable	Viable	[2]	Mad1_Mad2 KO, Pds1 KO + Fixed input nocodazole
Mec1-Δ	Viable	Viable	[38]	Mec1 KO
Mob1-Δ	Arrest in M	Inviable	[39]	Dbf2_Mob1 KO
Mps1-MC	Viable	Viable	[40]	Mps1[Unattached_Kinotochores@2]
Mps1-Δ	Viable	Viable	[40]	Mps1 KO
Net1-GAL	Arrest in M	Arrest in M	[10]	Net1[G2_proteins@3], Net1[PP2A@3]
Net1-Δ	Viable	Viable	[10]	Net1 KO
Net1-Δ-nocodazole	Viable	Viable	[10]	Net1 KO + Fixed input nocodazole
ORC-Δ	Arrest in G1	Inviable	[41]	ORC KO
Pds1-Δ	Viable	Viable	[42]	Pds1 KO
Pds1-Δ-nocodazole	Viable	Arrest in M	[42]	Pds1 KO + Fixed input nocodazole
PP2A_Cdc55-Δ	Viable	Viable	[43]	PP2A KO
Rad53-Δ	Viable	Viable	[38]	Rad53 KO
SBF-Δ	Arrest in G1	Inviable	[44]	SBF KO
SBF-Δ|MBF-Δ	Arrest in G1	Arrest in G1	[44]	SBF KO, MBF KO
SBF-Δ|Sic1-Δ	Arrest in G1	Inviable	[45]	SBF KO, Sic1 KO
Sic1-GAL	Viable	Viable	[46]	Sic1[Cdc14@3]
Sic1-GAL-dbΔ	Arrest in G2	Arrest in G1	[46].	Sic1[Cdc14@3], Sic1[SFC_Cdc4@0]
Sic1-Δ	Viable	Viable	[47]	Sic1 KO
Sic1-Δ|Cdc6(2-49)	Viable	Viable	[25]	Sic1 KO, Cdc6[Clb5_6@0]
Sic1-Δ|Cdh1-Δ	Arrest in M	Inviable	[25]	APCC_Cdh1 KO, Pds1 KO
Slk19-Δ	Arrest in M	Viable	[48]	Slk19 KO
Swe1-MC	Arrest in G2	Arrest in G2	[49]	Swe1[S_proteins@3]
Swi5-Δ	Viable	Viable	[50]	Swi5 KO
TAB6-1	Viable	Viable	[51]	Cdc14[Net1@0]
TAB6-1|Clb5_6-Δ	Viable	Arrest in G1	[51]	Cdc14[Net1@0], Clb5_6 KO
Tah11-Δ	Arrest in G1	Inviable	[52]	Tah11 KO

Tem1-GAL	Viable	Viable	[7]	Tem1 [G2_proteins@3]
Tem1-GAL|Mad2-Δ nocodazole	Viable	Viable	[2]	Mad1_Mad2 KO, Tem1 [G2_proteins@3 + Fixed input nocodazole
Whi5-Δ	Viable	Viable	[20]	Whi5 KO
Pds1-dbΔ-GAL|Esp1-ts	Arrest in M	Inviable	[53]	Esp1 KO, Pds1[G2_proteins@3], Pds1[APCC_Cdc20@0]

References

1. Schandel KA, Jenness DD. Direct evidence for ligand-induced internalization of the yeast alpha-factor pheromone receptor. Mol Cell Biol. 1994;14: 7245–7255. doi:10.1128/mcb.14.11.7245-7255.1994
2. Alexandru G, Zachariae W, Schleiffer A, Nasmyth K. Sister chromatid separation and chromosome re-duplication are regulated by different mechanisms in response to spindle damage. EMBO J. 1999;18: 2707–2721. doi:10.1093/emboj/18.10.2707
3. Hoyt MA, Totis L, Roberts BT. S. cerevisiae genes required for cell cycle arrest in response to loss of microtubule function. Cell. 1991;66: 507–517. doi:10.1016/0092-8674(81)90014-3
4. Visintin R, Craig K, Hwang ES, Prinz S, Tyers M, Amon A. The phosphatase Cdc14 triggers mitotic exit by reversal of Cdk-dependent phosphorylation. Mol Cell. 1998;2: 709–718. doi:10.1016/S1097-2765(00)80286-5
5. Yuste-Rojas M, Cross FR. Mutations in CDC14 results in high sensitivity to cyclin gene dosage in Saccharomyces cerevisiae. Mol Gen Genet. 2000;263: 60–72. doi:10.1007/PL00008676
6. Chen KC, Calzone L, Csikasz-Nagy A, Cross FR, Novak B, Tyson JJ. Integrative Analysis of Cell Cycle Control in Budding Yeast. Mol Biol Cell. 2004;15: 3841–3862. doi:10.1091/mbc.e03-11-0794
7. Jaspersen SL, Charles JF, Tinker-Kulberg RL, Morgan DO. A late mitotic regulatory network controlling cyclin destruction in Saccharomyces cerevisiae. Mol Biol Cell. 1998;9: 2803–2817. doi:10.1091/mbc.9.10.2803
8. Shirayama M, Zachariae W, Ciosk R, Nasmyth K. The Polo-like kinase Cdc5p and the WD-repeat protein Cdc20p/fizzy are regulators and substrates of the anaphase promoting complex in Saccharomyces cerevisiae. EMBO J. 1998;17: 1336–1349. doi:10.1093/emboj/17.5.1336
9. Ciosk R, Zachariae W, Michaelis C, Shevchenko A, Mann M, Nasmyth K. An ESP1/PDS1 complex regulates loss of sister chromatid cohesion at the metaphase to anaphase transition in yeast. Cell. 1998;93: 1067–1076. doi:10.1016/S0092-8674(00)81211-8
10. Visintin R, Hwang ES, Amon A. Cfi1 prevents premature exit from mitosis by anchoring Cdc14 phosphatase in the nucleolus. Lett to Nat. 1999;398: 818–823.
11. Lim HH, Goh PY, Surana U. Cdc20 is essential for the cyclosome-mediated proteolysis of both Pds1 and Clb2 during M phase in budding yeast. Curr Biol. 1998;8: 231–237. doi:10.1016/s0960-9822(98)70088-0
12. Shirayama M, Attila T, Galova M, Nasmyth K. APC Cdc20 promotes exit from mitosis by destroying the anaphase inhibitor. Nature. 1999;402: 203–207.
13. Cross FR. Two Redundant Oscillatory Mechanisms in the Yeast Cell Cycle. Dev Cell. 2003;4: 741–752.
14. Hennessy KM, Lee A, Chen E, Botstein D. A group of interacting yeast DNA replication genes. Genes Dev. 1991;5: 958–969. doi:10.1101/gad.5.6.958
15. Almawi AW, Langlois-Lemay L, Boulton S, Rodríguez González J, Melacini G, D'Amours D, et al. Distinct surfaces on Cdc5/PLK Polo-box domain orchestrate combinatorial substrate recognition during cell division. Sci Rep. 2020;10: 3379. doi:10.1038/s41598-020-60344-4
16. Borlado LR, Méndez J. CDC6: From DNA replication to cell cycle checkpoints and oncogenesis. Carcinogenesis. 2008;29: 237–243. doi:10.1093/carcin/bgm268
17. Hogan E, Koshland D. Addition of extra origins of replication to a minichromosome suppresses its mitotic loss in cdc6 and cdc14 mutants of

Saccharomyces cerevisiae. Proc Natl Acad Sci U S A. 1992;89: 3098–3102. doi:10.1073/pnas.89.7.3098
18. Nguyen VQ, Co C, Li JJ. Cyclin-dependent kinases prevent DNA re-replication through multiple mechanisms. Nature. 2001;411: 1068–1073. doi:10.1038/35082600
19. Zachariae W, Schwab M, Nasmyth K, Seufert W. Control of cyclin ubiquitination by CDK-regulated binding of Hct1 to the anaphase promoting complex. Science (80-). 1998;282: 1721–1724. doi:10.1126/science.282.5394.1721
20. Jorgensen P, Nishikawa JL, Breitkreutz BJ, Tyers M. Systematic identification of pathways that couple cell growth and division in yeast. Science (80-). 2002;297: 395–400. doi:10.1126/science.1070850
21. Calzada A, Sacristán M, Sánchez E, Bueno A. Cdc6 cooperates with Sic1 and Hct1 to inactivate mitotic cyclin-dependent kinases. Nature. 2001;412: 355–358. doi:10.1038/35085610
22. Archambaut V, Li CX, Wa¨sch R, Alan J. T, T. Chait B, P. Rout M, et al. Genetic and Biochemical Evaluation of the Importance of Cdc6 in Regulating Mitotic Exit. Mol Biol Cell. 2003;14: 5069–5081. doi:10.1091/mbc.E03
23. Cross FR, Archambault V, Miller M, Klovstad M. Testing a Mathematical Model of the Yeast Cell Cycle. Mol Biol Cell. 2002;13: 52–70. doi:10.1091/mbc.01
24. Schwab M, Lutum AS, Seufert W. Yeast Hct1 is a regulator of Cib2 cyclin proteolysis. Cell. 1997;90: 683–693. doi:10.1016/S0092-8674(00)80529-2
25. Wäsch R, Cross FR. APC-dependent proteolysis of the mitotic cyclin Clb2 is essential for mitotic exit. Nature. 2002;418: 556–562. doi:10.1038/nature00856
26. Toyn JH, Johnson AL, Donovan JD, Toone WM, Johnston LH. The Swi5 Transcription Factor of Saccharomyces cerezkiae Has a Role in Exit From Mitosis Through Induction of the cdk-Inhibitor Sic1 in Telophase. 1996;96: 85–96.
27. Surana U, Amon A, Dowzer C, McGrew J, Byers B, Nasmyth K. Destruction of the CDC28/CLB mitotic kinase is not required for the metaphase to anaphase transition in budding yeast. EMBO J. 1993;12: 1969–78. Available: http://www.ncbi.nlm.nih.gov/pubmed/8491189
28. Schwob E, Nasmyth K. CLB5 and CLB6, a new pair of B cyclins involved in DNA replication in Saccharomyces cerevisiae. Genes Dev. 1993;7: 1160–1175. doi:10.1101/gad.7.7a.1160
29. Jacobson MD, Gray S, Yuste-Rojas M, Cross FR. Testing Cyclin Specificity in the Exit from Mitosis. Mol Cell Biol. 2000;20: 4483–4493. doi:10.1128/mcb.20.13.4483-4493.2000
30. Dirick L, Böhm T, Nasmyth K. Roles and regulation of Cln-Cdc28 kinases at the start of the cell cycle of Saccharomyces cerevisiae. EMBO J. 1995;14: 4803–4813. doi:10.1002/j.1460-2075.1995.tb00162.x
31. Epstein CB, Cross FR. CLB5: A novel B cyclin from budding yeast with a role in S phase. Genes Dev. 1992;6: 1695–1706. doi:10.1101/gad.6.9.1695
32. Cross FR, Tinkelenberg AH. A potential positive feedback loop controlling CLN1 and CLN2 gene expression at the start of the yeast cell cycle. Cell. 1991;65: 875–883. doi:10.1016/0092-8674(91)90394-E
33. Tyers M, Tokiwa G, Nash R, Futcher B. The Cln3-Cdc28 kinase complex of S. cerevisiae is regulated by proteolysis and phosphorylation. EMBO J. 1992;11: 1773–1784. doi:10.1002/j.1460-2075.1992.tb05229.x
34. De Bruin RAM, McDonald WH, Kalashnikova TI, Yates J, Wittenberg C. Cln3

activates G1-specific transcription via phosphorylation of the SBF bound repressor Whi5. Cell. 2004;117: 887–898. doi:10.1016/j.cell.2004.05.025
35. Guacci V, Koshland D, Strunnikov A. A direct link between sister chromatid cohesion and chromosome condensation revealed through the analysis of MCD1 in S. cerevisiae. Cell. 1997;91: 47–57. doi:10.1016/S0092-8674(01)80008-8
36. Solomon NA, Wright MB, Chang S, Buckley AM, Dumas LB, Gaber RF. Genetic and molecular analysis ofDNA43 andDNA52: Two new cell-cycle genes inSaccharomyces cerevisiae. Yeast. 1992;8: 273–289. doi:10.1002/yea.320080405
37. Tinker-Kulbetg RL, Morgan DO. Pds1 and Esp1 control both anaphase and mitotic exit in normal cells and after DNA damage. Genes Dev. 1999;13: 1936–1949. doi:10.1101/gad.13.15.1936
38. Tercero JA, Diffley JFX. Regulation of DNA replication fork progression through damaged DNA by the Mec1/Rad53 checkpoint. Nature. 2001;412: 553–557. doi:10.1038/35087607
39. Komarnitsky SI, Chiang Y-C, Luca FC, Chen J, Toyn JH, Winey M, et al. DBF2 Protein Kinase Binds to and Acts through the Cell Cycle-Regulated MOB1 Protein. Mol Cell Biol. 1998;18: 2100–2107. doi:10.1128/mcb.18.4.2100
40. Schutz AR, Giddings TH, Jr. ES, Winey M. The Yeast CDC37 Gene Interacts with MPS1 and Is Required for Proper Execution of Spindle Pole Body Duplication The Yeast CDC37 Gene Interacts with MPS1 and Is Required for Proper Execution of Spindle Pole Body Duplication. J Cell Biol. 1997;136: 39–48.
41. Newlon CS. Two Jobs for the Origin Replication Complex. 1993;262: 17–19.
42. Yamamoto A, Guacci V, Koshland D. Pds1p, an inhibitor of anaphase in budding yeast, plays a critical role in the APC and checkpoint pathway(s). J Cell Biol. 1996;133: 99–110. doi:10.1083/jcb.133.1.99
43. Rossio V, Michimoto T, Sasaki T, Ohbayashi I, Kikuchi Y, Yoshida S. Nuclear PP2A-Cdc55 prevents APC-Cdc20 activation during the spindle assembly checkpoint. J Cell Sci. 2013;126: 4396–4405. doi:10.1242/jcs.127365
44. Koch C, Moll T, Neuberg M, Ahorn H, Nasmyth K. A role for the transcription factors Mbp1 and Swi4 in progression from G1 to S phase. Science (80-). 1993;261: 1551–1557. doi:10.1126/science.8372350
45. Wijnen H, Futcher B. Genetic analysis of the shared role of CLN3 and BCK2 at the G1-S transition in Saccharomyces cerevisiae. Genetics. 1999;153: 1131–1143. doi:10.1093/genetics/153.3.1131
46. Verma R, Annan RS, Huddleston MJ, Carr SA, Reynard G, Deshaies RJ. Phosphorylation of Sic1p by G1 Cdk required for its degradation and entry into S phase. Science (80-). 1997;278: 455–460. doi:10.1126/science.278.5337.455
47. Schneider BL, Yang Q-H, Futcher AB. Linkage of Replication to Start by the Cdk Inhibitor Sicl. 1996;272: 26–28.
48. Zeng X, Kahana JA, Silver PA, Morphew MK, Mcintosh JR, Fitch IT, et al. Slk19p Is a Centromere Protein That Functions to Stabilize Mitotic Spindles. 1999;146: 415–425.
49. Lianga N, Williams EC, Kennedy EK, Doré C, Pilon S, Girard SL, et al. A wee1 checkpoint inhibits anaphase onset. J Cell Biol. 2013;201: 843–862. doi:10.1083/jcb.201212038
50. Dohrmann PR, Butler G, Tamai K, Dorland S, Greene JR, Thiele DJ, et al. Parallel pathways of gene regulation: Homologous regulators SWI5 and ACE2 differentially control transcription of HO and chitinase. Genes Dev. 1992;6: 93–

104. doi:10.1101/gad.6.1.93

51. Shou W, Sakamoto KM, Keener J, Morimoto KW, Traverso EE, Azzam R, et al. Net1 stimulates RNA polymerase I transcription and regulates nucleolar structure independently of controlling mitotic exit. Mol Cell. 2001;8: 45–55. doi:10.1016/S1097-2765(01)00291-X
52. Jacobson MD, Muñoz CX, Knox KS, Williams BE, Lu LL, Cross FR, et al. Mutations in SID2, a novel gene in saccharomyces cerevisiae, cause synthetic lethality with sic1 deletion and may cause a defect during S phase. Genetics. 2001;159: 17–33. doi:10.1093/genetics/159.1.17
53. Cohen-Fix O, Koshland D. Pds1p of budding yeast has dual roles: Inhibition of anaphase initiation and regulation of mitotic exit. Genes Dev. 1999;13: 1950–1959. doi:10.1101/gad.13.15.1950
