# Supplementary material for: LncRNAs of Saccharomyces cerevisiae bypass the cell cycle arrest imposed by ethanol stress
Source: PLoS Comput Biol. 2022 May 19;18(5):e1010081. doi: 10.1371/journal.pcbi.1010081 (PMC9232138; doi:10.1371/journal.pcbi.1010081)
Supplement: S3 Table — Note A: General setting used to test mutations, random cell cycle perturbations, and the effects of ethanol and lncRNAs on the cell cycle. Note B: Setup of checkpoint nodes fixed at the minimum activated value. Note C: The MATING node had only the activation value ’1’, which is its maximum or minimum activated value. Note D: Setup for each checkpoint node fixed at the maximum activated value. Note E: Setup of all checkpoint nodes fixed at the minimum activated value. Note F: Setup of all checkpoint nodes fixed at the maximum activated value. (PDF) [file pcbi.1010081.s007.pdf]

S3 Table: Exploring the effect of checkpoint nodes on the cell cycle model. **Note A:** General setting used to test mutations, random cell cycle perturbations, and the effects of ethanol and lncRNAs on the cell cycle. **Note B:** Setup of checkpoint nodes fixed at the minimum activated value. **Note C:** The MATING node had only the activation value '1', which is its maximum or minimum activated value. **Note D:** Setup for each checkpoint node fixed at the maximum activated value. **Note E:** Setup of all checkpoint nodes fixed at the minimum activated value. **Note F:** Setup of all checkpoint nodes fixed at the maximum activated value.

**Unattached_kinetochores**	**Misaligned_spindle**	**Mating**	**DNA_damage**	**Functional cell cycle?**	**Note**
1	1	0	1	Yes	A
1	0	0	0	Yes	B
0	1	0	0	Yes	B
0	0	0	1	Yes	B
0	0	1	0	No	C
2	0	0	0	No	D
0	2	0	0	Yes	D
0	0	0	2	No	D
1	1	1	1	No	E
2	2	1	2	No	F
