# Supplementary material for: LncRNAs of Saccharomyces cerevisiae bypass the cell cycle arrest imposed by ethanol stress
Source: PLoS Comput Biol. 2022 May 19;18(5):e1010081. doi: 10.1371/journal.pcbi.1010081 (PMC9232138; doi:10.1371/journal.pcbi.1010081)
Supplement: S4 Table — The numbers in brackets represent the up- or downregulation profiles for each phenotype from the data reported in S5 Table and Fig 3E in the main text. According to the following definition downregulated<non-differentially expressed<upregulated, where the ’non-differentially expressed’ genes had a log2 fold-change = 0 and the differentially expressed genes must have a false discovery rate < 0.01, the nodes related to the downregulated genes were model-constrained to a value of ’0’ or ’1’, whereas the nodes related to the upregulated genes reached a value of ’3’. For instance, Rad53[0,1] constrains the node Rad53 to assume only values of ’0’ or ’1’ during simulations. The notation X[Y@n] indicates that the logical functions of the X node will operate such as the Y = ‘n’ level (the X’s node regulator). For instance, Chk1[Mec1@2] will drive the Chk1 node updates assuming Mec1 = 2, although Mec1 will be updated according to its logic equations. This approach enables to peak the maximum level for these nodes but not excluding the possibility to evolve during the simulation. The notation in the equations was designed according to the symbols and rules indicated in the GINsim manual. (PDF) [file pcbi.1010081.s008.pdf]

S4 Table: Model constraints used to simulate the effects of ethanol on the cell cycle ('*', the first experimental model simulation), the DNA damage pathways (the second experimental model simulation), and the effect of lncRNAs on the cell cycle based on the transcriptome data (the third and fourth experimental model simulations). The numbers in brackets represent the up- or downregulation profiles for each phenotype from the data reported in **S5 Table and Fig 3E** in the main text. According to the following definition $downregulated < non\text{-}differentially\ expressed < upregulated$, where the 'non-differentially expressed' genes had a log2 fold-change = 0 and the differentially expressed genes must have a false discovery rate < 0.01, the nodes related to the downregulated genes were model-constrained to a value of '0' or '1', whereas the nodes related to the upregulated genes reached a value of '3'. For instance, Rad53[0,1] constrains the node Rad53 to assume only values of '0' or '1' during simulations. The notation X[Y@n] indicates that the logical functions of the X node will operate such as the Y='n' level (the X's node regulator). For instance, Chk1[Mec1@2] will drive the Chk1 node updates assuming Mec1=2, although Mec1 will be updated according to its logic equations. This approach enables to peak the maximum level for these nodes but not excluding the possibility to evolve during the simulation. The notation in the equations was designed according to the symbols and rules indicated in the GINsim [1] manual**.**

**Simulation**	**Model constraints**	**Outcome**
S288c DNA damage	Rad53[0,1], Chk1[Mec1@2]	Arrest in M
X2180-1A DNA damage	Rad53[0,1], Chk1[Mec1@2], Mec1[DNA_Damage@2], Pds1[0,1]	Viable
BY4742 DNA damage	Rad53[0,1], Chk1[Mec1@2], Mec1[DNA_Damage@2], Pds1[0,1]	Viable
BY4741 DNA damage	Rad53[0,1], Mec1[DNA_Damage@2]	Arrest in M
SEY6210 DNA damage	Chk1[Mec1@2]	Arrest in M
*LT phenotype	Sic1[0,1], Cak1[S_proteins@3], MCM[0,1], Hsl1[0,1], Cln1_2[0,1], Gin4[0,1], Cdc14[0,1], Bub1[0,1]	Arrest in M

*HT phenotype	SCF_Cdc4[S_proteins@3], SCF_Grr1[S_proteins@3], Pds1[0,1], Cak1[S_proteins@3], MCM[0,1], Hsl1[0,1], Cln1_2[0,1], Gin4[0,1], Cdc14[0,1], Bub1[0,1]	Arrest in G1

References

1. Naldi A, Berenguier D, Fauré A, Lopez F, Thieffry D, Chaouiya C. Logical modelling of regulatory networks with GINsim 2.3. Biosystems. 2009;97: 134–139. doi:10.1016/j.biosystems.2009.04.008
