# Supplementary material for: LncRNAs of Saccharomyces cerevisiae bypass the cell cycle arrest imposed by ethanol stress
Source: PLoS Comput Biol. 2022 May 19;18(5):e1010081. doi: 10.1371/journal.pcbi.1010081 (PMC9232138; doi:10.1371/journal.pcbi.1010081)
Supplement: S5 Table — The “-” symbol indicates the lack of differential expression (the non-differentially expressed genes). (PDF) [file pcbi.1010081.s009.pdf]

S5 Table: Log2 fold-changes in the differentially expressed genes related to the cell cycle used here. The "-" symbol indicates the lack of differential expression (the non-differentially expressed genes).

**Gene**	**BMA64-1A**	**BY4742**	**X2180-1A**	**SEY6210**	**BY4741**	**S288C**
YLR079W (Sic1)	-	-	-	-1.08117595	-1.22444222	-1.0492007
YDR328C (Skp1 - SCF)	0.661474217	0.515904744	0.893148145	-	-	-
YDR113C (Pds1)	-1.26114795	-0.86942896	-0.39082185	-	-	-
YFL029C (Cak1)	0.652247841	1.388448777	0.43798135	2.13350926	1.575399232	1.732428162
YBL023C (Mcm2 - MCM)	-0.36072788	-1.29496158	-1.08464273	-1.19079481	-2.6943636	-0.82771961
YKL101W (Hsl1)	-1.30582079	-1.21648815	-0.75315376	-1.09028362	-3.12464438	-0.97596056
YMR199W (Cln1)	-1.51163272	-3.1838803	-2.01137073	-2.50849942	-0.73793939	-2.15737657
YDR507C (Gin4)	-0.95438996	-1.76815401	-1.45276723	-1.68325044	-1.53158578	-1.82844616
YFR028C (Cdc14)	-0.59305398	-2.02320133	-1.41824222	-2.4116205	-1.57427086	-1.68677962
YGR188C (Bub1)	-0.82833276	-1.4003396	-0.86156941	-0.94239787	-2.34407248	-0.93849007
