# Supplementary material for: LncRNAs of Saccharomyces cerevisiae bypass the cell cycle arrest imposed by ethanol stress
Source: PLoS Comput Biol. 2022 May 19;18(5):e1010081. doi: 10.1371/journal.pcbi.1010081 (PMC9232138; doi:10.1371/journal.pcbi.1010081)
Supplement: S1 Data — Name; strain: genomic coordinates start-end; orientation. (PDF) [file pcbi.1010081.s010.pdf]

S1 Data: Sequences of lncRNAs studied here in FASTA format. Name; strain: genomic coordinates start-end; orientation.

>lnc9136; SEY6210: 84,821-86,676; negative

ATCTTTTTTTCTTGGTTCAGTTTTGACTCTTCCACACACCCATTTCGCTAGCGTAGAACAAGGGGAGACACAAACTTTCTTTTCCTTGCAAGTTAATAACACAA
CACCCGTCCTCCTGCACTGAATTTACACAGAAGTTAAGAACCGCCTCTGCTTTTCTGGTATTATTTTGCAGCCTTGCCTAAAAAAAGTGTGGTAATTTTTGAGAA
TAATATATTACTGTGTTTTTCGAAACGAAAGTTCTGAATATTGGCTTGGTCGCTTCCTGATATCTGGAAACTTGTTTAATGTTTTTAATTATCAGTCTTCAAAGCG
TCCTGATATGACTTGCCACGTTTCCATCCATTCTATATGTAGTTTAAATACAATGAGATAAAGGGTGCCTGCATGGAACTTTCCATTCGAAAAAAGAGATCATA
AACCTTATCTGTCAAATAAAAGCCAATCTATTTGATTAGGTAAAAAAAGTGATGTCAGACCAAAGTGCCCCTCTCCTGCAATGATCATCGATTACGTACGGGTG
TAATGATCACGACAGTAAAGTGAGCAAGGTAACTACTATCCCTAAGTGCGGCGTCATTGTAAGTCTACGACGTAGCATATTCAGTTTACTGTACAAGAAAAGT
TTTCTCTCTGTAACAAGAGGAAATTGGTTAGCTTTCTACGCTTACTTCGTTTGGTACGATCAGTAAAGGTGAATAAATTAAGATCCACGCCTTCATATGGCAGG
ATCCACGATATTCCTTGCAATACCCCTGGATAATTCAAAAACCAAATGTTACACGGTATACTTGAAAATCGATACCATATTAGGAACACACCGGCCAAAGGTT
GATAATACTTGGTATGAGATAAAACCCGAGCTCAAGGTTTAATGTTAAAATTTTTTGTATATATATATATATAGTAATGGGCTCGGTTGCAAATTTTTATGACTA
TCTTGAAACTAGTTGAAATTGCAAGAACCGTCACTCTATGGAGTACATGAACAACTAAAAAATAGCTTGAAGATACTTGCCACTCCATGCCATTTCTAGAAAG
GGGTCTCTTGAGAAATGTGTGTTAAAATAACGGTTATATAGTTGGAAAGAATATTACTATTTTGCGATTAAGGGATGCCGTATGGCAGGCAAGGAAGTATTTG
ATCAAGAATACCGATTAATGAAGCATGTTTGCCATTATCGTTGAACCTTGGTATTTCTCAGGTTTCCTTATCGATACGATAGGAATGAGAATCAACTAACATTT
ATCGACTACAATTCATTATTACTACTATATTATCAAATGCTCTGCAAGGAGATGACCAAAAAGAAATGAGAAACAGTCTTCATCTCAAAATATAGGCAATTACC
AGGTATACGATATTTCCTCAAAATGAAAATGCCTAAAATACGACAGAAAAACTTAGGGTGACGACTTTTTAACTAATCACTGAATGCAGCCGAAGCAAGTAAT
TATTTCCCTCGTTGCTACTCATTGAGGCCGCTCCATATGGAGATTTGAAAAAGGTTCTATTGATTCTTTAACTGGTGTCCATCCCCATGAGAACGTTTTAGTTAA
TTAACTGCTGGTAAAATAAGCGATAGTTGGTATTTTCAAAGGAATATGAGAAATGCGGGAATACTAGTTAGTAGACTATATTAAAACTTTGTTTCAACAGAATA
ATATATAGATACAACAGGGCAGTTGTGAAGATTGGCAAATAAATCTTTAAAATAACTTCTTTGTCCGGGCTAAGCAGGATACCACACTTTAAATGATGATTACC
ACCCCACAAGAGAAGTCGGTCTGAATTTATTAATTCTGGTATCAGCAAATCCCGTATTTCAATAACTTCAAGAAATCTTGTTCCTCGTGG

>lnc10883; BY4742: 13,912-17,479; negative

TATATAGCATAAACCGACAATAATGCGTGGGTGGTCCTCCATGCCGAATTTGTGAACTGAATTATTATCTAAGCACTGGATCGATCACCTTAAGATGATATATT
ATTTATAACTAAAACGCAGCAGCATTACACTCCAGAGTAACTCCTGAATTTCGTCATGGCATGTACTTATACTATATAACCAACAAAGGATGACTGAATAAGA
ACCAAATACTTTTCAAGAGATAAGATACGCGTTGCCATTGTTCGCAAGGCAGATCAAGAAGCTTCCTAGGCCTGTGAAAAAAGGAGGAAAAAAGAAATGAAT
TTTCTAGCTGTATTTAGATTGGATTTGAGGTTCACTAGTATAAGCAACAGCTCCAGTTCTTTAGTCAAGTGCCAGCTTCCCTTGAAATATTAACCTCCCTTTTTTC
TTCGTAAACCACTATTATGACCATCGGCCTGTTAATAAGGAGAAAAATAGAAAACTGAAGCAATAGCGGTATCATAAAGCTGACCGTGTAAAGTCACCACTAA
CCTCCCAATAAACTCTTCCAACTCTGTTTTTTTCAAAGCTAGTGGTGCATTTGAACGCTATTTAGGATTCCGATCTCTTCAGAAACATGCTGATTTAGAAGTAGT
CAAATTACTTATAGGCGTGAATTTCGAAATTGATCTCTTAAGCAGAATTTAGTTGTATCAAACTATATTCGTTTTGGAGCGCATATACTTTACATTTCGATGCGT
TTAACGTTGCCTCTGTTCTTTTCCTATTAATTCTATTAGATAAGTTTATTTCCATCTCATATAAAGAAGAACCTGTCGAGGTTATATGATCTCTTTTCCGTCTTTTT
ACCGATTCTGGTTAGTGTATGCTTCATTTGAGTTTCTTGCACTGCTTTGCCGACCAAATATGGAAATCATCTATTTTAACTCTATTATTGAAGAGGCTTTTCCTGG
CACATATAAATATGAATATAAATGTTGTGCGGCTGTAATATTTGGCTAGAATTACCAGCCTGATTGCGGGTTATATATTGCCTTCTTTTGGACATTTACTCCCGG
TGTAATGTTGGCAGCAGTAGTACCTATACCTGTGTGCCACCCGTTTGGATATTACTATAACTTCAAAATATGGGAGGATTACTTTCTCGAAAGTTGCTAAAAAA
ATCATTCGGGAATTTTCGCTACAGATTAATCAAATGTAGCTCGACAGTCTTCATATTATGAGTTTAAAAGTTCTTTAATATTCTTGTACATCTCAATTGTTATGTC
AACCAATTACCAGTTAAAAAGAGAGAAATTTTATGAGTTATTAGATGATTATGCGGTGCAGAACATTGAGGTTCCTCGTGATAATGATAATACGCACAGTTAG
AGAGCAAGTTAGAAAGCAGATTAGAGAGGGACAAGAAAGATAATATCTTCAGTTCTGTAAGGCTACTTTTAAACCAAATGAGCCAGAAGACTTCTCTATTCGT
GAGCGTCCCAAAAGTTATGGTCATTGCCAGTGGAGACGAAGCTGTACTTTTTACTGCTTCACCTGTGTTTTATATAGATAGCTCATAAAGAGATATTGACACCA
AACTCAAACGAAAGCTTACAATTCCGCATCAGCATTGAGACCCTATATTCCCCTCATCTATTCCAAATAAGAATCATATAAAGAATTCACTAGGCACAGATAAC
TGCTGATTTATAGACATAATCCCTTCGCATCCACGTTAGCATGATTGGTGACACGCTTTCTGAGCATTAGCTTGCGATAGGTTTCCAGCATCTCCAGTTACAATC
ATGGATAAATACACAAGGCGACGTTCAAACATGACTGAGGAAATGCAGTATTTAAAGCTTACTCTTTCCTCAGTTGAGTACGGTAAAATCGATTAACAAGCCA
TTTCAATAGTTATAATTTTTTTTTTTGGTCATGGAAGACCTGAACTAAAGTGTTTTAGTAAACCAATTGGAGTGAGAGTTTTTCATTCCGAAGATTCTTTATCTCA
AAATTTCTTTATCGAAAGACACTTCTGTGTCACTGTCCGTTCAATCAGTCAGATAGTTCCAACTCCGATGTCTTCCAATACCTCAACGAAGACCGAAAAATAAA
AGGTTTGTTTGACGGAGTGTGTTGATTAGTGCATTGGTGACGTGGGGTAGCAAAATCCAGATACTTCTATTTTTTGAAAAAGAAAAGAGAGAGTGCTAGAATG
TTTTCACGTTTATCAGTACACGAAAAACAAAACCTGAAGCAAATGATTACCATAACTATTGTCCACTTATGGGGAAGTTGCTAAAAATAACACATTATTTACTA
AGGGAACACAAATTGCTCATAGTATACTTGACTTTTTTTACTTAACTTTTGCAGCGATTGGTGATGAAATGTTTCAAAAAAAAAAAAAAAATCGAAAATCCATT
GTATTGCATAAGAAATGGCGAATTTTAGGCACGAAATTAACTTACATCTCTCCATTCCTTTCCTCATGAAAGAACCGTTATGTTAGCTAGCCCTGAACTTCTTAT
TTTGGAAGGACCCTCACCTCAATTTTCAAATGAACCGCGGACTTCACTTTCTACGGTCGTTTCTTCAAAAATCTTTACAACGTATATCGCCTGTTGCATTGCCTTT
TTCGGAAAACCTTATTTTACGACAAACCTTTTTTAGGATTAAAGTTTAAAAAATTTCTTCGTCTGCCTCAAAATTGCGCAGCTTTAAGCTTCCAGAATGTTTTAC
ACGAAATACCCTAGCGCTGCATTTCTTTTTTTCCTCAGTTGCGGTAGTAGAAAAGTATTACATCAGCACGAGTAACAACTTATGGTGTCAGTTTCATAATTTCAC
GCACTAAAGTTAATTTCCCCTTTTGTTCAAAAACTTTCAGGAGGTGTGGCCATGATGGACAGAAAGAATGTTATGCTTTAGGATTATTGTACAATTATTTAGATT
TGCTAAAGCGCGTCTTATAGTAATCGTGCAGTAAATTTTGTAATTCAGTTCCAGTTCACTTTATCATGATAGTGTTTTATCAGTTTCGAATTTTTGGCACACGAA
AGCTTGTTTTCCTTTATAATTCTTTTCAGGAATTATACCTTACTTCAATTTAGAAGCGCATAGCAAACGGGAAGAATGCGTACACTGCAGGGTTGTTGTGATCCG
TCACGTGTATGCAAAACGCGTTAGAGAGTAGTTATGTGCTAACTGTGTGGTAGAAATTTATATTTCTCTTTTAAGGTTGTTTGGTATGTATCCGCCGTGAAAAAA
GTAAGAAAACATCCAAATGGGCTCCTGCCTGAATGTTTCCTTCGATAAACGTATACGCCAATAGTGAAGAAAGTCGCCTAAATTTATTCTGTTGTAACGAATGA
TTCATGGTTTAACTTTTTTAGACTCTAGTATAACTAGCAAGTATACATATTTGCTCAAAACATGTTCGAGTGCGAAAGTGTTGACATATAAAGAGACACTTCTTT
TACGTTCCACTGTTTCGAGTTTACGTTGAAGATTTGTTTTAGGGTGCTTAATCAAAGAACAACAAATAAAAAATGCCTGTGGCTGCTCGATATATATTTTTGACC
GGCCTATTTTTGCTGGGTTTGGTTTCATGCTAGCGTTTGGTGACTTAAGTTTAGTTCCATTCACCTACTCATTACAAGCGCGTTACTT
